# Deprogramming metabolism in pancreatic cancer with a bi-functional GPR55 inhibitor and biased β_2_ adrenergic agonist

**DOI:** 10.1038/s41598-022-07600-x

**Published:** 2022-03-07

**Authors:** Artur Wnorowski, Danuta Dudzik, Michel Bernier, Jakub Wójcik, Guido Keijzers, Alberto Diaz-Ruiz, Karolina Mazur, Yongqing Zhang, Haiyong Han, Morten Scheibye-Knudsen, Krzysztof Jozwiak, Coral Barbas, Irving W. Wainer

**Affiliations:** 1 Department of Biopharmacy, Medical University of Lublin, 20-093 Lublin, Poland; 2 Centre for Metabolomics and Bioanalysis (CEMBIO), Department of Chemistry and Biochemistry, Facultad de Farmacia, Universidad San Pablo-CEU, CEU Universities, Madrid, Spain; 3 Department of Biopharmaceutics and Pharmacodynamics, Faculty of Pharmacy, Medical University of Gdańsk, 80-416 Gdańsk, Poland; 4 Translational Gerontology Branch, National Institute on Aging, National Institutes of Health, Baltimore, MD 21224 USA; 5 Center for Healthy Aging, Department of Cellular and Molecular Medicine, University of Copenhagen, Copenhagen, Denmark; 6 Nutritional Interventions Group, Precision Nutrition and Aging, Institute IMDEA Food, Crta. de Canto Blanco no 8, 28049 Madrid, Spain; 7 Laboratory of Genetics and Genomics, National Institute on Aging, National Institutes of Health, Baltimore, MD 21224 USA; 8 Molecular Medicine Division, Translational Genomics Research Institute, Phoenix, AZ 85004 USA; 9 Laboratory of Clinical Investigation, National Institute on Aging, National Institutes of Health, Baltimore, MD 21224 USA; 10 PAZ Pharma, Mullica Hill, NJ 08062 USA

**Keywords:** Metabolomics, Cancer metabolism, Pancreatic cancer, Receptor pharmacology

## Abstract

Metabolic reprogramming contributes to oncogenesis, tumor growth, and treatment resistance in pancreatic ductal adenocarcinoma (PDAC). Here we report the effects of (*R*,*S*′)-4′-methoxy-1-naphthylfenoterol (MNF), a GPR55 antagonist and biased β_2_-adrenergic receptor (β_2_-AR) agonist on cellular signaling implicated in proliferation and metabolism in PDAC cells. The relative contribution of GPR55 and β_2_-AR in (*R*,*S*′)-MNF signaling was explored further in PANC-1 cells. Moreover, the effect of (*R*,*S*′)-MNF on tumor growth was determined in a PANC-1 mouse xenograft model. PANC-1 cells treated with (*R,S*′)-MNF showed marked attenuation in GPR55 signal transduction and function combined with increased β_2_-AR/Gα_s_/adenylyl cyclase/PKA signaling, both of which contributing to lower MEK/ERK, PI3K/AKT and YAP/TAZ signaling. (*R,S*′)-MNF administration significantly reduced PANC-1 tumor growth and circulating l-lactate concentrations. Global metabolic profiling of (*R,S*′)-MNF-treated tumor tissues revealed decreased glycolytic metabolism, with a shift towards normoxic processes, attenuated glutamate metabolism, and increased levels of ophthalmic acid and its precursor, 2-aminobutyric acid, indicative of elevated oxidative stress. Transcriptomics and immunoblot analyses indicated the downregulation of gene and protein expression of HIF-1α and c-Myc, key initiators of metabolic reprogramming in PDAC. (*R*,*S*′)-MNF treatment decreased HIF-1α and c-Myc expression, attenuated glycolysis, shifted fatty acid metabolism towards β-oxidation, and suppressed de novo pyrimidine biosynthesis in PANC-1 tumors. The results indicate a potential benefit of combined GPR55 antagonism and biased β_2_-AR agonism in PDAC therapy associated with the deprogramming of altered cellular metabolism.

## Introduction

Pancreatic ductal adenocarcinoma (PDAC) is the third leading cause of cancer death in the U.S. and, while there are multiple therapeutic approaches, only 9% of PDAC patients will survive beyond 5 years post diagnosis^1–3^. The poor prognosis for PDAC patients is due, in part, to metabolic reprogramming, which contributes to PDAC oncogenesis, tumor growth and treatment resistance^4,5^. Metabolic reprogramming is a multifaceted process involving alterations in glucose metabolism with a shift towards glycolysis, changes in lipid metabolism, and dysregulation in glutaminolysis and autophagy^6^. The central role of metabolic reprogramming has prompted the investigation of PDAC metabolism as a target in treatment protocols^4,5^.

Here, we report the effect on metabolic reprogramming produced by the administration of (*R*,*S*′)-4′-methoxy-1-naphthylfenoterol (MNF) in a murine PDAC xenotransplant tumor model derived using the PANC-1 human-derived PDAC cell line. The study stems from the observation that (*R,R*′)-MNF, a (*R,S*′)-MNF stereoisomer, attenuated glycolysis in the same PANC-1 xenotransplant model^7^. In the previous study, treatment with (*R,R*′)-MNF reduced the expression of glycolysis-associated proteins in tumor tissues and decreased l-lactate plasma concentrations relative to the data obtained with vehicle-treated animals. Incubation of PANC-1 cells in culture with (*R,R*′)-MNF led to reduction in glycolysis and attenuated expression of glycolytic enzymes relative to vehicle-treated cells.

(*R,R*′)-MNF is an antagonist of the G protein-coupled receptor, an atypical cannabinoid receptor whose primary endogenous ligand is L-α-lysophosphatidyl inositol (LPI)^8,9^. GPR55 couples to Gα_12_ and Gα_q_ proteins^10,11^ resulting in activation of pro-oncogenic PI3K/AKT, Ras/Raf/MEK/ERK, and Wnt/β-catenin signaling pathways^10,12,13^. GPR55 overexpression and activation are associated with increased proliferation and aggressiveness of PDAC cell lines and tumors. GPR55 expression negatively correlates with survival in patients with glioblastoma, breast cancer, and PDAC^10,14,15^, and genetic ablation of *Gpr55* significantly prolonged survival in the KPC transgenic mouse model of PDAC^15^. Thus, targeted GPR55 inhibition is a promising therapeutic strategy in PDAC.

This hypothesis is supported by the effects of GPR55 antagonists on PDAC cell lines. Incubation of PANC-1 cells with (*R,R*′)-MNF or the characterized GPR55 inhibitor CID16020046 attenuated EGFR-MEK-ERK and PI3K/AKT signaling, reduced the expression of EGFR, cyclin D1, β-catenin, pyruvate kinase M2 (PKM2), and multidrug resistance (MDR) transporters and decreased nuclear accumulation of HIF-1α and the phospho-active forms of PKM2 and β-catenin^7,16,17^. CID 16022046 and the GPR55 inhibitor cannabidiol (CBD) inhibited anchorage-dependent growth in ASPC1, HPAFII, BXPC3 and PANC-1 PDAC cell lines^15^. The functional consequences of GPR55 inhibition include cell cycle arrest and reduced cellular growth^7,15–17^, decreased glycolytic activity^7^, and increased cellular accumulation of MDR-exported cytotoxic drugs such as doxorubicin and gemcitabine^17^.

While the in vitro effects are promising, GPR55 inhibition does not directly translate to single agent efficacy in PDAC tumor models. The administration of (*R*,*R*′)-MNF in a murine PANC-1 xenotransplant model had no significant effect on tumor growth^7^ and treatment of KPC mice with CBD did not produce increased survival relative to vehicle-treated animals^15^. Nevertheless, it was determined that (*R*,*R*′)-MNF administration had a measurable effect on the PANC-1 tumor by reducing the expression of PKM2, EGFR, β-catenin, P-glycoprotein, and the l-lactate transporter^7^. In KPC mice, coadministration of CBD and gemcitabine results in a three-fold increase in survival relative to vehicle or single agent treatment^15^.

**Figure 1:**
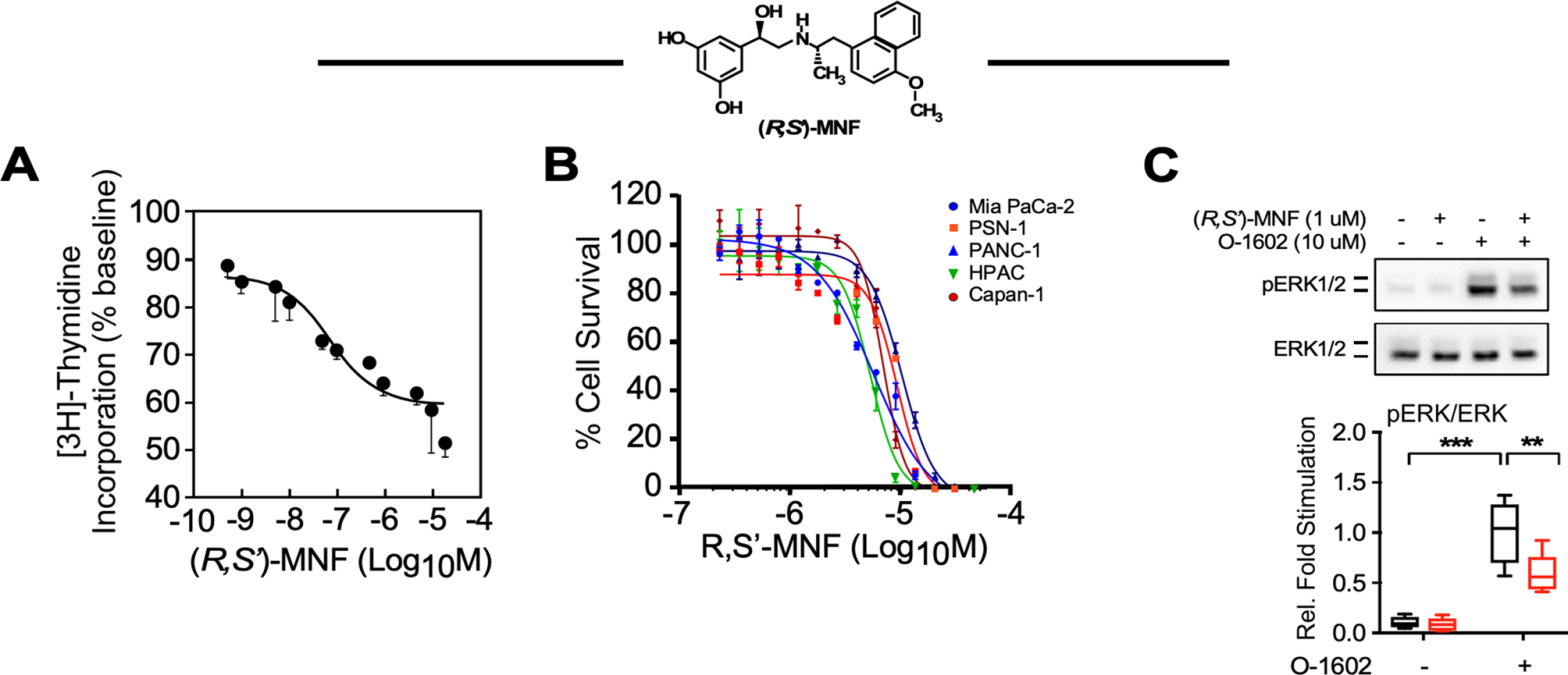
(*R*,*S*′)-MNF inhibits proliferation of PDAC cell lines. (**A**) (*R*,*S*′)-MNF (structure depicted) treatment reduces PANC-1 cell proliferation. [^3^H]-Thymidine incorporation into PANC-1 cells was assessed after incubation with increasing concentrations of (*R*,*S*′)-MNF for 24 h. Data are expressed as mean ± SD from 3 independent experiments. The calculated IC_50_ value was 0.11 ± 0.08 µM. (B) PDAC cell lines were exposed to (*R*,*S*′)-MNF for 72 h. Next, the viability of the cells was assessed using the Sulforhodamine B (SRB) assay. (*R*,*S*′)-MNF inhibited the survival of the MIA PaCa-2, PSN-1, PANC-1, HPAC, and Capan-1 cells with the IC_50_ of 5.2, 8.3, 9.6, 5.0, and 6.8 µM, respectively. (C) *Upper panel*, Immunoblot showing levels of phosphoactive and total forms of ERK after a 20-min pretreatment of serum-depleted PANC-1 cells with or without 1 µM (*R*,*S*′)-MNF followed by short term incubation with the GPR55 agonist O-1602 (10 µM, 20 min). *Lower panel*, Protein bands were quantified by densitometry, and the ratio of phosphorylated/total forms of ERK1/2 was calculated and plotted relative to O-1602-treated cells. Values are represented as boxplots with n = 6. Data analysis: one-way ANOVA followed by Tukey’s post-hoc test; **, ***P < 0.01, 0.001 vs. control or vs. marked treatments. All graphs were generated with GraphPad Prism v.8.4.3 (GraphPad Software, Inc., La Jolla, CA).

In addition to its inhibitory effect at GPR55, (*R*,*R*′)-MNF is a selective agonist of the β_2_-adrenergic receptor (β_2_-AR). Previous studies demonstrated that (*R*,*R*′)-MNF significantly reduced the growth of C6 glioblastoma xenograft tumors^18^ and that this effect reflects a synergism between the antagonistic properties of (*R*,*R*′)-MNF for GPR55 and its agonistic function for β_2_-AR^19^. In addition, the anti-tumor effects of (*R*,*R*′)-MNF in 1321N1 and melanoma cell lines appear to be predominately associated with the compound’s activation of the β_2_-AR^20,21^. The β_2_-AR agonist properties of (*R*,*R*′)-MNF would initially appear to be counterintuitive for the treatment of PDAC as catecholamine activation of β_2_-ARs increases tumor growth in KPC mice and PDAC xenograft models^22,23^ and promotes MEK/ERK signaling in PANC-1, BxPC-3 and MIA PaCa-2 PDAC cells^22^. However, catecholamines are a family of β_2_-AR agonists with limited steric bulk at the amino end of the molecule. This is unlike the MNF family that has a 4′-methoxy-1-naphthyl moiety at this position, which introduces steric bulk, additional interactive sites, and a second chiral center (Fig. 1). The structural differences between the two families are important as agonist binding to the β_2_-AR produces multiple receptor conformations whose relative distribution is a function of the agonist’s molecular structure^24–26^. Such structure-related differences in coupling to Gα_i_ and Gα_s_ proteins and G protein-independent signaling via β-arrestin-2 confer functional selectivity or biased agonism^24–26^.

Earlier in vitro studies indicated that both (*R*,*R*′)-MNF and (*R*,*S*′)-MNF reduce proliferation of 1321N1 astrocytoma, U118MG glioblastoma, and HepG2 hepatocellular carcinoma cells^20,27^ suggesting that both compounds target GPR55 and β_2_-AR. However, the effect of (*R*,*S*′)-MNF on GPR55 activity has not been definitively established and was one of the objectives of this study. A second objective was to probe the effect of biased agonism at the β_2_-AR on the growth and intracellular signaling in PACN-1 cells and derived tumors. This aspect of the investigation was based upon previous studies, which demonstrated that (*R*,*S*′)-MNF is a biased β_2_-AR agonist that selectively signals through Gα_s_ and does not recruit β-arrestin-2, while (*R*,*R*′)-MNF prefers Gα_s_ protein-coupling and is a weak partial agonist with respect to β-arrestin-2 recruitment^26^.

The overall goal of the present study was to examine the potential use of (*R*,*S*′)-MNF in the treatment of PDAC. This was accomplished by the determination of the effects of (*R*,*S*′)-MNF on expression and activation of key signaling proteins in PDAC cell lines using a multi-modal approach. The effect of (*R*,*S*′)-MNF on tumor growth was determined in a mouse PANC-1 xenotransplant model, and tumor tissues from treated and control animals were subjected to multiplatform, untargeted metabolomics and global microarray analyses in order to identify treatment-related metabolic signatures and signaling pathways. The results provide important clues about the roles that GPR55 antagonism and biased β_2_-AR agonism play in the treatment of PDAC, their effect on metabolic reprogramming and the therapeutic potential of (*R*,*S*′)-MNF in PDAC therapy.

## Materials and methods

### Materials

(*R*,*R*′)-Fenoterol [(*R*,*R*′)-Fen], (*R*,*S*′)-4′-methoxy-1-naphthylfenoterol [(*R*,*S*′)-MNF], and (*R*,*R*′)-MNF were synthesized as described previously^28^. The following compounds from Tocris Bioscience (Ellisville, MO, USA) were used for cell treatments: O-1602 (GPR55 agonist), Tocrifluor T1117 (T1117, fluorescent GPR55 agonist), myristoylated protein kinase inhibitor-(14–22)-amide (PKI), and forskolin (adenylyl cyclase activator). Salmeterol (selective β_2_-AR agonist) and ICI-118,551 (ICI, selective β_2_-AR inverse agonist) were purchased from Sigma-Aldrich (St-Louis, MO, USA).

### Cell culture

The human pancreatic cancer derived cell lines PANC-1 (RRID:CVCL_0480), MiaPaCa-2 (RRID:CVCL_0428), PSN-1 (RRID:CVCL_1644), HPAC (RRID:CVCL_3517), and Capan-1 (RRID:CVCL_0237) were obtained from American Tissue Culture Collection (Manassas, VA, USA) and were expanded for a few passages to enable the generation of new frozen stocks. Cells were resuscitated as needed and used for fewer than 6 months after resuscitation (no more than 12 passages). ATCC performs thorough cell line authentication utilizing Short Tandem Repeat profiling. Cell counting was done using Fuchs-Rosenthal hemacytometer.

PANC-1 cells were maintained in Dulbecco’s Modified Eagle’s Medium (DMEM) supplemented with 10% fetal bovine serum (FBS), 2 mM l-glutamine, 100 U/mL penicillin, and 0.1 mg/mL streptomycin. All other pancreatic cancer cell lines were cultured in RPMI-1640 containing 10% FBS, 100 U/mL penicillin, and 0.1 mg/mL streptomycin. HEK293 cells engineered to stably express the HA-tagged human GPR55 (hGPR55-HEK293) were a generous gift of Maria Waldhoer (Medical University of Graz, Graz, Austria)^29^. The hGPR55-HEK293 cells were cultured in DMEM with 4.5 g/L-glucose supplemented with 10% FBS, 0.2 mg/mL geneticin, 100 U/mL penicillin, and 0.1 mg/mL streptomycin. All culture media and supplements were from Thermo Fisher Scientific (Waltham, MA, USA).

Cells were maintained in a controlled environment (37 °C under humidified 5% CO2 in air), and the medium was replaced every 2–3 days. Prior to in vitro experiments, cells were seeded on 100 × 20 mm tissue culture plates and grown to ~ 70% confluency unless stated otherwise. At the time of the experiments, cells were between passages 8–11.

### [^3^H]-Thymidine incorporation assay in PANC-1 cells

PANC-1 cells were seeded in 24-well plates at 2.5 × 10^4^ cells per well and grown for 24 h at 37 °C. The medium was replaced and cells were incubated for 24 h with fresh media containing vehicle (0.1% DMSO) or (*R*,*S*′)-MNF (0–10 µM). [^3^H]-Thymidine (10 Ci/mmol; PerkinElmer Life and Analytical Sciences, Waltham, MA, USA) was added at 1 µCi per well and the incubation continued for 16 h. [^3^H]-Thymidine incorporation into DNA was measured as previously described^27^. Three independent experiments were conducted on three separate days.

### Cell survival assay

On Day 1, pancreatic cancer cells were seeded onto a 96-well plate (2000 cells/well) and cultured overnight. (*R*,*S*′)-MNF was added on Day 2 in a serial dilution starting at 25 µM. DMSO (0.1%) was used as vehicle control. The cells were incubated with the drug or vehicle for 72 h and then cell numbers were assessed using the Sulforhodamine B (SRB) assay (ab235935; Abcam, Cambridge, MA, USA). The percentage cell survival was calculated by dividing the blank-subtracted signal of drug-treated samples by blank-subtracted signal of vehicle control samples.

### Phosphorylation of eEF2, AKT, and ERK

PANC-1 cells were seeded in 6-well plates at 0.3 × 10^6^ cells per well and grown for 48 h at 37 °C. The medium was replaced with serum-free medium (SFM) and cells were starved for 3 h. Then the cells were subjected to one of the following challenges: (a) pretreatment with (*R*,*S*′)-MNF (1 µM) followed by treatment with O-1602 (10 µM); (b) treatment with either (*R*,*R*′)-MNF or (*R*,*S*′)-MNF (each at 5, 10 or 20 µM) for 20 min; (c) pretreatment with ICI (50 nM) or PKI (10 µM) followed by treatment with either (*R*,*R*′)-MNF or (*R*,*S*′)-MNF (each at 1 µM); (d) treatment with concentration gradient (1, 3.16, 10, 31.6, 100 µM) of (*R*,*R*′)-Fen or salmeterol; (e) treatment with concentration gradient (1, 10, 100, 1000, 10,000 nM) of forskolin. Each pretreatment and treatment lasted 20 min. Vehicle control (DMSO, 0.1%) was always used in parallel to the compound treatment. At the conclusion of the study, the cells were washed with ice-cold PBS, lysed, and then processed for immunoblot analysis.

### Cellular uptake and accumulation of T1117

The assay was performed as described previously^16^. In brief, hGPR55-HEK293 cells were plated at 1 × 10^4^ cells/well onto poly-D-lysine-coated 96-well black plates with clear bottom (BD Biosciences, San Jose, CA, USA) and allowed to attach overnight. Cell nuclei were stained with 1 µg/mL of Hoechst 33342 (ThermoFisher Scientific) for 30 min, washed with SFM, followed by the exposure to T1117 (15.6, 31.3, 62.5, 125, 250, 500 nM) for an additional 15, 30, 45 or 60 min. Alternatively, Hoechst-stained cells were pre-treated with fenoterol derivatives (1 µM or concentration gradient) for 30 min and loaded with 100 nM T1117 for another 30 min. Then, live cells were imaged using BD Pathway 855 BioImager workstation equipped with 20 × NA 0.75 dry objective (Olympus), environmental control (temperature: 37 °C, atmosphere 5% CO_2_:95% air), Photofluor light sources (Chroma Technology, Williston, VT, USA), and ORCA-AG Deep Cooled Digital Camera (Hamamatsu Photonics, Hamamatsu, Japan), all controlled by AttoVision v1.7 software (BD Biosciences). T1117 incorporation was assessed based on intensity of T1117-derived fluorescence within ring-shaped regions of interest (ROIs) established around Hoechst-stained nuclei. Numerical data were generated with BD Image Data Explorer and plotted using Prism v.8.4.3 software package (GraphPad Software, Inc., La Jolla, CA, USA; graphpad.com).

### PANC-1 tumor xenografts in mice

The studies were conducted using previously described model and validated protocol for the efficacy of agents in cancer chemotherapy^7,19^. Female Balb/c nude mice (aged between 6 and 8 weeks, weight 18–20 g) were purchased from HFK Bioscience Co., Ltd. (Beijing, China) and maintained under pathogen-free conditions with a 12 h light/12 h dark cycle in a ventilated cage (IVC) system with 5 animals per cage. Animals had free access to drinking water and were fed ad libitum with laboratory chow. Each mouse was inoculated subcutaneously at the right flank region with PANC-1 cells (5 × 10^6^) in 0.1 mL of PBS for tumor development. All animals were weighed and the tumor volumes measured in two dimensions using a caliper, and the volume expressed in mm^3^ using the formula: V = 0.5 *a* × *b*^2^, where *a* and *b* are the long and short diameters of the tumor, respectively. The treatments were started at Day 8 when the mean tumor size reached 139 ± 34 mm^3^ and the mice weighed 21.1 ± 1.1 g. The mice were assigned into groups (n = 10) using randomized block design based on their tumor volumes. The mice received a single intraperitoneal (ip) injection 5 days per week for 3 treatment cycles of either vehicle (1% hydroxypropyl-β-cyclodextrin) (Control), 20 mg kg^−1^ (Arm 1) or 40 mg kg^−1^ (*R*,*S*′)-MNF (Arm 2). The dosing volume was adjusted according to weight (10 µL/g). Tumor volumes and weights were determined at the beginning and end of each dosing cycle, while visual estimation of food and water consumption, eye/hair matting, and behavior such as mobility were determined daily. In Control and Arm 2, 5 days after the end of the last dosing cycle (Day 33) the animals were weighed and then euthanized by cervical extension, while in Arm 1, five of the animals were euthanized one day after the last administration of (*R*,*S*′)-MNF (Day 27) and the remaining 5 animals were euthanized five days after the end of the last dosing cycle (Day 33). Plasma samples were collected one day after the last day of dosing (Day 27) and when all the animals were euthanized at the conclusion of the studies (Day 33). The tumor volumes of all the animals in the study did not exceed 3000 mm^3^ and, therefore, no animals were euthanized before the end of their particular arm of the study. After collection of plasma samples, all the tumors were excised, weighed, divided into three portions and snap frozen. Only the Control and Arm 2 tumors were used to analyze changes in the metabolome, transcriptome, and cancer biomarker expression by immunoblotting.

#### Determination of (*R*,*S*′)-MNF concentrations in plasma and tumor tissues

The concentrations of (*R*,*S*′)-MNF in plasma and PANC-1 tumor samples obtained from Arm 1 and Arm 2 were analyzed by LC–MS using a previously described method^18^.

#### Determination of l-lactate plasma concentrations

Pre- and post-treatment plasma samples were obtained from all three arms of the study and were analyzed for l-lactate concentrations using commercially available kit (Abcam, ab65331). The assays were conducted following the manufacturer’s instructions.

#### Sample preparation and multiplatform metabolomics study

The method for preparation of PANC-1 xenograft tumor samples was adapted from Gonzalez-Pena and colleagues^30^. Metabolomics analyses was performed according to established procedures (see Supporting Information for full methodological details). MetaboAnalyst v5.0 (metaboanalyst.ca) data annotation tool was used for testing the relationships between variables^31^, while identification of compounds with accurate mass was searched for possible ID against available databases using online advanced CEU Mass Mediator (CMM) tool^32^. Compound identification by GC–MS was performed using multiple libraries including the NIST 14 Mass Spectral Library^33^.

### Gene expression microarray methodology

Total RNA was extracted from tumor lysates (n = 10 per group) using Trizol reagent (Thermo Fisher Scientific) and further purified using RNeasy mini columns (Qiagen, Valencia, CA, USA). RNA quantity and quality were determined using the Agilent Bioanalyzer RNA 6000 Chip (Agilent Technologies, Santa Clara, CA, USA). RNA was labeled and hybridized to the Illumina Human HT-12 v4 BeadChip using standard Illumina protocols. Following post-hybridization rinses, arrays were incubated with streptavidin conjugated Cy3, and scanned at a resolution of 0.53 µm using an Illumina iScan scanner. Hybridization intensity data were extracted from the scanned images using Illumina BeadStudio GenomeStudio software, v2011.1 (illumina.com). Raw data were subjected to Z-normalization. Significant genes were selected by the z-test < 0.05, false discovery rate < 0.30, as well as z-ratio > 1.5 in both directions and ANOVA *P*-value ≤ 0.05^34^. The raw data file and the filtered, normalized results are available online in the Gene Expression Omnibus, Accession Number GSE113077. Additional information can be found in the Supporting Information.

### Gel electrophoresis and immunoblotting

Lysates from PANC-1 cells and excised tumor xenografts were prepared and protein expression levels were measured by standard western blot procedures^7^. The primary antibodies used were as follows: CYR61 (HPA029853; RRID:AB_10611822; Sigma-Aldrich); and β-catenin (sc-7199; RRID:AB_634603; Santa Cruz Biotechnology, Dallas, TX, USA); HIF-1α (ab179483; RRID:AB_2732807), and β-actin (ab6276; RRID:AB_2223210; Abcam); cyclin D1 (ms-210-p1; RRID:AB_62132) and α-tubulin (MA1-80017; RRID:AB_2210201; Thermo Fisher Scientific); PKM2 (4053; RRID:AB_1904096), LDHA (3582; RRID:AB_2066887), c-Myc (9402; RRID:AB_2151827), YAP/TAZ (8418; RRID:AB_10950494), LATS1/2 (3477; RRID:AB_2133513), phospho-eEF2 (Thr56, 2331; RRID:AB_10015204), eEF2 (2332; RRID:AB_10693546), phospho-AKT (Ser473, 4060; RRID:AB_2315049), AKT (4691; RRID:AB_915783), phospho-ERK1/2 (Thr202/Tyr2104, 4376; RRID:AB_331772), ERK1/2 (4695; RRID:AB_390779), and phospho-PKA substrates (9624; RRID:AB_331817; Cell Signaling Technology, Danvers, MA, USA) and used at dilutions recommended by the manufacturers. Signals were detected by enhanced chemiluminescence using ECL Plus (GE Healthcare, Piscataway, NJ, USA) or SignalFire Plus ECL Reagent (Cell Signaling Technology) and images were acquired using ChemiDoc Imager (Bio-Rad) or Azure c400 (Azure Biosystems, Dublin, CA, USA) until signal saturation. The optical density of each band was quantified on sub-saturated images using ImageJ software (National Institutes of Health, Bethesda, MD, USA). All expression values were normalized to total protein load (Ponceau S-stained membrane) and then to β-actin, α-tubulin, or respective total proteins as indicated.

### Statistical analysis

Sigmoidal dose–response curves (IC_50_ curves) were determined using the ‘nonlinear regression (curve fit)’ model contained within the Prism v8.4.3. For tumor volume and animal weight analyses, statistical comparisons between treated and control groups were performed using unpaired Student’s t-test. For immunoblot analyses, data normality and variance were verified by the Shapiro–Wilk test and the *F* test, respectively. Differences between control and (*R*,*S*′)-MNF groups were evaluated using either unpaired t test for samples with normal distribution and equal variances or nonparametric (Mann–Whitney) test. *P*-values < 0.05 were considered significant.

### Ethical approval

The xenograft studies were conducted at the Crown Biosciences, Ltd. facilities and all protocols were approved by the Animal Care and Use Committee at CrownBio (AN-1407-009-164), which are based on “the Guide for the Care and Use of Laboratory Animals”^35^. The study was carried out in compliance with the ARRIVE guidelines.

## Results

### (*R*,*S*′)-MNF attenuates pro-oncogenic signaling and cellular proliferation in PANC-1 cells

To determine the effect of (*R*,*S*′)-MNF on cancer cell proliferation we treated PANC-1 cells with the drug for 24 h and observed a concentration-dependent reduction in [^3^H]-thymidine incorporation with a calculated IC_50_ value of 110 ± 80 nM (Fig. 1A). This antimitogenic action of (*R*,*S*′)-MNF was reproduced in a cell survival study with a panel of human-derived PDAC cell lines including PANC-1, MIA PaCa-2, PSN-1, HPAC, and Capan-1 cells. (*R*,*S*′)-MNF exposure of the cell lines led to complete attenuation of cell viability with low micromolar IC_50_ values (Fig. 1B).

**Figure 2:**
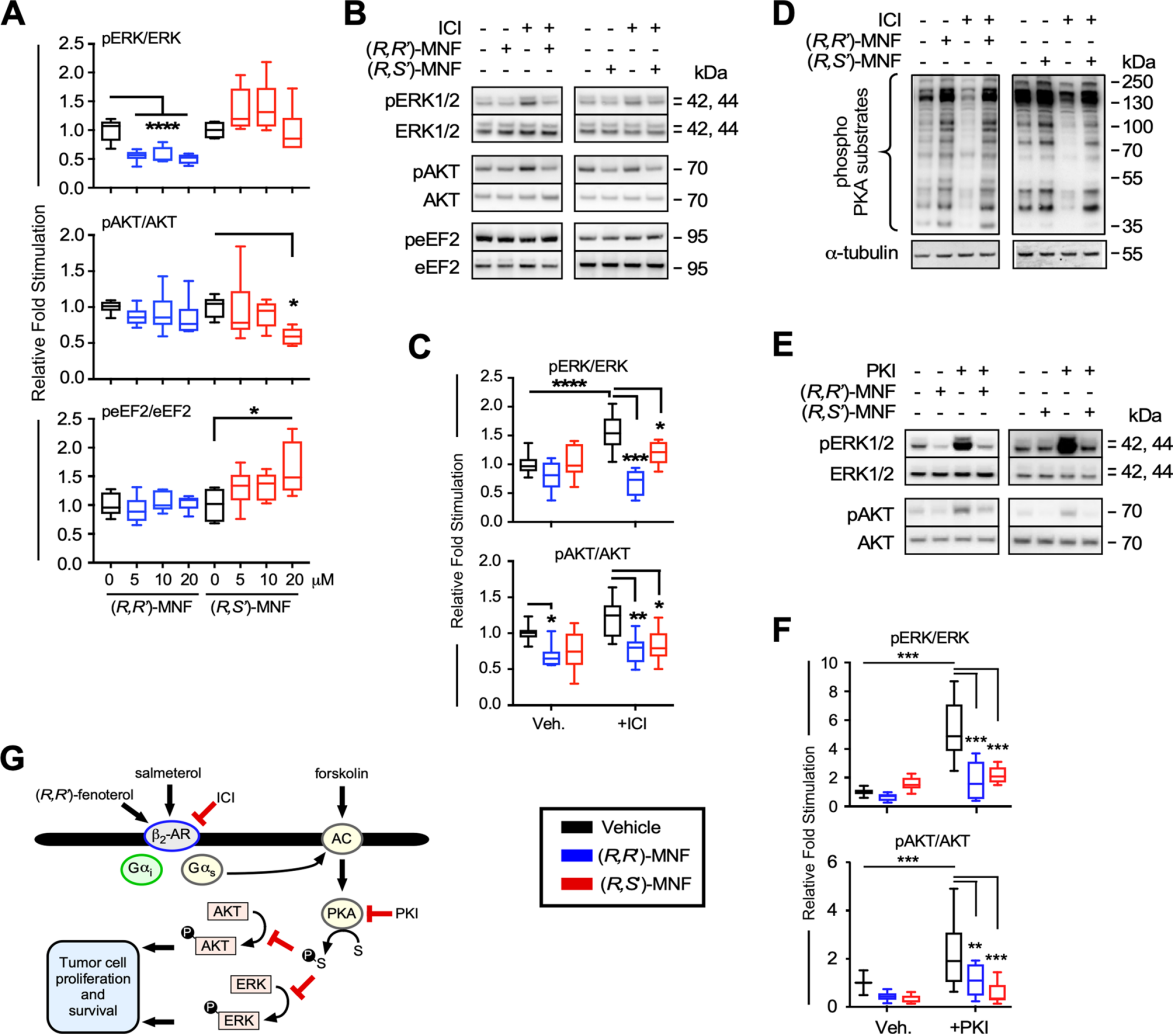
Biological activity of (*R*,*S*′)-MNF in PANC-1 carcinoma cell line. (**A**) PANC-1 cells were treated with 5, 10, 20 µM (*R*,*R*′)-MNF (blue), (*R*,*S*′)-MNF (red) or with vehicle (0.1% DMSO; black) for 20 min and lysed. Level of phosphorylated and total ERK, AKT and eEF2 was assessed by immunoblotting in the obtained lysates. Values are represented as boxplots with n = 6. (**B**) Immunoblots showing levels of phosphorylated and total forms of ERK, AKT and eEF2 after a 20-min pretreatment of serum-depleted PANC-1 cells with or without 50 nM ICI followed by incubation either with (*R*,*R*′)-MNF or (*R*,*S*′)-MNF (1 µM, 20 min). (**C**) Protein bands were quantified by densitometry, and the ratio of phosphorylated/total forms of the proteins was calculated and plotted relative to vehicle-treated cells. Values are represented as boxplots with n = 7. (**D**) PANC-1 cells were treated as on panel F and phosphorylated targets of PKA were visualized as a marker for PKA activation. (**E**) Effect of (*R*,*R*′)-MNF or (*R*,*S*′)-MNF on the PKI-dependent (10 µM) activation of ERK and AKT. Representative blots are depicted on panel (**F**). (**G**) β_2_-AR activation inhibits ERK and AKT phosphorylation. Illustration was created using Canvas X Draw v.7.0.2 for macOS (Canvas GFX, Boston, MA; canvasgfx.com). Data analysis: one-way ANOVA followed by Tukey’s post-hoc test; *, **, ***, **** *P* < 0.05, 0.01, 0.001, 0.0001 vs. control or vs. marked treatments. All box plots were generated with GraphPad Prism v.8.4.3 (GraphPad Software, Inc., La Jolla, CA).

Our previous work has established the approximate equipotency of (*R*,*R*′)-MNF and the GPR55 antagonist CID16020046 at lowering chemoresistance in a number of cancer cell types including PANC-1 cells^17^. Here, incubation of PANC-1 cells with 1 µM (*R*,*S*′)-MNF significantly decreased ERK phosphorylation stimulated by the GPR55 ligand O-1602 (Fig. 1C). Activated GPR55 undergoes time-dependent internalization, a process that can be followed using T1117 a fluorescent agonist of GPR55 (Supporting Information Fig. S1A). T1117 uptake experiments have been previously used to investigate ligand’s interactions with the GPR55^16^. To ascertain whether (*R*,*S*′)-MNF had a direct effect on ligand-induced GPR55 internalization, HEK293 cells stably expressing 3 × HA-tagged human GPR55 were preincubated with increasing concentrations of (*R*,*S*′)-MNF for 30 min followed by the addition of T1117 (Supporting Information Fig. S1B). (*R*,*S*′)-MNF elicited a dose-dependent reduction in T1117 incorporation, yielding IC_50_ of 1.3 µM. When (*R*,*S*′)-MNF was assessed in parallel with its isomer (*R*,*R*′)-MNF, which served as positive control, both compounds elicited a significant ~ 50% and ~ 70% reduction in T1117 incorporation, respectively (Supporting Information Fig. S1C). In contrast, the β_2_-AR agonist, (*R*,*R*′)-Fen, which has previously been used as a negative control for GPR55 antagonism^16^, did not reduce intracellular T1117 accumulation. Thus, it would appear that (*R*,*S*′)-MNF was an effective inhibitor of GPR55 internalization and signaling.

(*R*,*S*′)-MNF and (*R*,*R*′)-MNF display functionally selective pattern of signaling via β_2_-AR^26^, which could be reflected in the differential response of PANC-1 cells exposed to these stereoisomers (Fig. 2A-F). ERK phosphorylation was significantly attenuated by (*R*,*R*′)-MNF (5–20 µM) and not by (*R*,*S*′)-MNF. Activity towards AKT was quite the opposite; while (*R*,*R*′) isomer did not affect the levels of phosphoactive AKT, the (*R*,*S*′)-MNF diminished AKT phosphorylation at 20 µM dose (Fig. 2A). Moreover, increase in phosphorylated eEF2 was observed only in PANC-1 cells exposed to (*R*,*S*′)-MNF (20 µM), but not in response to equimolar concentration of (*R*,*R*′)-MNF (Fig. 2A). The contribution of β_2_-AR to PANC-1 signaling was further addressed using pharmacological approach. Previous studies have identified ICI-118551 (ICI) as a selective inverse agonist of β_2_-AR associated with the inhibition of Gα_s_/adenyl cyclase/PKA signaling and the promotion of β-arrestin-mediated ERK activation^36^. Here, incubation of PANC-1 cells with ICI alone induced a significant increase in ERK phosphorylation (Fig. 2B and C) accompanied by a drop in the level of phosphorylated PKA substrates (Fig. 2D). The fact that (*R*,*R*′) and (*R*,*S*′)-MNF (both at 1 µM) suppressed the observed effects of ICI is consistent with their role as β_2_-AR ligands. Moreover, addition of (*R*,*R*′)-MNF and (*R*,*S*′)-MNF (both at 1 µM) to PANC-1 cells pretreated with PKI, a selective inhibitor of PKA, blocked PKI-dependent elevation of phosphoactive forms of ERK and AKT (Fig. 2E and F), pointing towards PKA involvement in ERK and AKT activation (Fig. 2G). Incubation of PANC-1 cells with the Gα_s_-selective β_2_-AR agonist (*R*,*R*′)-Fen^37^ and the Gα_s_-biased partial β_2_-AR agonist salmeterol^38^ also dose-dependently reduced the levels of phospho-ERK and phospho-AKT; however, increased phospho-eEF2 levels were observed only in cells treated with salmeterol (Supporting Information Fig. S2A–D). Inhibitory action towards ERK and AKT elicited by the β_2_-AR agonists was mimicked by forskolin, an adenylyl cyclase activator (Supporting Information Fig. S2E and F). The data indicate that activation of β_2_-AR/ Gα_s_/adenylyl cyclase/PKA axis in PANC-1 cells contributes to the suppression of AKT and ERK phosphorylation (Fig. 2G).

### (*R*,*S*′)-MNF suppresses tumor growth in the PANC-1 mouse xenograft model

**Figure 3:**
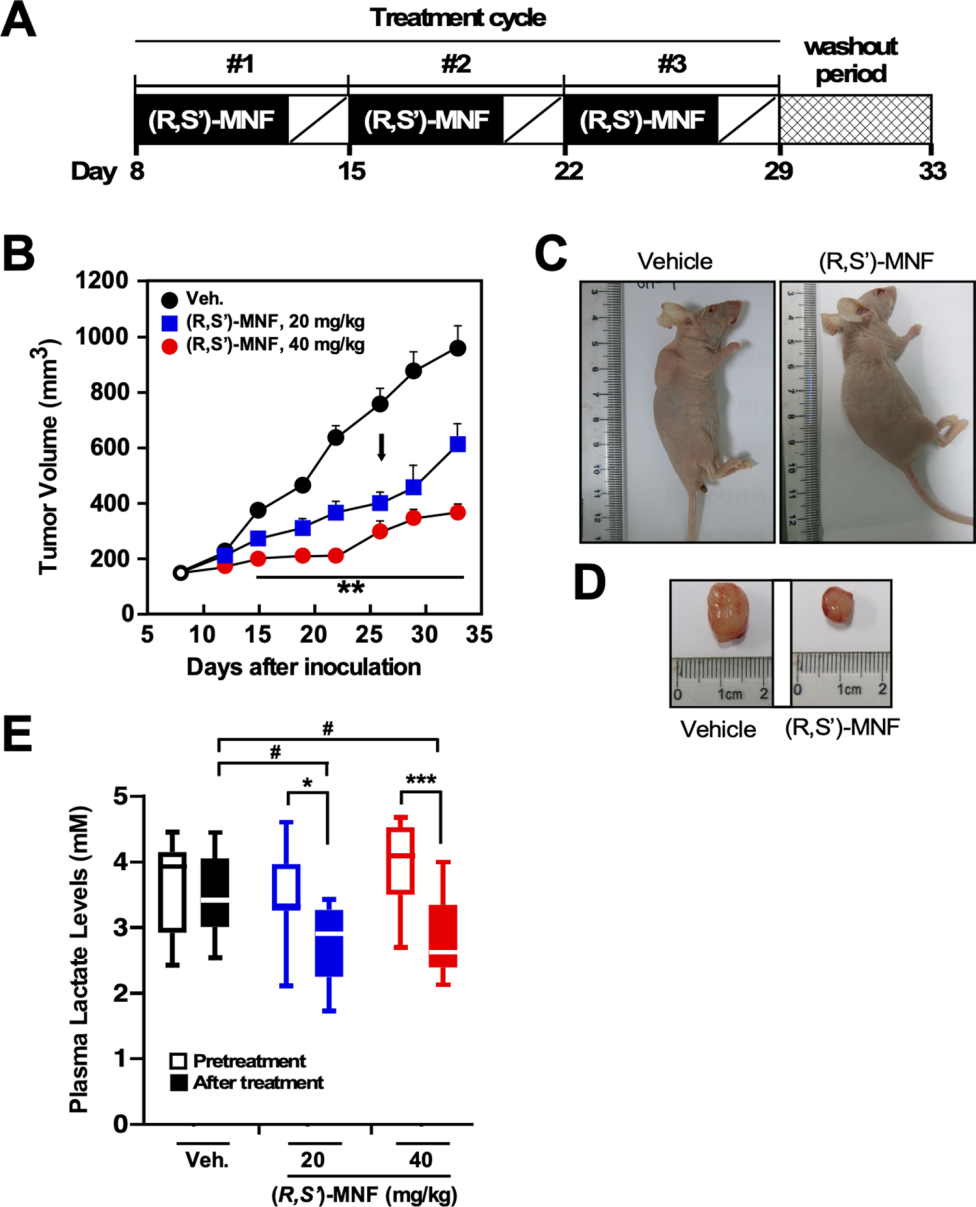
(*R*,*S*′)-MNF treatment reduces PANC-1 tumor growth in a mouse xenograft model. (**A**) Protocol design. (**B**) Tumor volume was determined after i.p. administration of vehicle (1% hydroxypropyl-β-cyclodextrin), 20 mg kg^−1^ (Arm 1) or 40 mg kg^−1^ (*R*,*S*′)-MNF (Arm 2) once daily for 5 days per week for 3 weeks (n = 10 per group). The black arrow depicts the last day of (*R*,*S*′)-MNF administration. Data represent mean + SD. **P < 0.01 vs. control group of mice. Representative images of mice (**C**) and excised tumors (**D**) are shown. (**E**) Plasma lactate levels were measured on Day 8 (Pretreatment, open symbols) and at completion of the (*R*,*S*′)-MNF treatment (After treatment, filled symbols) (n = 8–10 per group). *P < 0.05, ***P < 0.001; #*P* < 0.05 vs. control group of mice at the completion of the study. The dose- and time-dependent trajectories as well as the box plot were generated with GraphPad Prism v.8.4.3 (GraphPad Software, Inc., La Jolla, CA).

A dose-ranging study was conducted to determine the effect of (*R*,*S*′)-MNF treatment on the growth of PANC-1 tumors maintained as xenografts in female nude mice (Fig. 3A). The intraperitoneal administration of 20 mg kg^−1^ (Arm 1) and 40 mg kg^−1^ of (*R*,*S*′)-MNF (Arm 2) led to significant dose-dependent reduction in tumor growth, assessed as decreased tumor volume as compared to tumor-bearing mice treated with vehicle (Fig. 3B). The average tumor volume in the vehicle-treated mice increased from 142 ± 8 on Day 8 to 752 ± 57 mm^3^ after the last dose (Day 26) and 957 ± 79 mm^3^ at the termination of the study (Day 33), representing a ∼ 700% increase over the course of the study (Supporting Information Table S1). The average tumor volume in Arm 1 and Arm 2 of the study passed from 143 ± 8 on Day 8 to 399 ± 41 mm^3^ and 205 ± 22 mm^3^, respectively, on Day 26 and 615 ± 73 mm^3^ and 259 ± 27 mm^3^, respectively, on Day 33, representing a ∼ 400% increase (Arm 1) and ∼ 250% increase (Arm 2) over the course of the study (Supporting Information Table S1). One week after the last course of treatment, the average tumor volume in (*R*,*S*′)-MNF-treated mice was significantly smaller than that of control group (*P* < 0.05), especially in mice that had received 40 mg kg^−1^ of (*R*,*S*′)-MNF (Fig. 3C). The (*R*,*S*′)-MNF-mediated growth suppression of PANC-1 tumor xenografts was not correlated with obvious difference in tumor neovascularization (Fig. 3D). No significant loss in body weight was observed in Control group and Arm 1 animals while in Arm 2, there was a small but significant ∼ 10% decrease in body weight, 21.3 ± 0.5 g (Day 8) to 19.2 ± 2.2 g (Day 33) (Supporting Information Table S1).

The plasma concentrations of (*R*,*S*′)-MNF were determined one day after the last dose of the drug (Day 26) and at termination of the Study (Day 33). After the last dose, significant amounts of (*R*,*S*′)-MNF were detected in the plasma samples with 3.49 ± 1.00 ng/mL (Arm 1) and 13.2 ± 6.50 ng/mL (Arm 2), respectively (n = 10 for both groups). By Day 33, (*R*,*S*′)-MNF was not detected in the plasma while significant amounts were detected in tumor tissues, 33.1 ± 10.0 ng/g (Arm 1) and 43.9 ± 32.7 ng/g of (*R*,*S*′)-MNF (Arm 2) (n = 5), indicating that (*R*,*S*′)-MNF accumulates within the PANC-1 tumor.

Because the high production and release of l-lactate by glycolytic cancer cells contribute to the extracellular acidification of the tumor microenvironment^39,40^, we speculated that the antiproliferative activity of (*R,S*′)-MNF could be associated with the decrease in circulating l-lactate levels in mice harboring PANC-1 tumor xenografts. Serum l-lactate was determined at pre-dosing (Day 8) and at Day 33, one week after the administration of the last dose of (*R,S*′)-MNF. On Day 8, l-lactate levels were similar among the three groups of mice (Control, 3.59 ± 0.71 mM; Arm 1, 3.47 ± 0.67 mM; Arm 2, 3. 29 ± 0.66 mM), but were significantly different between the treatment and control groups on Day 33 (Control, 3.46 ± 0.62 mM; Arm 1, 2.75 ± 0.62 mM (*P* < 0.05); Arm 2, 2.81 ± 0.60 mM (*P* < 0.001) (Fig. 3E).

### Multiplatform, non-targeted metabolomics signature of PANC-1 tumor xenografts

**Figure 4:**
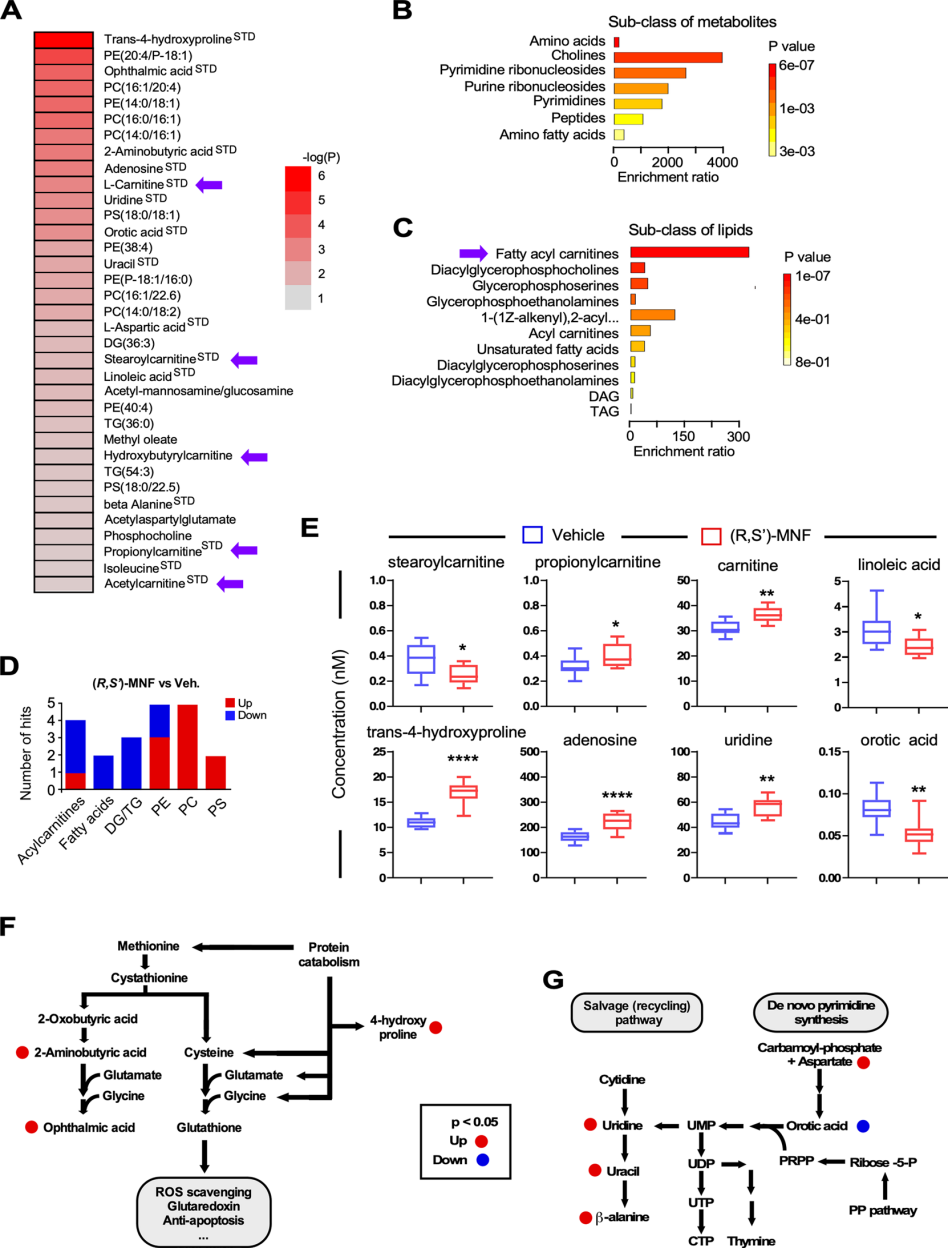
(*R*,*S*′)-MNF treatment elicits distinct metabolomics signature in PANC-1 tumor xenografts. (**A**) Heatmap depicting the *P*-values [− log10(*P*)] from the univariate analyses of the 35 metabolites shown to be significantly altered in response to (*R,S*′)-MNF. Purple arrows indicate l-carnitine and different species of acylcarnitines. The heatmap was created using Microsoft Excel v.16.56 for Mac (Redmond, WA). (**B**, **C**) The Over Representation Analysis (ORA) module of MetaboAnalyst 5.0 was used to provide better representation of the sub-classes of metabolites (**B**) and lipids (**C**) with their enrichment significance in our dataset of 35 significantly altered compounds. Significantly enriched ‘fatty acyl carnitines’ are indicated with purple arrow. (**D**) Numbers of biochemicals related to lipid metabolism that were differentially expressed in the PANC-1 tumor xenografts from (*R*,*S*′)-MNF vs. control groups; n = 9–10 mice per group. (**E**) Relative abundance of selected biochemicals between the two experimental groups, as quantified by targeted metabolomics. See Supporting Information Table S3 for complete list. *, **, ****P < 0.05, 0.01, 0.0001 vs. vehicle controls. The box plots were generated with GraphPad Prism v.8.4.3 (GraphPad Software, Inc., La Jolla, CA). (**F**) Altered cysteine/methionine metabolism in PANC-1 tumor xenografts through formation of ophthalmic acid upon (*R*,*S*′)-MNF treatment. (**G**) Schematic representation of the change in metabolites associated with pyrimidine synthetic and salvage pathways following (*R*,*S*′)-MNF treatment. Metabolic pathway schemes were drawn using Canvas X Draw v.7.0.2 for macOS (Canvas GFX, Boston, MA; canvasgfx.com). DG, DAG, diacylglycerol; TG, TAG, triacylglycerol; PE, phosphatidylethanolamine; PC, phosphatidylcholine; PS, phosphatidylserine.

A multiplatform, non-targeted metabolomics study was carried out to analyze the global metabolic profiles of PANC-1 tumor xenografts from Control and Arm 2 groups on Day 33 (n = 10 per group). The metabolomics data matrix consisted of 584 and 316 metabolic features found in ESI(+) and ESI(-) LC–MS ionization mode, respectively, and 514 features found in CE-MS analysis. In GC–MS analysis, 101 metabolites were identified using the target metabolite Fiehn GC–MS Metabolomics RTL library (G1676AA, Agilent), the in-house developed CEMBIO-library and the NIST mass spectra library. A total of 201 features in ESI(+), 94 in ESI(-) LC–MS mode, 93 in CE-MS, and 28 in GC–MS were significantly altered between the PANC-1 tumors collected one week after the last treatment cycle with (*R*,*S*′)-MNF and the Control group (Day 33). Once data were acquired, the raw chromatographic spectra were aligned and cleaned from background noise. The quality of the analysis was evaluated using non-supervised principal component analysis (PCA-X). A clustering of QCs was observed in each of the four data sets (Supporting Information Fig. S3A–D) indicating the precise analytical outcome^41^. Score plots from the supervised orthogonal partial least squares (OPLS-DA) discriminant analysis showed separation between the (*R*,*S*′)-MNF and Control groups (Supporting Information Fig. S3E–H) which suggests existing biochemical perturbation. Multivariate statistical approach based on the constructed OPLS-DA models, combining the p(corr) (the loadings scaled as correlation coefficient) in combination with Variable Influence on the Projection (VIP) was used to select metabolites that contribute to class separation. Multivariate along with univariate statistical analysis resulted in the 201 and 94 metabolic features significant in ESI(+) and ESI(-) LC–MS data; 93 and 28 features significant in CE-MS and GC–MS analysis, respectively. Finally, the identity of 74 metabolites that belong to 14 chemical classes have been confirmed by the analysis of the fragmentation pattern of the compounds and the comparison with the fragmentation pattern of chemical standards (^STD^), when available (Supporting Information Table S2). We graphically represented as heatmap the 35 metabolites that were shown by both multivariate and univariate analyses to be significantly altered in response to (*R*,*S*′)-MNF (Fig. 4A). Remaining 39 metabolites were identified based solely on the multivariate OPLS-DA models that are prone to over-fitting, and thus their significance needs to be interpreted with caution. The impact of (*R*,*S*′)-MNF on carnitine and various acylcarnitines (Fig. 4A, purple arrows) led us to use the Overrepresentation Analysis (ORA) module of MetaboAnalyst to further dissect our dataset of 35 metabolites based on their chemical classes (Fig. 4B and C). Significant enrichment of amino acids, cholines, and ribonucleosides along with fatty acyl carnitines was observed in tumors of (*R*,*S*′)-MNF-treated mice. Noteworthily, all 3 glycerolipids and 2 fatty acids were lower with (*R*,*S*′)-MNF treatment while 10 out of 12 glycerophospholipids were significantly increased in response to the treatment (Fig. 4D). Targeted metabolomics analysis confirmed the differential effect of (*R*,*S*′)-MNF on a number of metabolites, including stearoylcarnitine, propionylcarnitine, carnitine, linoleic acid, *trans*-4-hydroxyproline, adenosine, uridine, and orotic acid (Fig. 4E and Supplementary Table S3). The increase in carnitine, a key component of long-chain fatty acid transport into the mitochondria for β-oxidation, in contrast to the decrease in long-chain acylcarnitine species e.g., palmitoyl and stearoyl (Supporting Information Table S2) may suggest a downregulation of the carnitine shuttle. The reduction in 3-hydroxybutyric acid, a ketone body, and of citrate, a Krebs cycle intermediate and precursor for the synthesis of fatty acids and cholesterol, is worth noting.

Thirty biochemicals that showed significant differences between (*R*,*S*′)-MNF (Arm 2) and Control groups have unique accession number (HMDB and/or CAS) and International Chemical Identifier code (Supporting Information Table S4.

Concomitant with the change in lipid metabolism intermediates, there was a significant increase in the levels of ophthalmic acid (+ 237%) and its precursor, 2-aminobutyric acid (+ 84%), indicative of elevated cysteine/methionine metabolism and associated oxidative stress due to the ‘hijacking’ of the GSH-synthesizing pathway^42^ (Fig. 4F). With respect to pyrimidine nucleotide biosynthesis, aspartate accumulation was associated with a significant decrease in orotic acid, a precursor of uridine monophosphate, the biochemical that other pyrimidine nucleotides are derived from (Fig. 4G). Yet, two pyrimidine salvage pathway intermediates (e.g., uridine, uracil) and the uracil catabolite β-alanine were also all increased upon treatment with (*R*,*S*′)-MNF (Fig. 4G and Supporting Information Table S2). Lastly, *trans*-4-hydroxyproline was increased by 53% in (*R*,*S*′)-MNF-treated tumors, consistent with greater connective tissue degradation in response to elevated oxidative stress^43^. These results provide support for sustained metabolic deprogramming of PANC-1 tumor xenografts one week after (*R*,*S*′)-MNF withdrawal.

### Global gene expression signature in PANC-1 tumor xenografts

To unravel the molecular underpinings of the antitumor activity of (*R*,*S*′)-MNF, we performed a whole-genome microarray of RNA derived from PANC-1 tumor xenografts.

**Figure 5:**
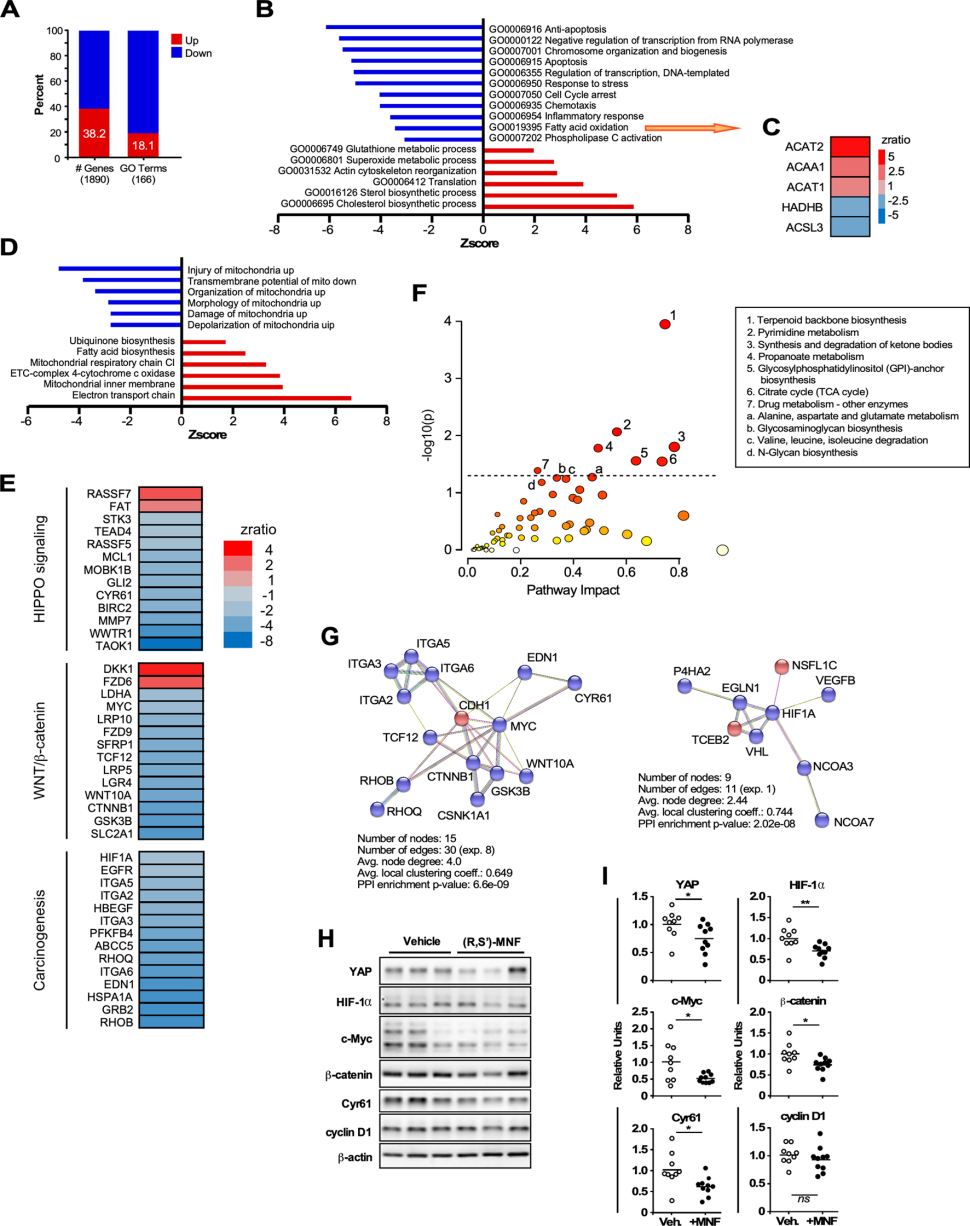
(*R*,*S*′)-MNF treatment elicits distinct gene expression signature in PANC-1 tumor xenografts. (**A**) Percent of genes and GO Terms that were up- and down-regulated in the (*R*,*S*′)-MNF:vehicle pairwise comparison. (**B**) Enrichment of top GO Terms that were up- (positive Z-scores) and down-regulated (negative Z-scores) after (*R*,*S*′)-MNF treatment. (**C**) Heatmap depicting the expression of genes implicated in fatty acid oxidation that were significantly impacted by (*R*,*S*′)-MNF. (**D**) Enrichment of top GO Terms related to Mitochondrial Pathway dataset from the (*R*,*S*′)-MNF:vehicle pairwise comparison. The complete list can be found in Supporting Information Table S5. (**E**) Heatmap showing the statistically significant Z-ratio changes in expression of selected genes in the (*R*,*S*′)-MNF:vehicle pairwise comparison. These genes are implicated in a handful of pro-oncogenic signaling pathways, including Hippo, Wnt-β-catenin, and EGF receptor. (**F**) Input for the multi-omics Joint Pathway Analysis (JPA) from MetaboAnalyst 5.0 consisted of the 74 metabolites gathered in Supporting Information Table S2 and the 1890 differentially expressed transcripts identified by microarray profiling. y axis, enrichment significance; x axis, pathway impact for network topology. Dotted line highlights pathways significantly impacted as defined by enrichment significance *P* < 0.05 [log(*P*) > 1.3]. (**G**) String protein–protein interaction database (v 11.5; string-db.org) was used to visualize the putative impact of (*R,S*′)-MNF on functional c-Myc (left panel) and HIF-1α (right panel) protein–protein association based on the transcriptional responses of PANC-1 tumors to (*R,S*′)-MNF treatment. Red nodes, significantly upregulated genes; blue nodes, significantly downregulated genes. In String network analysis, nodes represent proteins whereas edges represent functional protein–protein associations. If the number of edges for analyzed dataset is higher than expected for a random set of proteins (the ‘exp.’ value), the clustered proteins are biologically connected as a group. The average node degree indicates how many interactions a protein has on average in the network. The clustering coefficient is a measure of how connected the nodes in the network are. Protein–protein interaction (PPI) enrichment *P*-value is a measure of significance for biological association of investigated proteins. (**H**) Lysates prepared from PANC-1 tumor xenografts were separated by SDS-PAGE and immunoblotted using primary antibodies specific for YAP, HIF-1α, c-Myc, β-catenin, CYR61, cyclin D1, and β-actin (n = 9–10 mice per group). Representative blots are depicted. (**I**) Scatter plots depict densitometric quantitation of each protein band followed by normalization to β-actin levels. *, **P < 0.05, < 0.01 vs. control. The heatmaps were created using Microsoft Excel v.16.56 for Mac (Redmond, WA). The bar graphs and scatter plots were generated with GraphPad Prism v.8.4.3 (GraphPad Software, Inc., La Jolla, CA).

Of the 1890 differentially expressed genes in the (*R*,*S*′)-MNF:vehicle pairwise comparison, 38.2% were upregulated and 61.8% were downregulated (Fig. 5A). Parametric analysis of gene set enrichment (PAGE) identified 166 significantly altered GO Terms (biological process classification) in response to (*R*,*S*′)-MNF, of which only 18.1% were upregulated (Fig. 5A). Among the top GO Terms that were negatively impacted by (*R*,*S*′)-MNF treatment were those belonging to apoptosis, cell cycle arrest, inflammation, and fatty acid oxidation while pathways related to superoxide metabolic process, translation, and sterol/cholesterol biosynthesis were among the top upregulated GO Terms (Fig. 5B and Supporting Information Table S5). A heatmap of the genes associated with the significant alteration in fatty acid oxidation is depicted in Fig. 5C. These genes encode for acetyl-CoA acetyltransferase 1 and 2 (*ACAT1*, *ACAT2*), acetyl-CoA acyltransferase 1 (*ACAA1*), mitochondrial trifunctional enzyme that catalyzes the last three of the four reactions of the mitochondrial beta-oxidation pathway (*HADHB*), and a fatty acid CoA ligase (*ACSL3*). Of the 29 GO Terms related to ‘Mitochondrial Pathway’ dataset, 17 were upregulated among which featured ‘electron transport chain’ and ‘mitochondrial inner membrane’ while ‘injury to mitochondria’ was the top downregulated term (Fig. 5D and Supporting Information Table S6). Thus, it would appear that the anti-tumorigenic properties of (*R*,*S*′)-MNF correlate with sustained dysregulation in energy production and homeostasis in PANC-1 tumor xenografts even one week after (*R*,*S*′)-MNF removal.

Strikingly, we noted the presence of compensatory mechanisms that come into play to overcome metabolic deficits, such as the overexpression of genes encoding mitochondrial antioxidant enzymes (*PRDX3*, *PRDX5*) and respiratory complex subunits (e.g., *ATP5L*, *ATP5G1*, *SDHB*, *COX8A*, *NDUFB9*, *NDUFV1*) as well as the downregulation of genes implicated in aerobic glycolysis (*HIF1A*, *SLC2A1*, *LDHA*, *PFKFB4*, *WWTR1*) (Fig. 5E and Supporting Information Table S7). Canonical and nonclassical Wnt pathways were also negatively impacted by (*R*,*S*′)-MNF treatment with the downregulation of key mediators (*WNT10A*, *FZD9*, *LRP5*, *LRP10*, *LGR4*, *GSK3B*, *CTNNB1*, *TCF12*) and upregulation of the antagonist DKK1 (Fig. 5E and Supporting Information Table S7), consistent with marked attenuation in β-catenin-mediated mechanisms. (*R*,*S*′)-MNF significantly impacted various facets of tumorigenesis, including the inhibition of expression of genes encoding growth and proliferation (*GLI2*, *FOS*, *MYC*, *HB-EGF*, *EGFR*, *GRB2*), survival (*MCL1*, *BIRC2*), vascularization (*CYR61*, *VEGFB*), invasion and metastasis (*MMP7*), immune modulation and inflammation (*IL8*, *IL6ST*), and multidrug resistance (*ABCC5*, *ABCB7*, *ABCB6*) (Fig. 5E). A partial list of these genes is provided in Supporting Information Table S7.

YAP and TAZ are two transcriptional coactivators from the Hippo signaling pathway that are involved in pancreatic cancer cell survival and chemoresistance^44^. YAP/TAZ have been reported to exert positive as well as negative roles in Wnt/β-catenin pathway^45,46^. Here, (*R*,*S*′)-MNF exerted a potent repression of the Hippo-YAP/TAZ signaling in PANC-1 tumor xenografts with the downregulation of genes encoding key mediators (*TAOK1*, *MST2*, *MOBK1B*), transcriptional regulators (*WWTR1*, *TEAD4*), and downstream pro-survival genes *CYR61*, *BIRC2*, and *MCL1* (Fig. 5E and Supporting Information Table S7).

To further investigate the impact of (*R*,*S*′)-MNF beyond those specific genes and pathways, we utilized the 1890 differentially expressed transcripts and 74 metabolites (Supporting Information Table S2) as input for the multi-omics Joint Pathway Analysis (JPA), based on enrichment and topological metrics that do not account for the quantitative changes between the two experimental groups (Fig. 5F). The relevant pathways were identified as having an enrichment significance of (*P* < 0.05 [− log (*P*) > 1.3]) on the y-axis. (*R*,*S*′)-MNF treatment was linked to numerous significant pathways, such as terpenoid backbone biosynthesis, pyrimidine metabolism, synthesis and degradation of ketone bodies as well as pathways from central catabolism (e.g., TCA cycle and propanoate metabolism) and drug metabolism. Pathways linked to amino acid metabolism and degradation, and biosynthesis of glycosylphosphatidylinositol, a lipid anchor for many cell surface signaling proteins, were also influenced by (*R*,*S*′)-MNF. These data show that the actions of the drug are widespread and far-reaching.

We used the STRING v11.5 functional network association program (string-db.org) to visualize interactions between the transcribed gene products that were significantly co-expressed in response to (*R,S*′)-MNF treatment (Fig. 5G). We focused on groups of genes that are known targets of MYC (left panel) and HIF-1α (right panel). The top biological processes impacted in the MYC interactome were ‘tissue morphogenesis’ (fdr: 5.52e−08) and ‘positive regulation of gene expression’ (fdr: 9.01e−08), while the HIF-1α interactome encompassed genes enriched in processes such as ‘oxygen homeostasis’ (fdr: 0.00051) and ‘regulation of transcription from RNA polymerase II promoter in response to hypoxia’ (fdr: 1.00e−06) (Fig. 5G).

Immunoblotting experiments confirmed the significant and sustained downregulation of key pro-oncogenic signaling proteins, such as YAP, HIF-1α, c-Myc, CYR61 and β-catenin, but not cyclin D1 nor LATS1/2 in PANC-1 tumor xenografts one week after the withdrawal of (*R*,*S*′)-MNF (Fig. 5H and I and data not shown). In summary, these results show that (*R*,*S*′)-MNF is a potent inhibitor of tumor growth, eliciting its antitumorigenic effect through GPR55 and metabolic deprogramming.

## Discussion

The reprogramming of cellular metabolism towards glycolysis is a key aspect in the initiation and progression of PDAC^4,6,47–49^. HIF-1α and c-Myc play an important role in this metabolic rewiring through induction of glycolytic enzymes^5,47,50,51^. In the current study, gene expression profiling and immunoblotting analyses of PDAC-derived tumor xenografts indicate that administration of (*R*,*S*′)-MNF significantly reduced gene and protein expression of HIF-1α and c-Myc and attenuated gene expression of multiple components of the glycolytic pathway, including GLUT1 transporter (*Slc2a1*), PFKFB4, PGK2, and LDHA. Metabolomics analysis identified a significant increase in *trans*-4-hydroxyproline in (*R*,*S*′)-MNF-treated tumors consistent with degradation of HIF-1α by proline hydroxylation and subsequent ubiquitination-mediated proteasomal digestion^51,52^. We surmise that the 28% reduction in circulating l-lactate in (*R*,*S*′)-MNF-treated animals also reflects a decrease in glycolytic metabolism and that the PANC-1 tumor metabolism is shifting towards normoxic processes in response to (*R*,*S*′)-MNF administration.

Metabolic deprogramming in (*R*,*S*′)-MNF-treated tumors is also suggested by the significant reduction in the abundance of glycerolipids and drop in fatty acyl-CoA esters. PDAC tumors exhibit significant alterations in lipid metabolism^5,6,53^ and reduced mitochondrial function^4,48^. In comparison to normal pancreatic tissues, de novo lipogenesis is increased in PDAC with most of the synthesized fatty acids converted to phospholipids and triacylglycerol esters^5,53^. This shift is associated with hypoxia-driven formation of lipid droplets containing triacylglycerols and cholesterol esters^54^, with concomitant decrease in free fatty acid concentration in plasma^55^ and tumor tissues^56^ stemming from attenuated expression of lipolytic enzymes such as adipose triglyceride lipase (ATGL), encoded by *PNPLA2*^57^. ATGL is a rate-limiting lipolytic enzyme whose activity results in the release of fatty acids used for energy production (e.g., β-oxidation) and intracellular signaling, including the upregulation of genes related to mitochondrial biogenesis and β-oxidation^57^. Here, the transcriptomic analysis revealed increases in the expression of *PNPLA2*, *LPIN1* and *ACAT2* in response to (*R,S*′)-MNF treatment. *LPIN1* is a p53-controlled gene that encodes lipin-1, a phosphatidic acid phosphohydrolase that promotes fatty acid oxidation under reduced glucose conditions^58^ whereas *ACAT2* encodes acetyl-CoA acetyltransferase-2, a key player in lipid catabolism, whose expression is downregulated in PANC-1 cells^59^. Thus, the data suggests that (*R,S*′)-MNF administration deprograms glycolytic metabolism in PDAC tumors by reducing lipid storage and shifting metabolism towards fatty acid β-oxidation.

A contributing factor to this shift may be a change in glutamine metabolism as metabolic reprogramming in PDAC is associated with increased utilization of glutamine via canonical and non-canonical pathways^4,54,59–62^. In canonical glutaminolysis, glutamine conversion to glutamate leads to the formation of α-ketoglutarate, which enters the TCA cycle and ultimately provides citric acid for biosynthesis^54^. A key regulator of this process is c-Myc, which promotes the transcriptional activation of glutamine uptake and metabolism^60^. In this study, data from the metabolomics analysis indicate an intratumoral accumulation of N-acetyl-aspartyl-glutamate (NAAG) in (*R*,*S*′)-MNF-treated animals. NAAG acts as a glutamate reservoir in PDAC and delivers glutamate to the tumor after hydrolysis by the c-Myc-regulated glutamate carboxypeptidase II (GCPII)^63^. Previous studies have shown that deactivation/inhibition of c-Myc reverses the PDAC metabolic phenotype^64^ and genetic knockdown or pharmacological inhibition of GCPII suppresses glutamate production and diminishes PDAC tumor growth^63^. Thus, the accumulation of NAAG in the tumors of (*R*,*S*′)-MNF-treated mice could plausibly be associated with an impairment of glutamate metabolism. However, despite clear effect on NAAG levels, (*R*,*S*′)-MNF did not significantly alter concentration of glutamine nor glutamate. Similarly, expression of c-Myc-dependent genes associated with glutaminolysis, e.g. *SLC1A5* and *GLS*, was not affected by (*R*,*S*′)-MNF, indicating that this mechanism deserves further study and verification.

In the non-canonical glutamine pathway, glutamate is primarily used as a nitrogen donor in nucleotide and amino acid biosynthesis and mTORC1 signaling rather than as a mitochondrial substrate^59,62^. A recent metabolomic-proteomic study of the effect of glutaminase inhibitors in PDAC tumors revealed that reduced glutaminolysis resulted in increased fatty acid β-oxidation, altered response to oxidative stress, and reduced nucleotide biosynthesis^62^. The metabolomics and transcriptomic data obtained in the current study are consistent with these effects. Indeed, (*R*,*S*′)-MNF administration led to dramatic increases in the levels of ophthalmic acid and its precursor, 2-aminobutyric acid, in the tumor tissues, indicative of elevated cysteine/methionine metabolism and associated oxidative stress due to the ‘hijacking’ of the GSH-synthesizing pathway^42^. In addition, the GO Terms associated with “response to stress” and “negative regulation of apoptosis” were among the top downregulated pathways impacted by (*R*,*S*′)-MNF, likely reflecting a pathological state of redox imbalance. Moreover, tumors from (*R*,*S*′)-MNF-treated mice had significantly less orotic acid, an intermediate in pyrimidine biosynthesis, whereas the levels of uridine, uracil and β-alanine were elevated, suggesting an increase in the pyrimidine salvage pathway. These observations are consistent with increased autophagy and recycling of biochemical components that accompany glutaminolysis inhibition in PDAC^61,62,65^.

Taken as a whole, the results indicate that treatment with (*R*,*S*′)-MNF resulted in a metabolic deprogramming in PANC-1 tumors initiated by decreased expression of HIF-1α and c-Myc and subsequent attenuation of glycolysis, shift of fatty acid metabolism towards β-oxidation, and alterations in pyrimidine synthesis.

In light of the fact that reduced MEK/ERK and PI3K/ATK signaling contribute to metabolic deprogramming^66,67^, it is reasonable to assume that (*R*,*S*′)-MNF-mediated metabolic alterations are a result of upstream effects on pro-oncogenic signaling. Previous studies showed that the treatment of PDAC cell lines with GPR55 inhibitors attenuates MEK/ERK and PI3K/ATK signaling^7,15,17^. Here we demonstrate that (*R*,*S*′)-MNF dose-dependently inhibits cellular uptake of the GPR55 agonist T1117 in vitro and attenuates ERK phosphorylation in cultured PANC-1 cells treated with the GPR55 agonist O-1602.

While the GPR55 antagonist properties of (*R*,*S*′)-MNF contribute to the observed effects, the compound is also a potent, biased β_2_-AR agonist that selectively signals through Gα_s_ over β-arrestin^26^. This biased agonism at the β_2_-AR seems central for the antitumorigenic properties of (*R*,*S*′)-MNF. Although the GPR55 antagonist CBD has no affinity for the adrenergic receptors, it increases the effectiveness of gemcitabine in KPC mice, but has no significant effect on animal survival as a single agent^15^. Similarly, (*R*,*R*′)-MNF does not dampen growth of PANC-1 xenograft tumors even though it affects expression of oncogenic proteins and l-lactate production^7^. The data suggest that GPR55 inhibition alone does not hinder tumor growth and that tumor inhibition observed with (*R*,*S*′)-MNF is the cooperative effect of GPR55 antagonistic properties and biased β_2_-AR agonism leading to selective PKA activation that is not observed with the non-biased agonism produced by (*R*,*R*′)-MNF. In the current study, PANC-1 cell cultures preincubated with the PKA inhibitor PKI markedly increased basal ERK and AKT phosphorylation, a process reversed by (*R*,*S*′)-MNF treatment. Significant reductions in ERK and AKT phosphorylation were also observed after treatment of PANC-1 cells either with the Gα_s_-biased β_2_-AR agonist salmeterol, the adenylyl cyclase activator forskolin, or the β_2_-AR selective agonists (*R*,*R*′)-fenoterol and (*R*,*R*′)-MNF. The results indicate that in PANC-1 cells, β_2_-AR/Gα_s_/adenyl cyclase/PKA activity attenuates basal and β_2_-AR antagonist-mediated increase in ERK and AKT phosphorylation. This is consistent with previous studies showing the observed antiproliferative effects of (*R*,*R*′)-MNF through PKA activation in C6 glioma cells^19^.

PKA activation elicits a myriad of downstream effects including the stimulation of Lats1/2 kinase activity and the subsequent phosphorylation and deactivation of Yes-associated protein (YAP) and transcriptional coactivator with PDZ-binding motif (TAZ), known as the Hippo pathway^68–70^. Unphosphorylated YAP and TAZ translocate to the nucleus where they act as transcription co-activators with the TEAD family of transcription factors and play a role in metabolic reprogramming including glycolysis, lipogenesis, and glutaminolysis^70–74^. Two of the YAP/TEAD-regulated genes are c-Myc and cysteine-rich angiogenic inducer 61 (CYR61)^73,74^. Here, the mRNA expression and protein abundance of c-Myc and CYR-61 were reduced in PANC-1 xenografts of (*R*,*S*′)-MNF-treated mice relative to vehicle-treated controls along with significant reduction in YAP protein in the treated tumors. The effect on CYR61 expression is of interest as increased CYR61 expression is associated with PDAC aggressiveness, drug resistance, and tumor-microenvironment interactions^75–77^.

**Figure 6:**
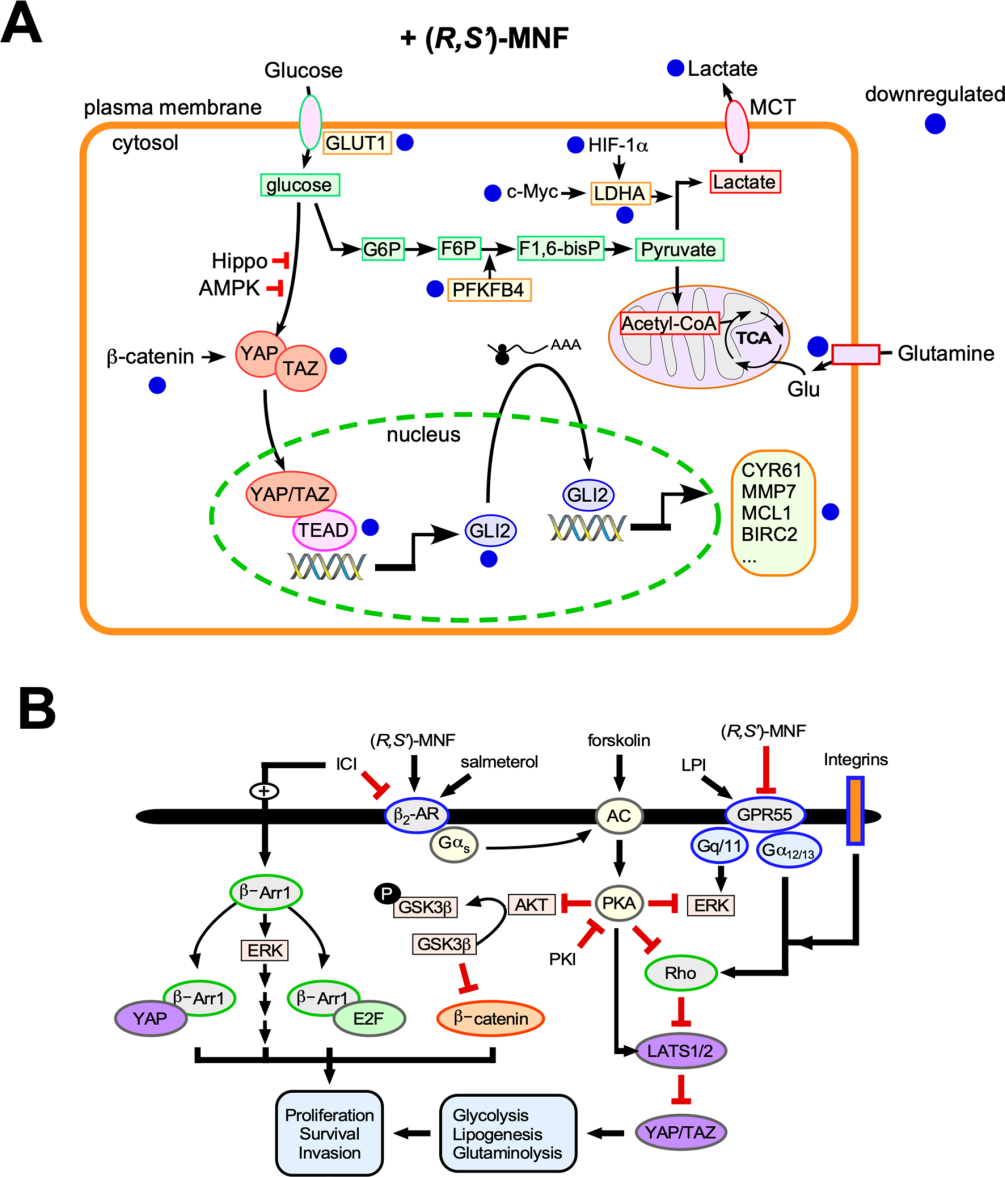
(*R*,*S*′)-MNF blocks the growth of PANC-1 xenograft tumors in mice. (**A**) YAP/TAZ is regulated by the canonical Hippo pathway, AMPK-dependent phosphorylation, and the β-catenin destruction complex. YAP/TAZ translocation to the nucleus and interaction with TEAD transcription factors induces target genes such as *GLI2*, which encodes a transcription factor that controls the expression of several genes implicated in tumorigenesis (e.g., *CYR61*, *MMP7*, *MCL1*, *BIRC2*). Administration of (*R*,*S*′)-MNF led to significant downregulation in the expression of the indicated protein-encoded genes. Inhibition of the glycolytic pathway combined with reduced expression of *LHDA*, caused by the downregulation of c-Myc and HIF-1α gene and protein levels, negatively impacts the production of l-lactate by the tumor and its release into the circulation. Inhibition of glutamine uptake and its conversion to glutamate has a negative impact on the production of metabolic intermediates within the TCA cycle for mitochondrial ATP production. (**B**) Bitopic function of (*R*,*S*′)-MNF contributes to its anti-tumor activity. (*R*,*S*′)-MNF is a biased agonist of the β_2_-AR by promoting its coupling to the Gα_s_/adenylate cyclase (AC)/cAMP/PKA signal transduction, while preventing the recruitment and activation of β-arrestin 1, a critical regulator of tumorigenesis through ERK activation and formation of protein complexes with YAP and E2F transcription factors. The selective β_2_-AR antagonist, ICI 118551, inhibits salmeterol-stimulated cAMP production and associated PKA activity, while promoting basal β-arrestin 1 function. PKA blocks AKT- and ERK-mediated phosphorylation of target proteins and enhances LATS-dependent inhibition of YAP/TAZ pro-tumorigenic activity. LATS1/2 is activated by PKA, but inactivated by mechanical cues (e.g., integrin engagement) and GPCR-RhoA-mediated F-actin. Binding of the bioactive lipid, lysophosphatidylinositol (LPI), to GPR55 activates signaling cascades implicated in cell proliferation, invasion and survival through coupling to Gα_q/11_ and Gα_12/13_. Binding of (*R*,*S*′)-MNF to GPR55 directly inhibits its function. The illustrations were created using Canvas X Draw v.7.0.2 for macOS (Canvas GFX, Boston, MA).

While PKA is associated with activation of Lats1/2 kinase and inhibition of YAP/TAZ signaling, there are multiple pathways that produce the opposite effect^70^. One of these pathways involves signaling through Gα_12_ and Gα_q_ protein-coupled receptors, which activate RhoA GTPase and F-actin leading to Lats1/2 inhibition^68,78,79^. Because GPR55 signals through both Gα_12_ and Gα_q_, it is reasonable to assume that (*R*,*S*′)-MNF and any other GPR55 inhibitors will contribute to a reduction in YAP/TAZ-associated transcriptional regulation. We surmise that the dual role of (*R*,*S*′)-MNF, both as a GPR55 inhibitor and β_2_-AR agonist, contributes to reduced YAP/TAZ activity through Lats1/2, as illustrated in Fig. 6. This assumption is under investigation and the data will be reported elsewhere.

Although the Hippo pathway has received a great deal of attention, additional activation mechanisms for YAP/TAZ signaling have been identified in PDAC, including the PI3K/AKT/mTORC1 pathway^70,74^. This pathway is of particular interest as incubation of PANC-1 cells with (*R*,*S*′)-MNF significantly reduced the activating phosphorylation of AKT. In addition, incubation of PANC-1 and MIA PaCa-2 cell cultures with forskolin resulted in PKA-associated phosphorylation of the mTORC1 component Raptor to negatively regulate mTORC1^80^. Interestingly, (*R*,*S*′)-MNF may also attenuate mTORC1 activity through reduction in orotic acid, a metabolite previously shown to activate mTORC1 via negative regulation of AMPKA^81^. The focus of ongoing investigations is on gaining valuable insights into the effect of (*R*,*S*′)-MNF on mTORC1 activity.

One reflection of decreased mTORC1 signaling may be the increased abundance of phospho-eukaryotic elongation factor 2 (peEF2) observed in (*R*,*S*′)-MNF-treated cells. eEF2 plays a key role in protein synthesis when unphosphorylated and eEF2 kinase (eEF2K) mediates phosphorylation-dependent eEF2^82,83^. eEF2K is activated by β_2_-AR/PKA signaling in cardiomyocytes and melanoma cells^21,84^ and by the inhibition of mTORC1/p70S6K cascade^82,83^, the latter also responsive to β_2_-AR/Gα_s_/adenylyl cyclase/PKA signaling. Like (*R*,*S*′)-MNF, salmeterol treatment of PANC-1 cells resulted in higher peEF2 levels, whereas (*R*,*R*′)-MNF and (*R*,*R*′)-fenoterol had no effect, indicating that a multitude of signaling pathways are likely involved in mediating (*R*,*S*′)-MNF-associated increases in eEF2 phosphorylation.

Because eEF2K activity is also inhibited by activation of the MEK/ERK/p90RSK cascade^82,83^, a reduction in MEK/ERK signaling would be expected to increase peEF2 levels. However, our data indicate that (*R*,*S*′)-MNF did not significantly alter MEK/ERK activity (even though peEF2 levels were up) in PANC-1 cell cultures, whereas inhibition of MEK/ERK was achieved with either salmeterol, (*R*,*R*′)-MNF or (*R*,*R*′)-fenoterol treatment, with salmeterol being the only β_2_-AR ligand capable at impacting peEF2 levels. Such divergence among β_2_-agonists may arise from differences in interactions with β-arrestin and subsequent β-arrestin-mediated ERK activation. Both (*R*,*S*′)-MNF and salmeterol are Gα_s_-biased β_2_-AR agonists that do not cause β-arrestin recruitment while (*R*,*R*′)-MNF and (*R*,*R*′)-fenoterol weakly recruit β-arrestin to β_2_-AR^26,38^. The inhibition and/or non-engagement of β-arrestin signaling is intriguing as β-arrestin is involved in multiple protein interaction networks involved in tumor development, metastasis, and transcriptional regulation^85–87^. It is important to note that GPR55 activation also results in β-arrestin recruitment to this GPCR and associated increases in MEK/ERK signaling^88,89^. Both (*R*,*S*′)-MNF and (*R*,*R*′)-MNF effectively inhibit GPR55 activation, which may be the source of some of their effects on tumor growth and viability. The data suggest that the increase in peEF2 levels by (*R*,*S*′)-MNF depends on β_2_-AR/Gα_s_/adenylyl cyclase/PKA signaling in the absence of β-arrestin recruitment to the β_2_-AR.

The current study demonstrates that (*R*,*S*′)-MNF has the potential to provide a unique contribution to the treatment of PDAC, which is currently under investigation using patient-derived xenograft (PDX) models with (*R*,*S*′)-MNF administered as a single agent and in combination with standard of care therapies. Taken as a whole, the data obtained from the metabolomics and transcriptomics analysis of PANC-1 tumor tissues indicate that treatment with (*R*,*S*′)-MNF resulted in a metabolic deprogramming characterized by decreased glycolysis, alterations in fatty acid metabolism, and pyrimidine synthesis, and that these changes are consistent with reduced expression of HIF-1α and c-Myc. The results from studies utilizing PANC-1 cell cultures are consistent with the tumor-derived data and indicate that the bifunctional properties of (*R*,*S*′)-MNF as GPR55 antagonist and β_2_-AR-biased agonist both contribute to the observed antitumor effects (Fig. 6). As with other multi-omics studies, the data has provided more questions than answers. Key issues such as the effect of (*R*,*S*′)-MNF on β-arrestin and mTORC1 signaling were identified and are currently being explored.

## Supplementary Information

Supplementary Information.

## Data Availability

The raw data file and the filtered, normalized microarray gene expression results are available online in the Gene Expression Omnibus, Accession Number GSE113077. Remaining data and materials that support the findings of this study are available from the corresponding authors upon reasonable request. Some data may not be made available because of privacy or ethical restrictions.
