# Supplementary material for: Deprogramming metabolism in pancreatic cancer with a bi-functional GPR55 inhibitor and biased β2 adrenergic agonist
Source: Sci Rep. 2022 Mar 7;12:3618. doi: 10.1038/s41598-022-07600-x (PMC8901637; doi:10.1038/s41598-022-07600-x)

SUPPORTING INFORMATION
for

Deprogramming metabolism in pancreatic cancer with a bi-functional GPR55 inhibitor and biased β_2-adrenergic agonist

Artur Wnorowski[*†], Danuta Dudzik[*], Michel Bernier, Jakub Wójcik, Guido Keijzers, Alberto Diaz-Ruiz, Karolina Mazur, Yongqing Zhang, Haiyong Han, Morten Scheibye-Knudsen, Krzysztof Jozwiak, Coral Barbas, Irving W. Wainer[†]

[*] Contributed equally to the work.

[†] Correspondence: Artur Wnorowski, email: artur.wnorowski@umlub.pl; Irving W. Wainer, iwwainer@gmail.com.

TABLE OF CONTENTS

Online Materials and Methods 2

Supporting Figure S1. (*R*,*S*′)-MNF inhibits GPR55 binding activity of the fluorescent ligand Tocrifluor 1117 (T1117). 6

Supporting Figure S2. Effects of β_2-AR agonists and adenylate cyclase activator on key cellular signaling cascades in PANC-1 cells. 7

Supporting Figure S3. Metabolomics analysis on the effect of (*R*,*S*′)-MNF administration in PANC-1 xenograft tumors. 8

Table S1. Effect of the i.p. administration of (*R*,*S*′)-MNF on body weight and volume of PANC-1-derived tumors in a mouse xenograft model 9

Table S2. List of statistically significant metabolites identified in the study. 10

Table S3. Compounds quantified by targeted metabolomics. 12

Table S4. Metabolite signature of PANC-1 xenograft tumor in mice treated with (*R*,*S*′)-MNF. 13

Table S5. GO Term signature of (*R*,*S*′)-MNF treatment of PANC-1 xenograft tumor in mice. 14

Table S6. Mitochondrial pathway signature of (*R*,*S*′)-MNF treatment of PANC-1 xenograft tumor in mice. 15

Table S7. Gene expression signature of (*R*,*S*′)-MNF treatment of PANC-1 xenograft tumor in mice. 16

Full-size blots 17

Online Materials and Methods

Sample preparation and multiplatform metabolomics study

I) Samples preparation.
The method for sample preparation was adapted from González-Peña *et al*. 2017.
Homogenization
All samples were stored at -80°C until analysis. In the day of analysis, the samples were immersed in LN2 and a representative cross-section of tissue was cut and weighted. A mixture of cold (-20°C MeOH/H_2O (1:1) in ratio 1:10) was added to each sample. The exact volume of the extraction solution was adjusted according to weight of the sample. Tissue lysis and metabolite extraction was carried out with QIAGEN TissueLyser LT bead-mill homogenizer. The process was performed by using 5 mm (mean diameter) stainless steel beads, vibrating at 50 Hz for 5 min. Three repeated cycles with 1 min break were applied with samples and TissueLyser adapter cooled on ice. Additionally, ultrasounds (UP 200S Ultrasonic lab homogenizer, Hielscher GmbH) were introduced. Parameters of sonication were set as follow: 50 amplitudes, 0.8 cycles, sonication time 30 sec. The process was performed on ice.

Metabolites extraction for LC-MS analysis
Homogenate (100 µL) was reconstituted in 320 µL of MeOH and vortex-mixed for 15 min. Subsequently 80 µL methyl tert-butyl ether (MTBE) was added and vortex-mixed 1 h at room temperature. Samples were centrifuged for 20 min at 4000 × g at 20°C. Hundred microliters (100 µL) of supernatant was used for LC-MS analysis.

Metabolites extraction for GC-MS analysis
Homogenate (150 µL) protein was precipitated with cold methanol (300 µL) and separated by centrifugation (16000 × g, 15 min, 4°C). Resulting supernatant was transferred to GC vial with insert and then evaporated to dryness (Speedvac Concentrator (Thermo Fisher Scientific). Twenty microliters (20 µL) of O-methoxyamine hydrochloride in pyridine (15 mg/mL) was added to each GC vial, and mixture was vigorously vortex-mixed and ultrasonicated. Methoxymation was carried out in darkness at room temperature for 16 h. N, *O*-Bistrifluoroacetamide (BSTFA) with 1% chlorotrimethylsilane (TMCS) (20 µL) was then added as catalyst. For silylation process samples were heated in an oven for 1 h at 70°C. Finally, 100 µL of heptane containing 10 ppm of C18:0 methyl ester as internal standard (IS) was added to each GC vial and vortex-mixed before GC analysis.

Metabolites extraction for CE-MS analysis
Two hundred microliters (200 µL) of homogenate were vortex-mixed with 200 µL of 0.2 M formic acid, centrifuged (16000 × g 10 min, 4°C) and transferred to a centrifree ultracentrifugation device (Millipore Ireland Ltd., Ireland) with 30 kDa protein cutoff for deprotenization through centrifugation (2000 × g, 70 min, 4°C). The filtrate was then transferred to the chromacol vial, dried using a SpeedVac, and resuspended in 100 µL of 0.1 M formic acid with 0.2 mM methionine sulfone (IS) before CE-MS analysis.

QC sample preparation
Quality control (QC) samples were prepared by pooling equal volumes of pancreatic cancer tissue homogenate from each of the 20 samples. Five QC samples were independently prepared for each technique following the same procedure as applied for the experimental samples. QC samples were analyzed throughout the run to provide a measurement of the system's stability, performance, and the reproducibility of the sample treatment procedure.

Randomization
The samples were randomized before homogenization, metabolite extraction and for analytical run.

II) Metabolic fingerprinting.
LC–QTOF/MS
A HPLC system (1200 series, Agilent Technologies, Waldbronn, Germany), equipped with a degasser, two binary pumps, and a thermostated autosampler coupled with Q-TOF LC/MS (6520) system (Agilent), was used in the ESI+ and ESI− mode to increase the number of detected metabolite ions.

Briefly, 5 µL of extracted pancreatic tissue samples was injected into a thermostated (60°C) Agilent Poroshell 120 EC-C8 column (150 mm × 2.1 mm, 2.7 µm) with a guard column Ascentis® Express C8 (5 mm × 2.1 mm, 2.7 µm). The flow rate was 0.5 mL/min with solvent A (5 mM ammonium formate in Milli-Q water), and solvent B (5 mM ammonium formate in methanol) for analysis in positive ionization mode and solvent A (water with 0.1% formic acid), and solvent B (methanol with 0.1% formic acid and 15% isopropanol) for analysis in negative ionization mode. Initial conditions at time 0 were 82% B, increasing to 96% B in 30 min. This was then held until 38 min. The gradient then increased to 100% B by 38.5 min and held until 40.5 min. The starting condition was returned to by 42 min, followed by an 8-min re-equilibration time, taking the total run time to 50 min. Capillary voltage was set to 3.5 kV for positive and 4.5 kV for negative ionization mode; the drying gas flow rate was 12 L/min at 250°C and gas nebulizer at 52 psi; fragmentor voltage was 175 V for positive and 125 V for negative ionization mode; skimmer and octopole radio frequency voltage (OCT RF Vpp) were set to 65 V and 750 V, respectively. Data were collected in the centroid mode at a scan rate of 1.2 spectrum per second. Mass spectrometry detection was performed in both positive and negative ESI mode in full scan from 100 to 1200 *m/z*. The reference mass ions used were 121.050873, 922.009798 (positive ion mode) and 119.036320, 966.000725 (negative ion mode). These masses were continuously infused into the system to allow constant mass correction. Samples were analyzed in separate runs (positive and negative ionization modes), in a randomized order.

GC–Q-MS

A GC system (Agilent Technologies 7890A), equipped with an autosampler (Agilent 7693) and interfaced to an inert mass spectrometer with triple-Axis detector (5975C, Agilent), was used for pancreatic tissue cancer fingerprinting. Briefly, 2 µL of the derivatized sample was injected in a GC column DB5-MS (30 m length, 0.25 mm, 0.25 µm film 95% dimethyl/ 5% diphenylpolysiloxane) coupled to a pre-column (10 m Agilent J&W Capillary GC column integrated with Agilent 122-5532G). The injector port was held at 250°C, and the helium carrier gas flow rate was set at 1.0 mL/min. The split ratio was 1:10. The temperature gradient was programmed as follows: the initial oven temperature was set to 60°C (held for 1 min), increased to 325°C at a rate of 10°C/min; the system was allowed to cool down for 10 min before the next injection. The detector transfer line, the filament source and the quadrupole temperature were set to 280°C, 230°C and 150°C, respectively. MS detection was performed in electron impact (EI) mode at −70 eV. The mass spectrometer was operated in scan mode over a mass range of 50–600 m/z at a rate of 2.7 scan/s.

CE-TOF-MS

An Agilent 7100 (CE) system, coupled to a TOF Mass Spectrometer (6224 Agilent), was used for sample analysis. In brief, a fused-silica capillary (Agilent Technologies; total length, 96 cm; i.d., 50 µm) was pre-conditioned with 1 M NaOH for 30 min, followed by MilliQ® water for 30 min and background electrolyte (BGE; 0.8 M formic acid in 10% methanol) for 30 min. Before each analysis, the capillary was flushed for 5 min (950 mbar pressure) with BGE. The MS was operated in positive polarity, with a full scan from 80 to 1000 m/z at a rate of 1.4 scan/s. Drying gas was set to 10 L/min, nebulizer to 10 psi, voltage to 3.5 kV, fragmentor to 125 V, gas temperature to 200°C and skimmer to 65 V. The sheath liquid composition was methanol/water (1/1, v/v), containing 1.0 mM formic acid with two reference masses (121.050873 - purine (C5H4N4) and 922.009798 - HP-921 (C18H18O6N3P3F24)), which allows for correction and provides more accurate mass determination. Flow rate was 0.6 mL/min and split was set to 1/100. Samples were injected at 50 mbar for 50 s. After each injection, along with the samples, BGE was co-injected for 10 s at 100 mbar pressure to improve repeatability. Separations were performed at a pressure of 25 mbar and a voltage of +30 kV; current under these conditions was 100 µA.

III) Data treatment and statistical analysis.

Data Acquisition

Quality of the raw data was first inspected by the analysis of the chromatograms/electrochromatograms acquired for experimental samples, QC samples, blanks and internal standard if used. Raw data acquired were processed to provide structured data in an appropriate format for data analysis. The data collected by LC-MS and CE-MS were cleaned of background noises and unrelated ions in recursive analysis in Mass Hunter Profinder (B.06.00, Agilent Technologies) software. Feature extraction is the reduction of acquired data size and complexity through the removal of redundant and non-specific information by identifying the important variables (features) associated with the data. Molecular feature extraction (MFE) performs chromatographic deconvolution to find the features in the analyzed samples. The features are aligned across all of the selected sample files using mass and retention/migration time. Recursive Feature Extraction first performs MFE and

then uses the MFE results, feature mass and retention time, to perform a targeted feature extraction referred to as Find by Ion (FbI). Find by Ion uses the median mass, median retention time, and composite spectrum calculated from the aligned features to improve the reliability in finding the features in the data. GC-MS raw data files were translated to appropriate format with MassHunter Workstation GC/MS Translator (B.04.01, Agilent Technologies) and then processed with the MassHunter Quantitative (B.08.00, Agilent Technologies) software. Deconvolution process and metabolite identification was conducted applying Agilent MassHunter Unknowns Analysis Tool 7.0.

Data normalization and filtration
Quality assurance procedure was applied to check overall data quality (Dudzik *et al.* 2017). Non-supervised PCA-X projection method was used to evaluate instrumental signal drift, sensitivity loss and variation of the measurement in QC samples. The control Shewhart´s charts were used to plot the sum of acquired signals of detected metabolic features for every analyzed sample against the acquisition order, that enables for fast detection of the measurement abnormalities. When required, the data were normalized. Normalization according to internal standard, methionine sulfone was applied to CE-MS data and normalization by fold change to correct GC-MS data. Variation of the compound concentrations in QC samples expressed as coefficient of variation (%CV) was also calculated. For data filtration, a threshold of 20% for LC-MS and CE-MS and 30% for GC-MS was set for the CV values of metabolites in the QC samples.

Statistical analysis
Data normality was verified by evaluation of the Kolmogorov−Smirnov−Lillefors and Shapiro−Wilk tests and variance ratio by the Levene's test. Differences between experimental groups were evaluated by unpaired t test (equal or unequal variance) or nonparametric (Mann−Whitney test) with post hoc Benjamini Hochberg (FDR) correction. The levels of statistical significance were set at 95% level ($P < 0.05$). Statistical analyses were performed using Matlab R2015 (Mathworks) software. MetaboAnalyst v5.0 data annotation tool (metaboanalyst.ca) was used for testing the relationships between variables (Xia *et al*., 2016). Multivariate (unsupervised and supervised) analysis as well as other multivariate calculation and plots was performed by using SIMCA-P+ 14.0 (Sartorius AG/Umetrics, Umea, Sweden; sartorius.com). Combination of VIP-p(corr) (correlation coefficient combined with VIP, Variable Influence on the Projection) based on selected OPLS-DA, with the cutoff set as VIP ≥ 1.0 and p(corr) ≥ 0.4 model was applied for specified interpretations.

IV) Compounds Identification
Accurate mass of statistically significant metabolic features was searched for possible ID against the online available databases as Kegg, Metlin, LipidMaps and HMDB using online available advanced CEU Mass Mediator (CMM) tool (Gil de la Fuente *et al*., 2018). Isotopic distribution, accurate m/z, retention/migration time for each metabolite feature (LC-MS and CE-MS) as well as fragmentation spectra obtained from LC-MS/MS analysis and analysis of commercially chemical standards if available have been studied for final metabolite identification. Compound identification by GC-MS was performed with the target metabolite Fiehn GC/MS Metabolomics RTL library (G1676AA, Agilent), the CEMBIO-library and the NIST 14 Mass Spectral Library (Babushok *et al*., 2007).

V) Experiment Validation
OPLS/O2PLS-DA models that were obtained according to multivariate calculations were validated by cross-validation tool. Validation was performed by using the leaving-1/3-out approach. A randomized data set was divided into three parts, and 1/3 of samples were excluded to build a model with the remaining 2/3 of samples. Then, the excluded samples were predicted by the new model, and the process was repeated until all samples have been predicted at least once. Each time the percentage of correctly classified samples was calculated. In the models obtained with data from LC–MS, 63% (ESI+) and 90% (ESI−) of all excluded samples were classified correctly; in data from GC–MS and CE–MS, 80% and 50%, respectively, of the excluded samples were classified correctly. To estimate the predictive power of statistically significant metabolites the multivariate model was created and validated resulting in 92% correct classification of the samples.

Microarray Analysis
For the calculation of pairwise distances between samples, each microarray was considered as a point in a high-dimensional space since we treated each probe as a variable. For parametric analysis of gene set enrichment (PAGE), our expression data was tested using the PAGE method as previously described (Kim and Volsky, 2005). Briefly, for each pathway under each pair

of conditions, an aggregated Z score and *P* value were computed (JMP 6.0 software) to the total Z-ratio in comparison by Z-test. Ingenuity Pathways Analysis© was performed by using the tools supplied by Ingenuity Inc. (Ingenuity Systems; Redwood City, CA).

References

Kim SY, Volsky DJ. PAGE: parametric analysis of gene set enrichment. *BMC Bioinformatics* 2005;6:144.

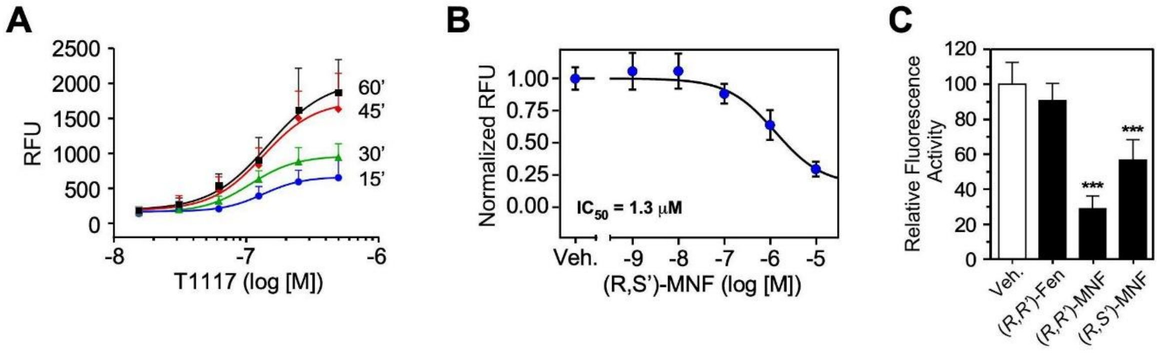

Supporting Figure S1. (*R*,*S′*)-MNF inhibits GPR55 binding activity of the fluorescent ligand Tocrifluor 1117 (T1117). ***(A)*** HEK293 cells ectopically expressing recombinant human GPR55 (hGPR55-HEK293) were incubated with various concentrations of T1117 for periods up to 60 min. Total cellular fluorescence was then measured and expressed as relative fluorescence units (RFU). Data originate form 3 independent experiments carried out in septuplicate. ***(B)*** Serum-depleted hGPR55-HEK293 cells were preincubated with increasing concentrations of (*R*,*S′*)-MNF for 30 min prior to the addition of 100 nM T1117. N = 3 independent experiments; 4 replicates per experiment. ***(C)*** Serum-depleted hGPR55-HEK293 cells were preincubated for 30 min with 1 μM of the indicated fenoterol derivatives followed by a 30-min incubation with T1117. N = 3 independent experiments. *** $P < 0.001$ vs. vehicle control. All graphs were generated with GraphPad Prism v.8.4.3 (GraphPad Software, Inc., La Jolla, CA).

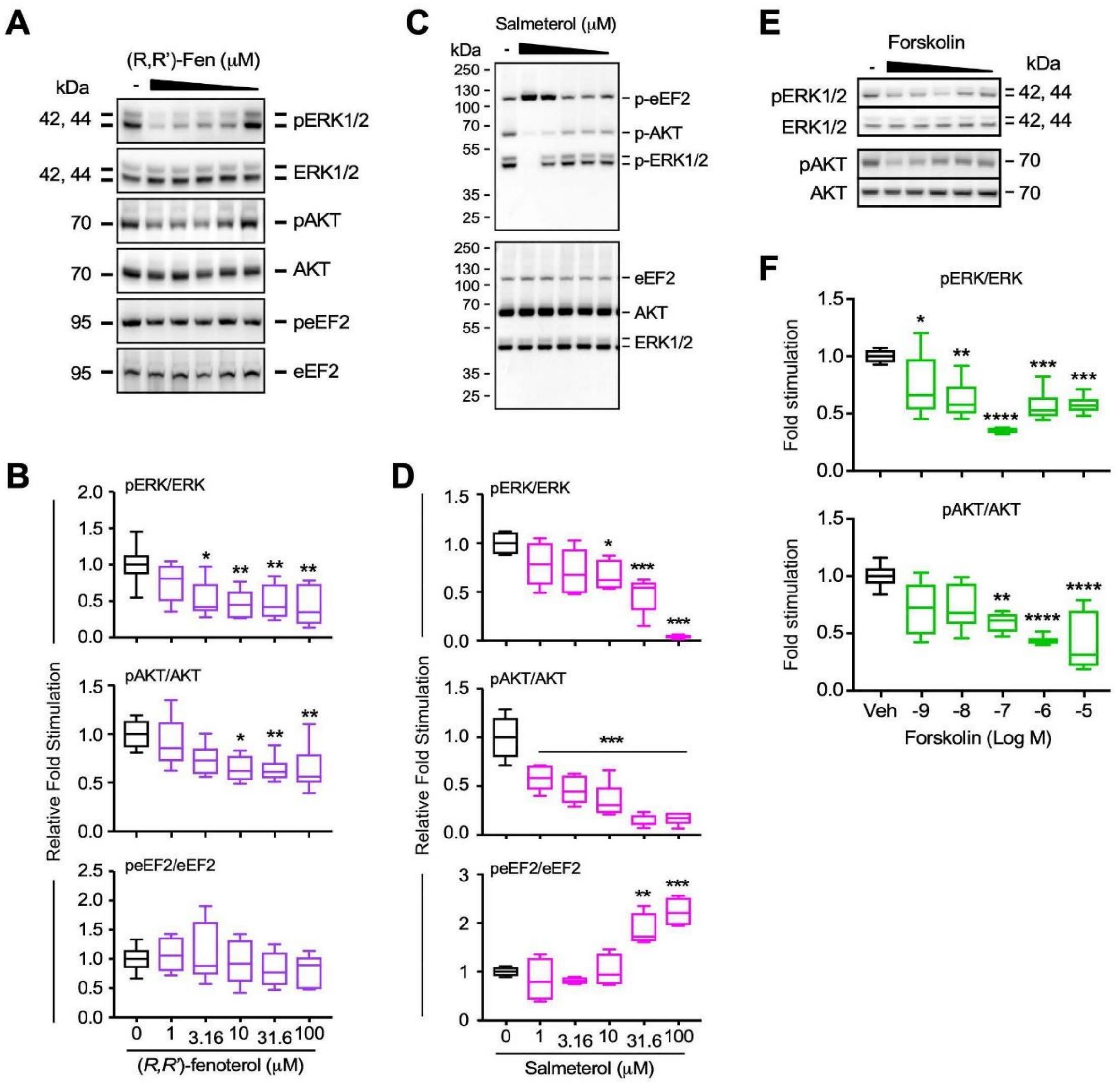

Supporting Figure S2. Effects of β2-AR agonists and adenylate cyclase activator on key cellular signaling cascades in PANC-1 cells.

Serum-starved PANC-1 cells were incubated with increasing concentrations of (*R*,*R*′)-Fen (***A***, representative blots; ***B***, densitometry), salmeterol (***C***, representative blots; ***D***, densitometry), or forskolin (***E***, representative blots; ***F***, densitometry) for 20 min. Cell lysates were resolved by SDS-PAGE under reducing conditions and immunoblotted with primary antibodies raised against phospho-specific and total forms of ERK, AKT and eEF2. Data analysis: n = 6 per group, one-way ANOVA followed by Tukey's post-hoc test; *, **, ***, **** $P < 0.05, 0.01, 0.001, 0.0001$ vs. vehicle control. All box plots were generated with GraphPad Prism v.8.4.3 (GraphPad Software, Inc., La Jolla, CA).

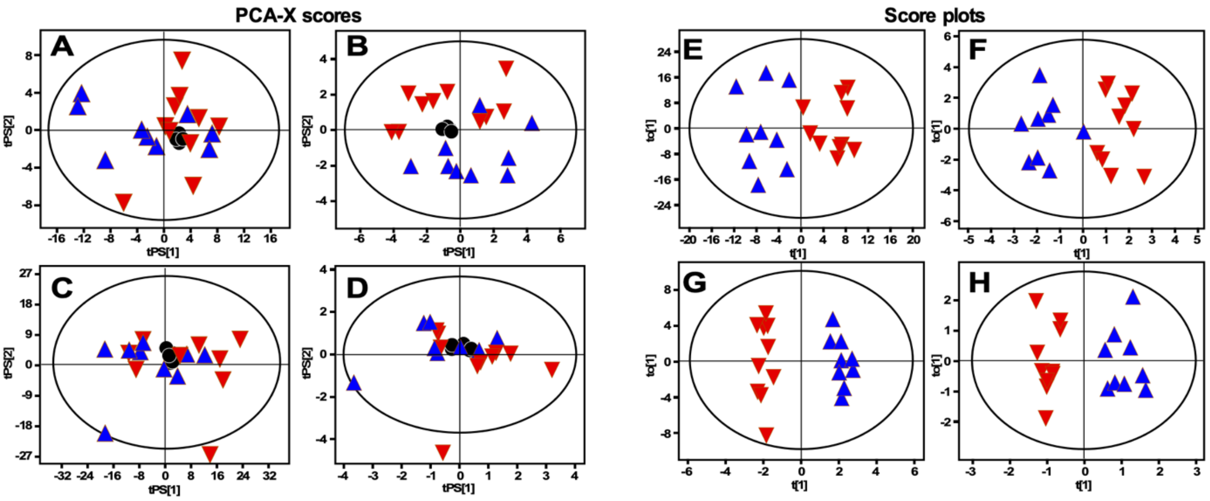

Supporting Figure S3. Metabolomics analysis on the effect of (*R*,*S′*)-MNF administration in PANC-1 xenograft tumors. ***(A-D)*** PCA-X score plots of experimental samples (▲, blue triangles, (*R*,*S′*)-MNF-treated pancreatic tissue cancer; ▼, red invert triangles, samples from non-treated mice; ●, black circles, predicted QC samples). ***(A)*** LC-MS ESI(+); ***(B)*** LC-MS ESI(−); ***(C)*** CE-MS; ***(D)*** GC-MS data. PCA-X score plots were created in SIMCA-P+ 14.0 (Sartorius AG/Umetrics, Umea, Sweden; sartorius.com). ***(E-H)*** Score plots of pancreatic cancer tissue metabolic profiles obtained for non-treated and treated with (*R*,*S′*)-MNF samples. Supervised OPLS/O2PLS-DA analysis. ***(E)*** LC-MS ESI(+) (R2 = 0.43, Q2 = 0.042); ***(F)*** LC-MS ESI(-) R2 = 0.84, Q2 = 0.55); ***(G)*** CE-MS (R2 = 0.97, Q2 = 0.23), ***(H)*** GC-MS (R2 = 0.92, Q2 = 0.63). R2, coefficient for variance explained; Q2, coefficient for variance predicted. OPLS-DA score plots were created in SIMCA-P+ 14.0.

Table S1. Effect of the i.p. administration of (*R*,*S*′)-MNF on body weight and volume of PANC-1-derived tumors in a mouse xenograft model.

Group	Body weight (g)	Body weight change (%)	Tumor volume (mm^3)	Tumor volume change (%)
**Control**				
Day 8 (n = 10)	21.5 ± 0.3	-	142 ± 8	-
Day 26 (n = 10)	22.1 ± 0.3	103 ± 2	752 ± 57	558 ± 69
Day 29 (n = 10)	21.9 ± 0.4	102 ± 1	875 ± 67	651 ± 85
Day 33 (n = 10)	21.9 ± 0.4	102 ± 2	957 ± 79	716 ± 94
**Arm 1**				
Day 8 (n = 10)	21.3 ± 0.2	-	143 ± 8	-
Day 26 (n = 10)	20.6 ± 0.3	97 ± 1	399 ± 41	280 ± 25
Day 29 (n = 5)	20.1 ± 0.4	95 ± 1	457 ± 32	311 ± 59
Day 33 (n = 5)	20.2 ± 0.6	96 ± 2	615 ± 73	416 ± 49
**Arm 2**				
Day 8 (n = 10)	21.3 ± 0.2	-	143 ± 8	-
Day 26 (n = 10)	19.8 ± 0.4	93 ± 2	294 ± 38	205 ± 22
Day 29 (n = 10)	19.4 ± 0.6	91 ± 3	343 ± 32	242 ± 20
Day 33 (n = 10)	19.2 ± 0.7	90 ± 4	362 ± 33	259 ± 27

The experimental groups are vehicle control for (*R*,*S*′)-MNF (**Control**), 20 mg·kg^{-1} (*R*,*S*′)-MNF (**Arm 1**), and 40 mg·kg^{-1} (*R*,*S*′)-MNF (**Arm 2**). The presented data was collected before initiation of treatment (Day 8), at the end of the third cycle of drug administration on Day 29 and at the end of the study on Day 33.

Table S2. List of statistically significant metabolites identified in the study.

Compound ID	Formula	Mass	MT /RT	Platform	Error	pcorr	VIP	*P*-value	% Change	FC	CV QC
L-Carnitine^{STD}	$C_7H_{15}NO_3$	161.1052	9.0	CE-MS	2	0.7	1.6	0.00114#	17	1.17	5
Acetylcarnitine^{STD}	$C_9H_{17}NO_4$	203.1158	9.4	CE-MS	6	-0.5	1.3	0.04711	-14	-1.16	4
Propionylcarnitine^{STD}	$C_{10}H_{19}NO_4$	217.1314	9.5	CE-MS	2	0.5	1.7	0.04258	27	1.27	7
Stearoylcarnitine^{STD}	$C_{25}H_{49}NO_4$	427.3662	1.1	LC-MS ESI(+)	0	0.6	1.7	0.01797#	-33	-1.49	4
Hydroxybutyrylcarnitine	$C_{11}H_{21}NO_5$	247.1420	9.9	CE-MS	4	-0.5	1.7	0.03139	-20	-1.25	4
Methyl oleate	$C_{19}H_{36}O_2$	296.2715	19.3	GC-MS	n.a.	-0.6	2.2	0.02667	-42	-1.72	18
Linoleic acid^{STD}	$C_{18}H_{32}O_2$	280.2402	20.3	GC-MS	n.a.	-0.6	1.6	0.01816	-22	-1.29	18
Trans-4-hydroxyproline^{STD}	$C_5H_9NO_3$	131.0582	11.2	CE-MS	13	0.9	3.0	8.8E-07#	53	1.53	7
beta Alanine^{STD}	$C_3H_7NO_2$	89.04768	8.6	GC-MS	n.a.	0.5	1.6	0.03499	33	1.33	17
Isoleucine^{STD}	$C_6H_{13}NO_2$	131.0946	10.1	GC-MS	n.a.	-0.4	1.1	0.04347	-13	-1.14	3
L-Aspartic acid^{STD}	$C_4H_7NO_4$	133.0375	11.0	CE-MS	3	0.5	1.6	0.01749	22	1.22	3
Acetylaspartylglutamate	$C_{11}H_{16}N_2O_8$	304.0907	15.7	CE-MS	4	0.5	1.5	0.03526	24	1.24	7
Ophthalmic acid^{STD}	$C_{11}H_{19}N_3O_6$	289.1274	11.9	CE-MS	2	0.8	4.4	0.00018#	237	3.37	8
2-Aminobutyric acid^{STD}	$C_4H_9NO_2$	103.0633	9.8	CE-MS	5	0.6	2.8	0.00064#	84	1.84	4
Uracil^{STD}	$C_4H_4N_2O_2$	112.0273	15.3	CE-MS	3	0.6	1.2	0.00682	10	1.10	2
Orotic acid^{STD}	$C_5H_4N_2O_4$	156.0171	15.6	GC-MS	n.a.	-0.8	2.4	0.00192	-36	-1.56	8
Uridine^{STD}	$C_9H_{12}N_2O_6$	244.0695	15.4	CE-MS	6	0.7	2.0	0.00166#	26	1.26	6
Adenosine^{STD}	$C_{10}H_{13}N_5O_4$	267.0968	11.3	CE-MS	1	0.7	2.3	0.00078#	36	1.36	11
DG(36:3)	$C_{39}H_{70}O_5$	618.5223	14.8	LC-MS ESI(+)	3	0.9	1.9	0.01774	-41	-1.69	13
TG(36:0)	$C_{39}H_{74}O_6$	638.5485	21.1	LC-MS ESI(+)	6	0.7	1.7	0.02595	-49	-1.96	6
TG(54:3)	$C_{57}H_{104}O_6$	884.7833	35.6	LC-MS ESI(+)	0	0.9	1.7	0.03229	-42	-1.72	12
PE(38:4)	$C_{43}H_{78}NO_7P$	751.5516	14.1	LC-MS ESI(+)	1	0.7	2.0	0.00641	-23	-1.30	17
PE(40:4)	$C_{45}H_{82}NO_7P$	779.5829	16.7	LC-MS ESI(+)	1	0.6	1.7	0.02019	-22	-1.29	5
PC(14:0/16:1)	$C_{38}H_{74}NO_8P$	703.5152	5.3	LC-MS ESI(-)	3	-0.8	2.4	0.000607#	33	1.33	2
PC(16:0/16:1)	$C_{40}H_{78}NO_8P$	731.5465	7.7	LC-MS ESI(-)	1	-0.8	2.3	0.00026#	29	1.29	2
PC(16:1/22:6)	$C_{46}H_{78}NO_8P$	803.5465	6.1	LC-MS ESI(-)	0	-0.6	1.6	0.00822#	18	1.18	5
PC(16:1/20:4)	$C_{44}H_{78}NO_8P$	779.5465	6.6	LC-MS ESI(-)	1	-0.7	1.8	0.00023#	18	1.18	2
PC(14:0/18:2)	$C_{40}H_{76}NO_8P$	729.5309	5.9	LC-MS ESI(-)	1	-0.7	1.6	0.00834#	17	1.17	2
PE(14:0/18:1)	$C_{37}H_{72}NO_8P$	689.4996	7.3	LC-MS ESI(-)	2	-0.9	2.2	0.00024#	28	1.28	2
PE(P-18:1/16:0)	$C_{39}H_{76}NO_7P$	701.5359	12.8	LC-MS ESI(-)	0	-0.8	1.5	0.00724#	16	1.16	2
PE(20:4/P-18:1)	$C_{43}H_{76}NO_7P$	749.5359	11.4	LC-MS ESI(-)	0	-0.8	3.0	0.00004#	47	1.47	6
PS(18:0/22:5)	$C_{46}H_{80}NO_{10}P$	837.552	13.7	LC-MS ESI(-)	1	-0.4	1.1	0.03386	10	1.10	11
PS(18:0/18:1)	$C_{42}H_{80}NO_{10}P$	789.552	14.2	LC-MS ESI(-)	5	-0.8	1.2	0.00184#	8	1.08	1
Phosphocholine	$C_5H_{15}NO_4P$	184.0739	1.7	LC-MS ESI(+)	1	-0.5	1.4	0.03945	32	1.32	7
Acetyl-mannosamine/ glucosamine	$C_8H_{15}NO_6$	221.0899	19.0	GC-MS	n.a.	0.6	1.8	0.01839	31	1.31	12
Isovalerylcarnitine^{STD}	$C_{12}H_{23}NO_4$	245.1627	9.9	CE-MS	1	0.4	1.7	n/s	30	1.30	9
L-Palmitoylcarnitine^{STD}	$C_{23}H_{45}NO_4$	399.3349	1.0	LC-MS ESI(+)	0	0.6	1.3	n/s	-17	-1.21	4
Creatinine^{STD}	$C_4H_7N_3O$	113.0589	8.2	CE-MS	9	0.4	1.3	n/s	18	1.18	6
Creatine^{STD}	$C_4H_9N_3O_2$	131.0695	9.2	CE-MS	4	0.4	1.1	n/s	12	1.12	3
Citric acid	$C_6H_8O_7$	192.027	16.4	GC-MS	n.a.	-0.5	1.4	n/s	-21	-1.27	3
3-Hydroxybutyric acid^{STD}	$C_4H_8O_3$	104.0473	8.2	GC-MS	n.a.	-0.4	2.3	n/s	-39	-1.63	2
Xanthine	$C_5H_4N_4O_2$	152.033	18.5	GC-MS	n.a.	-0.4	1.3	n/s	-17	-1.20	14
DG(32:0)	$C_{35}H_{68}O_5$	568.5067	14.7	LC-MS ESI(+)	7	0.9	1.4	n/s	-24	-1.31	6
DG(34:1)	$C_{37}H_{70}O_5$	594.5223	15.9	LC-MS ESI(+)	1	0.9	1.4	n/s	-22	-1.28	6
DG(34:3)	$C_{37}H_{66}O_5$	590.491	11.2	LC-MS ESI(+)	3	0.9	1.3	n/s	-28	-1.39	15
DG(36:1)	$C_{39}H_{74}O_5$	622.5536	19.2	LC-MS ESI(+)	8	0.6	1.1	n/s	-20	-1.24	20
DG(36:4)	$C_{39}H_{68}O_5$	616.5067	12.4	LC-MS ESI(+)	2	0.8	1.3	n/s	-34	-1.51	18
DG(38:5)	$C_{41}H_{70}O_5$	642.5223	14.7	LC-MS ESI(+)	17	0.6	1.4	n/s	-18	-1.21	5
DG(40:7)	$C_{43}H_{70}O_5$	666.5223	14.2	LC-MS ESI(+)	0	0.5	1.3	n/s	-19	-1.23	4
DG(40:8)	$C_{43}H_{68}O_5$	664.5067	11.8	LC-MS ESI(+)	9	0.5	1.3	n/s	-20	-1.25	4
TG(38:1)	$C_{41}H_{76}O_6$	664.5642	22.2	LC-MS ESI(+)	4	0.8	1.1	n/s	-38	-1.62	6
TG(44:1)	$C_{47}H_{88}O_6$	748.6581	29.0	LC-MS ESI(+)	2	0.9	1.3	n/s	-25	-1.34	1
TG(44:2)	$C_{47}H_{86}O_6$	746.6424	27.7	LC-MS ESI(+)	7	0.9	1.5	n/s	-42	-1.72	4
TG(46:1)	$C_{49}H_{92}O_6$	776.6894	30.9	LC-MS ESI(+)	1	0.8	1.0	n/s	-12	-1.14	9

**Compound ID**	**Formula**	**Mass**	**MT /RT**	**Platform**	**Error**	**pcorr**	**VIP**	***P*-value**	**% Change**	**FC**	**CV QC**
TG(46:2)	$C_{49}H_{90}O_6$	774.6737	29.7	LC-MS ESI(+)	2	0.9	1.3	n/s	-23	-1.30	6
TG(48:2)	$C_{51}H_{94}O_6$	802.705	31.5	LC-MS ESI(+)	1	0.9	1.0	n/s	-14	-1.16	6
TG(48:3)	$C_{51}H_{92}O_6$	800.6894	30.3	LC-MS ESI(+)	1	0.8	1.2	n/s	-20	-1.25	9
TG(49:1)	$C_{52}H_{98}O_6$	818.7363	33.4	LC-MS ESI(+)	1	0.9	1.2	n/s	-25	-1.33	8
TG(50:3)	$C_{53}H_{96}O_6$	828.7207	32.0	LC-MS ESI(+)	1	0.9	1.2	n/s	-24	-1.32	5
TG(50:4)	$C_{53}H_{94}O_6$	826.705	30.9	LC-MS ESI(+)	1	0.9	1.1	n/s	-19	-1.24	9
TG(51:3)	$C_{54}H_{98}O_6$	842.7363	32.8	LC-MS ESI(+)	1	0.9	1.3	n/s	-26	-1.36	9
TG(52:2)	$C_{55}H_{102}O_6$	858.7676	34.9	LC-MS ESI(+)	0	0.9	1.4	n/s	-32	-1.47	13
TG(52:3)	$C_{55}H_{100}O_6$	856.752	33.7	LC-MS ESI(+)	1	0.9	1.4	n/s	-32	-1.48	10
TG(53:3)	$C_{56}H_{102}O_6$	870.7676	34.6	LC-MS ESI(+)	0	0.9	1.5	n/s	-34	-1.52	12
TG(53:4)	$C_{56}H_{100}O_6$	868.752	33.4	LC-MS ESI(+)	1	0.9	1.4	n/s	-31	-1.45	8
TG(54:2)	$C_{57}H_{106}O_6$	886.7989	37.2	LC-MS ESI(+)	0	0.8	1.2	n/s	-26	-1.34	11
TG(54:4)	$C_{57}H_{102}O_6$	882.7676	34.3	LC-MS ESI(+)	0	0.9	1.6	n/s	-37	-1.60	13
TG(54:5)	$C_{57}H_{100}O_6$	880.752	33.1	LC-MS ESI(+)	1	0.9	1.3	n/s	-24	-1.32	8
PC(32:0)	$C_{40}H_{80}NO_8P$	733.5622	9.6	LC-MS ESI(+)	0	0.6	1.1	n/s	-10	-1.11	6
PC(40:4)	$C_{48}H_{88}NO_8P$	837.6248	14.7	LC-MS ESI(+)	2	0.7	1.2	n/s	-14	-1.17	12
PC(40:5)	$C_{48}H_{86}NO_8P$	835.6091	12.2	LC-MS ESI(+)	5	0.7	1.1	n/s	-12	-1.14	4
Hypotaurine	$C_2H_7NO_2S$	109.0198	14.0	GC-MS	n.a.	-0.4	1.2	n/s	-16	-1.19	5
Pentose (ribose)	$C_5H_{10}O_5$	150.0528	14.9	GC-MS	n.a.	-0.5	1.3	n/s	-17	-1.20	8
Trisaccharide (maltotriose)	$C_{18}H_{32}O_{16}$	504.169	30.4	GC-MS	n.a.	0.5	1.3	n/s	29	1.29	26

Compounds significantly altered in response to (*R*,*S′*)-MNF, as per univariate analyses and VIP-p(corr) scores (gray background), were depicted on Figure 4A in the main text. Remaining metabolites were identified based only on the VIP-p(corr) values, thus need to be interpreted with caution as applied OPLS-DA models are prone to over-fitting.
Mass, monoisotopic molecular weight in Da; RT, Retention Time; MT, Migration Time; Error, mass error in PPM; p(corr), multivariate correlation coefficient; VIP, Variable Influence on the Projection; % of change expressed in (*R*,*S′*)-MNF group; FC, Fold Change; CV, coefficient of variation calculated for QC samples; ^{STD}, compounds confirmed by standards; #, FDR *P*-value; n/s, not significant; n.a., not available.

Table S3. Compounds quantified by targeted metabolomics.

Compound ID	concentration in vehicle controls (nM)	concentration in (*R*,*S′*)-MNF treatment group (nM)	*P*-value (t-test)	Significance indicator
2-Aminobutyric acid	2.784 ± 0.410	5.128 ± 3.208	0.03454	*
Trans-4-hydroxyproline	11.003 ± 1.111	16.878 ± 2.191	1E-06	****
Uridine	44.708 ± 6.377	56.500 ± 7.404	0.00166	**
Ophthalmic acid	0.179 ± 0.061	0.602 ± 0.487	0.01413	*
Creatinine	28.641 ± 6.339	33.911 ± 5.873	0.07839	n/s
L-Carnitine	31.028 ± 2.812	36.338 ± 3.123	0.00115	**
Acetylcarnitine	11.300 ± 1.466	9.765 ± 1.660	0.04712	*
Propionylcarnitine	0.319 ± 0.076	0.403 ± 0.093	0.04258	*
Isovalerylcarnitine	0.113 ± 0.040	0.147 ± 0.036	0.07049	n/s
Creatine	365.421 ± 56.325	409.031 ± 38.255	0.06782	n/s
L-Aspartic acid	64.426 ± 9.783	78.763 ± 13.823	0.01749	*
Uracil	38.412 ± 2.578	42.335 ± 2.979	0.00682	**
Adenosine	163.318 ± 19.767	222.029 ± 35.175	0.00028	***
3-HB	0.594 ± 0.319	0.365 ± 0.349	0.15322	n/s
Alanine	0.573 ± 0.202	0.764 ± 0.190	0.04971	*
Isoleucine	7.405 ± 0.843	6.475 ± 1.087	0.05134	*
Orotic acid	0.082 ± 0.018	0.052 ± 0.017	0.00192	**
Linoleic acid	3.092 ± 0.701	2.405 ± 0.378	0.01816	*
L-Palmitoylcarnitine	0.241 ± 0.052	0.200 ± 0.047	0.08701	n/s
Stearoylcarnitine	0.377 ± 0.122	0.253 ± 0.076	0.01798	*

The values represent the average ± SD. Control group, n = 10; (*R*,*S′*)-MNF group, n = 9. *, **, ***, ****, *P*-value < 0.05, 0.01, 0.001, 0.0001; n/s, not significant.

Table S4. Metabolite signature of PANC-1 xenograft tumor in mice treated with (*R*,*S′*)-MNF.

Compound ID	HMDB	CAS	InChI Key	FC
Ophthalmic acid	0005765	495-27-2	JCMUOFQHZLPHQP-BQBZGAKWSA-N	3.37
2-Aminobutyric acid	0000452	1492-24-6	QWCKQJZIFLGMSD-VKHMYHEASA-N	1.84
Trans-4-hydroxyproline	0000725	51-35-4	PMMYEEVYMWASQN-DMTCNVIQSA-N	1.53
Adenosine	0000050	58-61-7	OIRDTQYFTABQOQ-KQYNXXCUSA-N	1.36
beta Alanine	0000056	107-95-9	UCMIRNVEIXFBKS-UHFFFAOYSA-N	1.33
Phosphocholine	0001565	3616-044	YHHSONZFOIEMCP-UHFFFAOYSA-O	1.32
Acetylaspartylglutamate	0001067	3106-85-2	OPVPGKGADVGKTG-UHFFFAOYSA-N	1.24
Acetyl-mannosamine/glucosamine*	0000803	14131-68-1	OVRNDRQMDRJTHS-RTRLPJTCSA-N	1.31
Isovalerylcarnitine	0000688	31023-24-2	IGQBPDJNUXPEMT-UHFFFAOYSA-N	1.30
Trisaccharide (maltotriose*)	01262	1109-28-0	FYGDTMLNYKFZSV-DZOUCCHMSA-N	1.29
Propionylcarnitine	0000824	20064-19-1	UFAHZIUFPNSHSL-UHFFFAOYSA-N	1.27
Uridine	0000296	58-96-8	DRTQHJPVMGBUCF-XVFCMESISA-N	1.26
L-Aspartic acid	0000191	56-84-8	CKLJMWTZIZZHCS-REOHCLBHSA-N	1.22
Creatinine	0000562	60-27-5	DDRJAANPRJIHGJ-UHFFFAOYSA-N	1.18
L-Carnitine	0000062	541-15-1	PHIQHXFUZVPYII-ZCFIWIBFSA-N	1.17
Creatine	0000064	57-00-1	CVSVTCORWBXHQV-UHFFFAOYSA-N	1.12
Uracil	0000300	66-22-8	ISAKRJDGNUQOIC-UHFFFAOYSA-N	1.10
Isoleucine	0000172	73-32-5	AGPKZVBTJJNPAG-WHFBIAKZSA-N	-1.14
Acetylcarnitine	0000201	3040-38-8	RDHQFKQIGNGIED-UHFFFAOYSA-N	-1.16
Hypotaurine	00965	300-84-5	VVIUBCNYACGLLV-UHFFFAOYSA-N	-1.19
Pentose (ribose*)	00283	613-83-2	HMFHBZSHGGEWLO-SOOFDHNKSA-N	-1.20
Xanthine	00292	69-89-6	LRFVTYWOQMYALW-UHFFFAOYSA-N	-1.20
L-Palmitoylcarnitine	0000222	2364-67-2	XOMRRQXKHMYMOC-OAQYLSRUSA-N	-1.21
Hydroxybutyrylcarnitine	0013127	1469900-92-2	UEFRDQSMQXDWTO-GKAPJAKFSA-N	-1.25
Citric acid	00094	77-92-9	KRKNYBCHXYNGOX-UHFFFAOYSA-N	-1.27
Linoleic acid	0000673	60-33-3	OYHQOLUKZRVURQ-HZJYTTRNSA-N	-1.29
Stearoylcarnitine	0000848	25597-09-5	FNPHNLNTJNMAEE-UHFFFAOYSA-N	-1.49
Orotic acid	0000226	65-86-1	PXQPEWDEAKTCGB-UHFFFAOYSA-N	-1.56
3-Hydroxybutyric acid	0000357	625-72-9	WHBMMWSBFZVSSR-UHFFFAOYSA-N	-1.63
Methyl oleate	n.a.	112-62-9	QYDYPVFESGNLHU-KHPPLWFESA-N	-1.72

n.a., not available; *HMDB/CAS number given only for marked compound. Compounds related to the glycerolipid and glycerophospholipid class are not included as HMDB/CAS number is either not available or different HMDB numbers are assigned according to the several stereoisomers; FC, Fold Change; HMDB, Human Metabolome Database; CAS, Chemical Abstracts Service; InChI, International Chemical Identifier code.

Table S5. GO Term signature of (*R*,*S*′)-MNF treatment of PANC-1 xenograft tumor in mice.
Top 15 biological processes significantly up- and down-regulated in the (*R*,*S*′)-MNF:vehicle pairwise comparison are listed.

Gene Ontology Term	Number of genes	Zscore
GO0006695 Cholesterol Biosynthetic Process	25	5.906
GO0016126 Sterol Biosynthetic Process	21	5.264
GO0006412 Translation	253	3.907
GO0008299 Isoprenoid Biosynthetic Process	15	3.468
GO0043666 Regulation of Phosphoprotein Phosphatase	6	3.269
GO0031532 Actin Cytoskeleton Reorganization	4	2.929
GO0006801 Superoxide Metabolic Process	13	2.748
GO0015992 Proton Transport	56	2.667
GO0051258 Protein Polymerization	15	2.657
GO0006325 Establishment and/or Maintenance of Chromat. Organiz.	21	2.393
GO0045792 Negative Regulation of Cell Size	5	2.304
GO0051259 Protein Oligomerization	8	2.203
GO0000070 Mitotic Sister Chromatid Segregation	14	2.111
GO0043552 Positive Regulation of Phosphoinositide	6	2.033
GO0006000 Fructose Metabolic Process	3	2.008
GO0006916 Anti Apoptosis	133	-6.036
GO0001892 Embryonic Placenta Development	13	-5.936
GO0000122 Negative Regulation of Transcription from RNA Pol II ..	176	-5.546
GO0007001 Chromosome Organization and Biogenesis (sensu Euka.)	87	-5.492
GO0046777 Protein Amino Acid Autophosphorylation	48	-5.298
GO0008333 Endosome to Lysosome Transport	6	-5.196
GO0006915 Apoptosis	405	-5.069
GO0006355 Regulation of Transcription DNA-templated	1607	-5.050
GO0006468 Protein Amino Acid Phosphorylation	564	-5.019
GO0008150 Biological Process	1451	-4.981
GO0006950 Response to Stress	114	-4.943
GO0015031 Protein Transport	468	-4.800
GO0007267 Cell Cell Signaling	239	-4.752
GO0007155 Cell Adhesion	518	-4.735
GO0043066 Negative Regulation of Apoptosis	81	-4.719

Significance is defined as Z-score > 1.5 in either direction, false discovery rate (fdr) < 0.3 and $p < 0.05$. N = 10 mice per experimental group. The number of genes in a given GO Term is provided.

Table S6. Mitochondrial pathway signature of (*R,S′*)-MNF treatment of PANC-1 xenograft tumor in mice.

Pathway	Number of genes	Zscore	Class
Electron Transport Chain	86	6.688	Wikipathways
ETC-Complex 5-ATP Synthase F1 Complex	17	5.033	Wikipathways
Oxidative Phosphorylation	47	4.964	Wikipathways
Mitochondrial Inner Membrane	270	3.857	Mito Disease Foundation
Mitochondrial Membrane Part	114	3.789	Mito Disease Foundation
ETC-Complex 4-Cytochrome C Oxidase	16	3.764	Wikipathways
Mitochondrial Respiratory Chain	56	3.363	Mito Disease Foundation
Mitochondrial Respiratory Chain Complex 1	36	3.232	Mito Disease Foundation
NADH Dehydrogenase Complex	36	3.232	Mito Disease Foundation
ETC-Complex 1 NADH-Ubiquinone Oxidoreductase	35	3.037	Wikipathways
Fatty Acid Biosynthesis	14	2.395	Wikipathways
Ubiquinone Biosynthesis	4	1.586	Mitochondrial Pathway
Ubiquinone Biosynthesis in Humans	4	1.586	Mitochondrial Pathway
Ubiquinone Biosynthesis in Rats	4	1.586	Mitochondrial Pathway
Transmembr. Potential of Mito. Inner Membrane Up	5	1.407	IPA
Degeneration of Mitochondria Down	3	0.862	IPA
Mito. Fission of Cervical Cancer Cell Lines Up	4	0.500	IPA
Elongation of Mitochondria Up	5	-1.154	IPA
Density of Mitochondria Up	7	-1.566	IPA
Permeabilization of Mitochondria Up	32	-2.179	IPA
Transport of Mitochondria Up	14	-2.208	IPA
Depolarization of Mitochondria Up	30	-2.705	IPA
Damage of Mitochondria Up	24	-2.756	IPA
Morphology of Mitochondria Up	42	-2.879	IPA
Transmembrane Potential of Mitochondria Up	166	-3.250	IPA
Organization of Mitochondria Up	58	-3.326	IPA
Injury of Mitochondria Down	4	-3.540	IPA
Transmembrane Potential of Mitochondria Down	88	-3.826	IPA
Injury of Mitochondria Up	4	-4.749	IPA

Significance is defined as Z-score > 1.5 in either direction, false discovery rate (fdr) < 0.3 and $p < 0.05$. N = 10 mice per experimental group. The number of genes in a given GO Term is provided.

Table S7. Gene expression signature of (*R*,*S′*)-MNF treatment of PANC-1 xenograft tumor in mice.
A list of selected genes implicated in Hippo, Wnt/β-catenin, and growth factor signaling pathways is presented.

Symbol	Accession	Definition/Function	Zratio*
FAT	NM_005245.3	FAT atypical cadherin 1. Reduces tumor proliferation	+1.68
RASSF5	NM_182664.1	Tumor suppressor	-1.97
RASSF7	NM_003475.2	Tumor suppressor	+2.51
TAOK1	NM_020791.1	Involved in apoptotic body formation	-7.74
MST2	NM_006281.1	Restrict proliferation and promotes apoptosis	-1.80
MOBK1B	NM_018221.1	Control tumor growth	-2.94
TEAD4	NM_201441.1	TEA domain transcription 4	-1.81
WWTR1	NM_015472.3	Transcriptional co-activator TAZ	-5.46
MCL1	NM_021960.3	Anti-apoptotic protein	-2.76
CYR61	NM_001554.3	Promotes cell proliferation and chemotaxis	-3.03
MMP7	NM_002423.3	Allows tumor proliferation through HB-EGF release	-3.76
BIRC2	NM_001166.3	Apoptosis inhibitor	-3.06
GLI2	NM_005270.2	Potent oncogene by mediating SSH signaling	-2.98
WNT10A	NM_025216.2	Role in carcinogenesis through β-catenin/TCF complex	-3.84
FZD6	NM_003506.2	Receptor for Wnt proteins. Promotes apoptosis	+2.40
FZD9	NM_003508.2	Coupled to the β-catenin signaling pathway	-3.13
SFRP1	NM_003012.3	Inhibits TCF-dependent transcription	-3.36
LRP5	NM_002335.1	Triggers β-catenin signaling	-3.60
LRP10	NM_014045.3	Involved in signal transduction	-2.96
DKK1	NM_012242.2	Blocks canonical Wnt signaling	+3.43
LGR4	NM_018490.1	Activates the Wnt signaling pathway	-3.60
GSK3B	NM_002093.2	Kinase responsible for degrading β-catenin	-4.74
CTNNB1	NM_001904.2	Integral part of the canonical Wnt signaling pathway	-4.45
TCF12	NM_207036.1	Transcriptional regulator	-3.57
MYC	NM_002467.3	Proto-oncogene acting as transcription factor	-2.23
SLC2A1	NM_006516.1	GLUT1, required for aerobic glycolysis	-4.92
LDHA	NM_005566.3	Lactate dehydrogenase	-2.18
HBEGF	NM_001945.1	Potent growth factor that binds EGF receptor	-2.90
EGFR	NM_005228.3	Driving factor in many cancer types	-1.98
GRB2	NM_002086.3	Adaptor protein for Ras signaling pathway	-5.56
RHOQ	NM_012249.2	Promote reorganization of the actin cytoskeleton	-4.26
RHOB	NM_004040.2	Negative role in tumor formation	-5.57
HIF1A	NM_181054.1	Master transcriptional regulator in response to hypoxia	-1.94
HSPA1A	NM_005345.4	Hsp70, a buffering system for cancer cell survival	-5.46
ABCC5	NM_001023587.1	Involved in multidrug resistance	-4.25
PFKFB4	NM_004567.2	Involved in glucose metabolism and induced by hypoxia	-3.83
ITGA2	NM_002203.2	Mediates cell adhesion to the extracellular matrix	-2.85
ITGA3	NM_002204.1	Mediates cell adhesion to the extracellular matrix	-3.19
ITGA5	NM_002205.2	Mediates cell adhesion to the extracellular matrix	-2.63
ITGA6	NM_000210.1	Mediates cell adhesion to the extracellular matrix	-4.73
EDN1	NM_001955.2	Aberrant expression may promote tumorigenesis	-5.20

* The z-ratios of significantly up (+)- and down (-)-regulated genes in the (*R*,*S′*)-MNF:Vehicle pairwise comparison are shown. Significance is defined as z-ratio > 1.5 in either direction, false discovery rate (fdr) < 0.3 and $p < 0.05$. N = 10 mice per experimental group.

Full-size blots

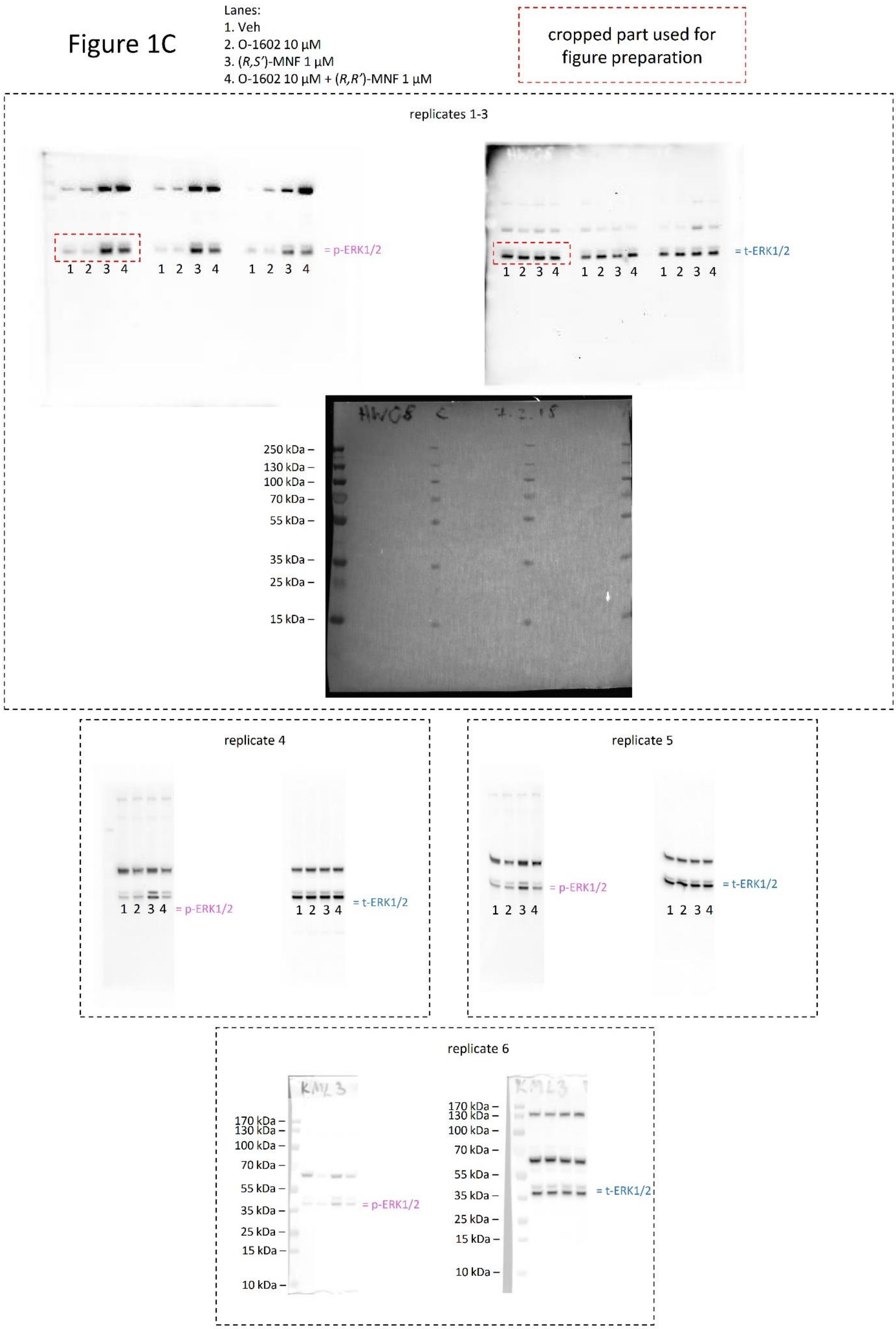

Figure 2A

Lanes:
1. Veh
2. (*R,R'*)-MNF 5 μM
3. (*R,R'*)-MNF 10 μM
4. (*R,R'*)-MNF 20 μM
5. (*R,S'*)-MNF 5 μM
6. (*R,S'*)-MNF 10 μM
7. (*R,S'*)-MNF 20 μM

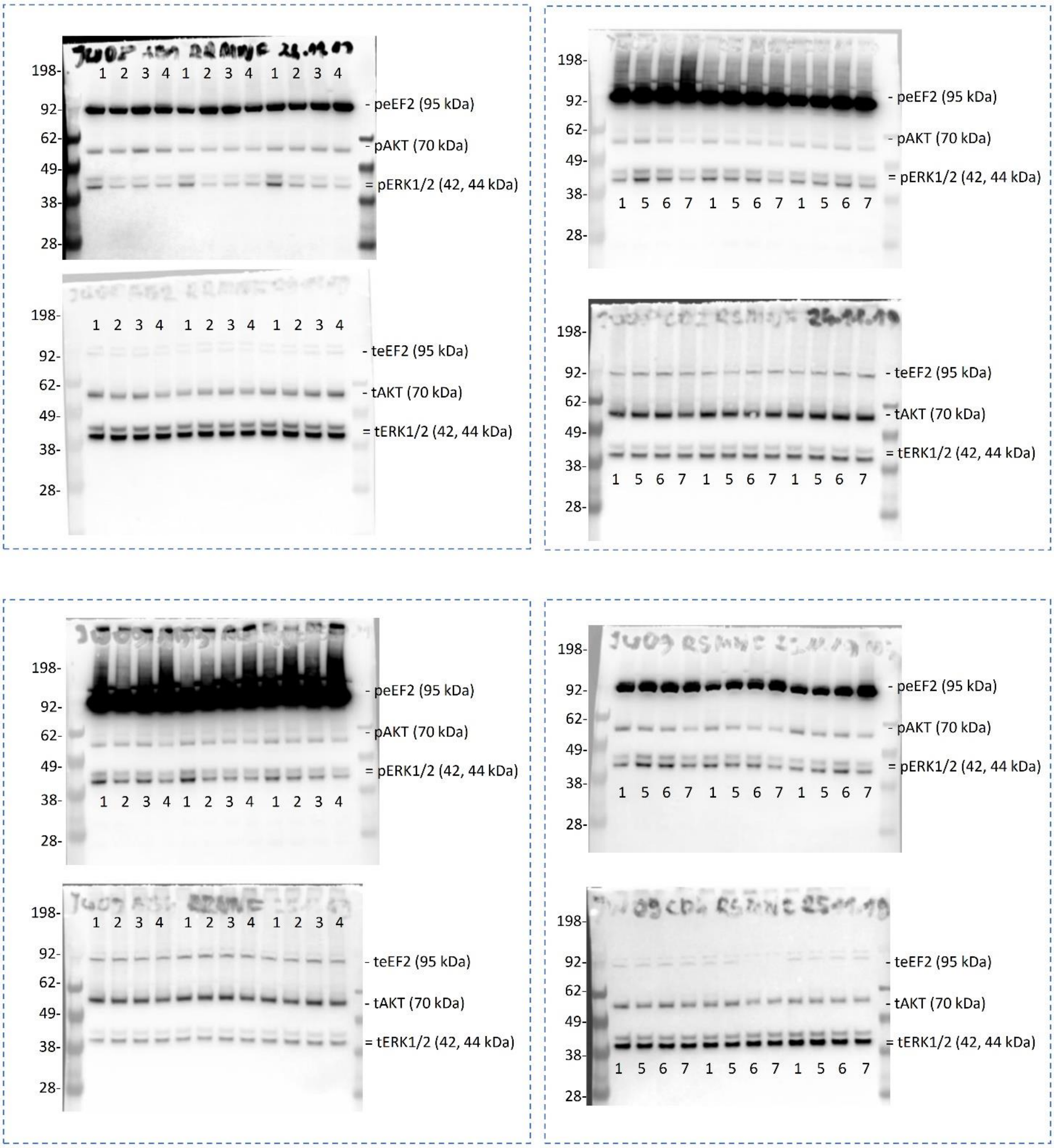

Figure 2B

Lanes:
1. Veh
2. (*R,R'*)-MNF
3. ICI
4. ICI + (*R,R'*)-MNF
5. (*R,S'*)-MNF
6. ICI + (*R,S'*)-MNF

cropped part used for figure preparation

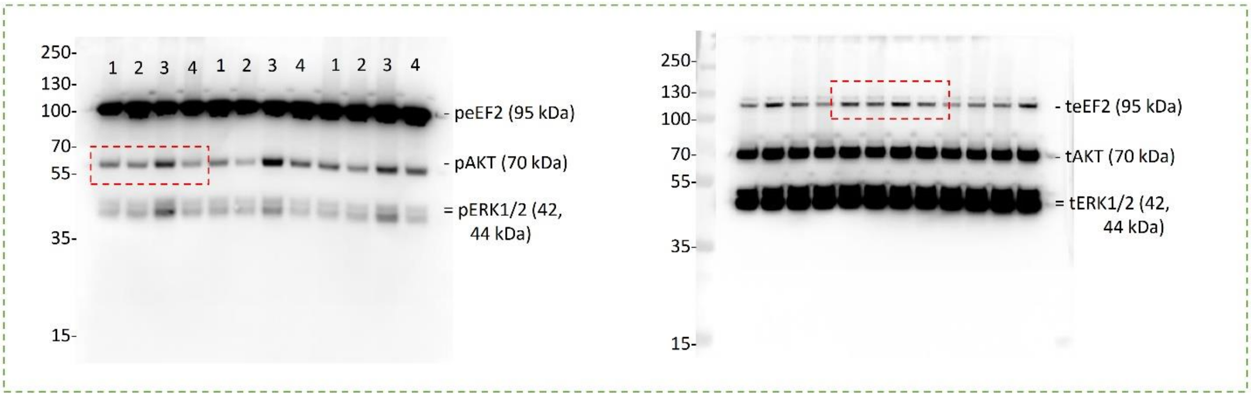

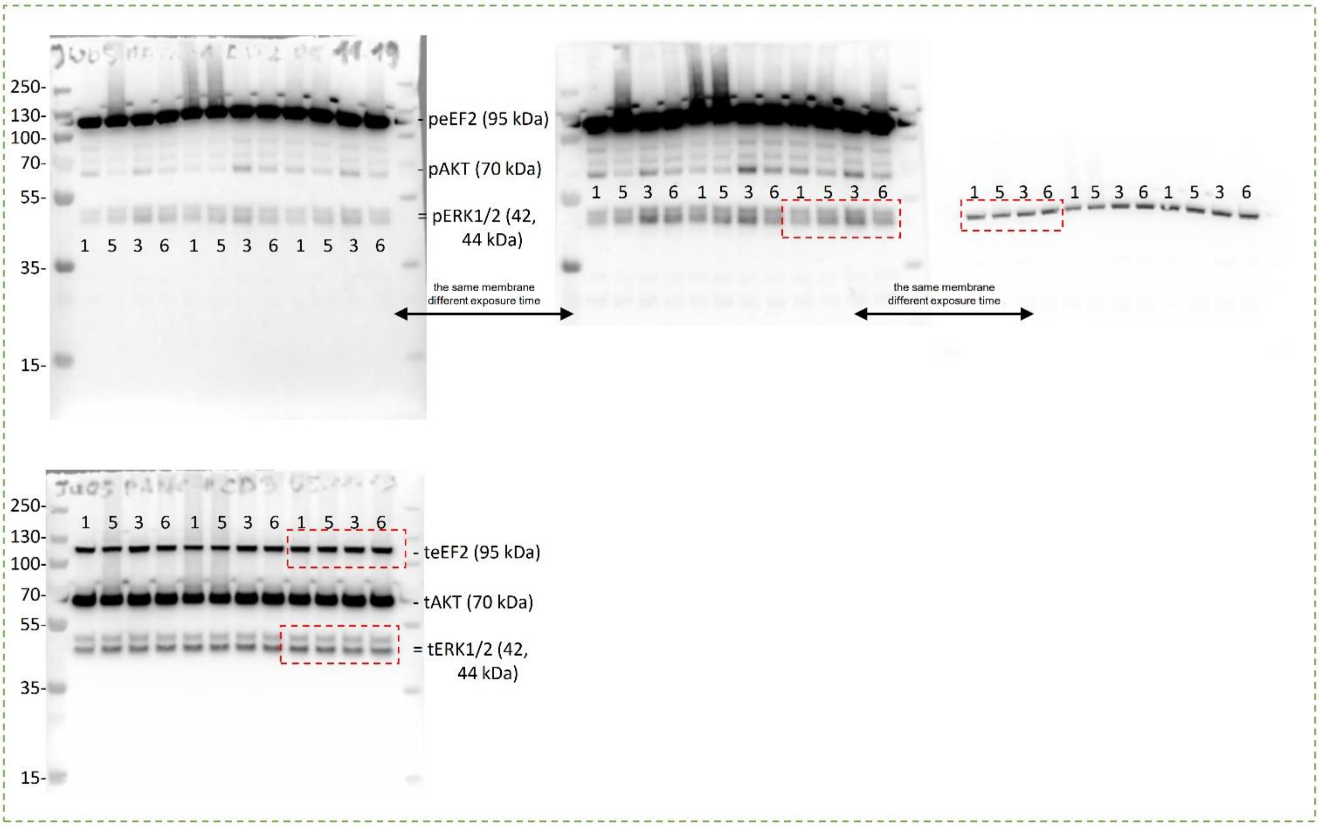

Figure 2B

Lanes:
1. Veh
2. (*R,R'*)-MNF
3. ICI
4. ICI + (*R,R'*)-MNF
5. (*R,S'*)-MNF
6. ICI + (*R,S'*)-MNF

cropped part used for figure preparation

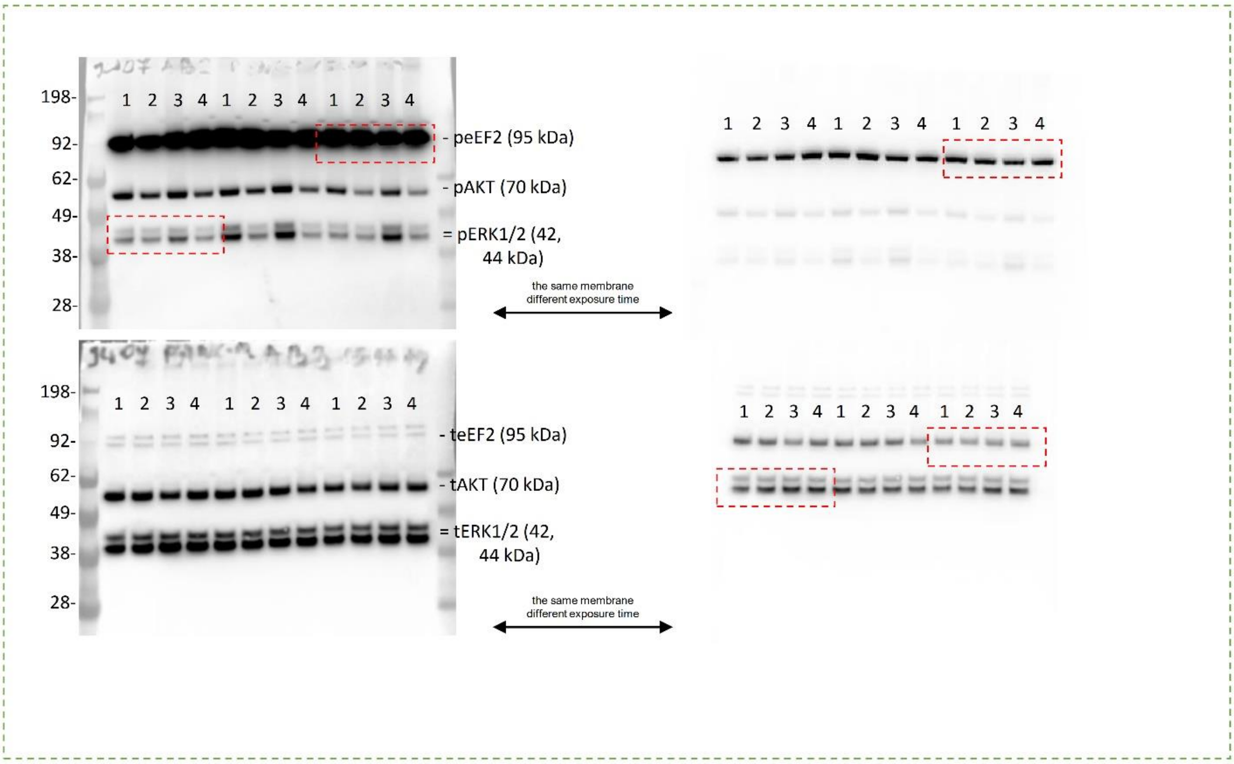

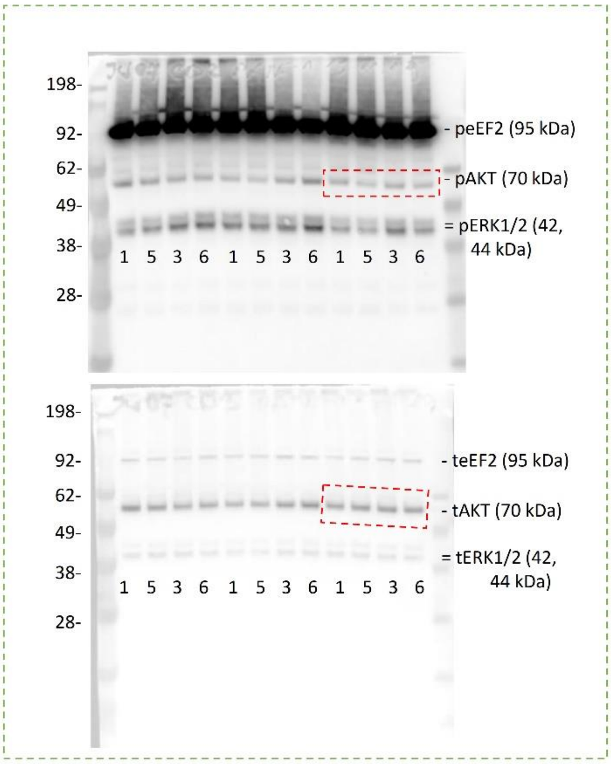

Figure 2B

Lanes:
1. Veh
2. (*R,S'*)-MNF
3. ICI
4. ICI + (*R,S'*)-MNF

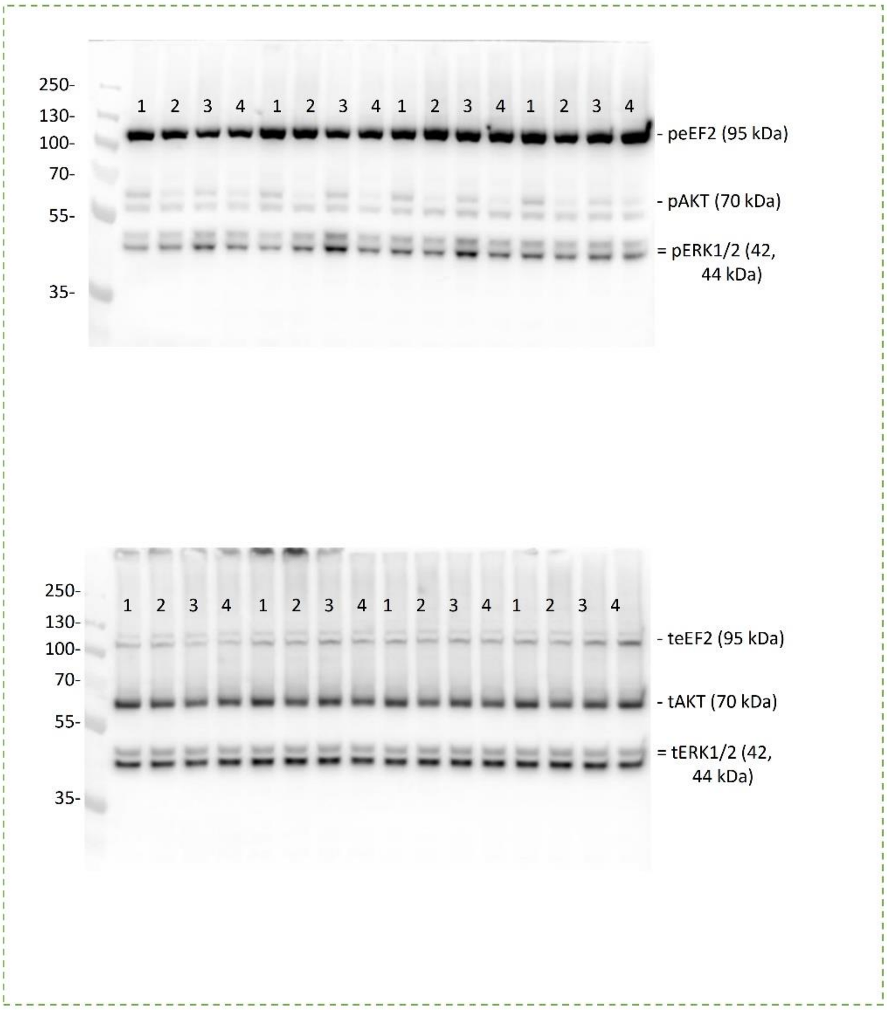

Figure 2B

Lanes:
1. Veh
2. (*R,S'*)-MNF
3. ICI
4. ICI + (*R,S'*)-MNF

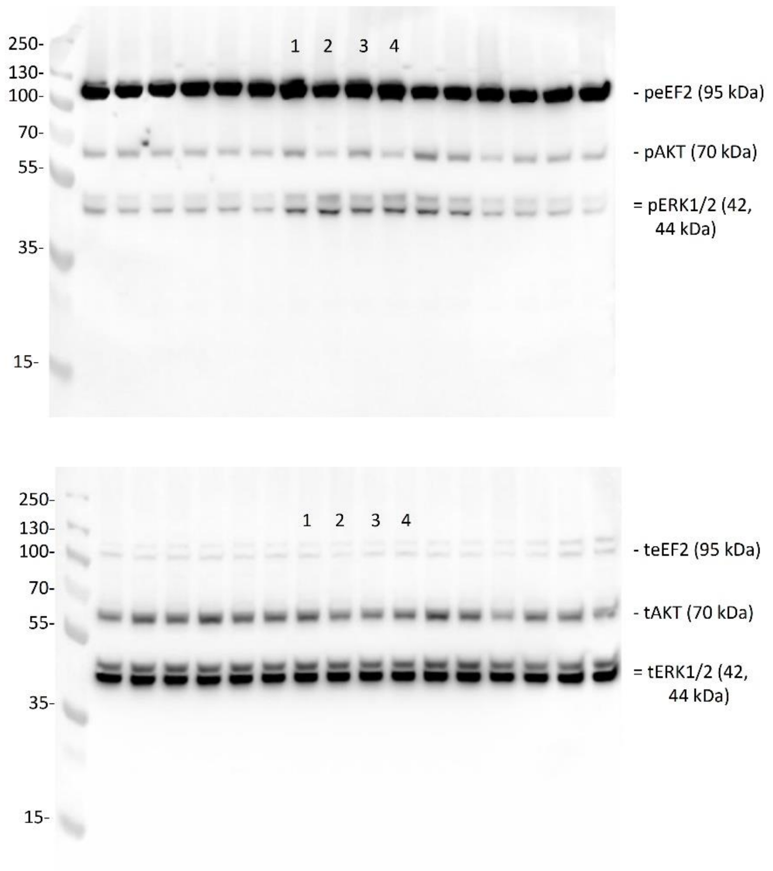

Figure 2B

Lanes:
1. Veh
2. (*R,S'*)-MNF
3. ICI
4. ICI + (*R,S'*)-MNF

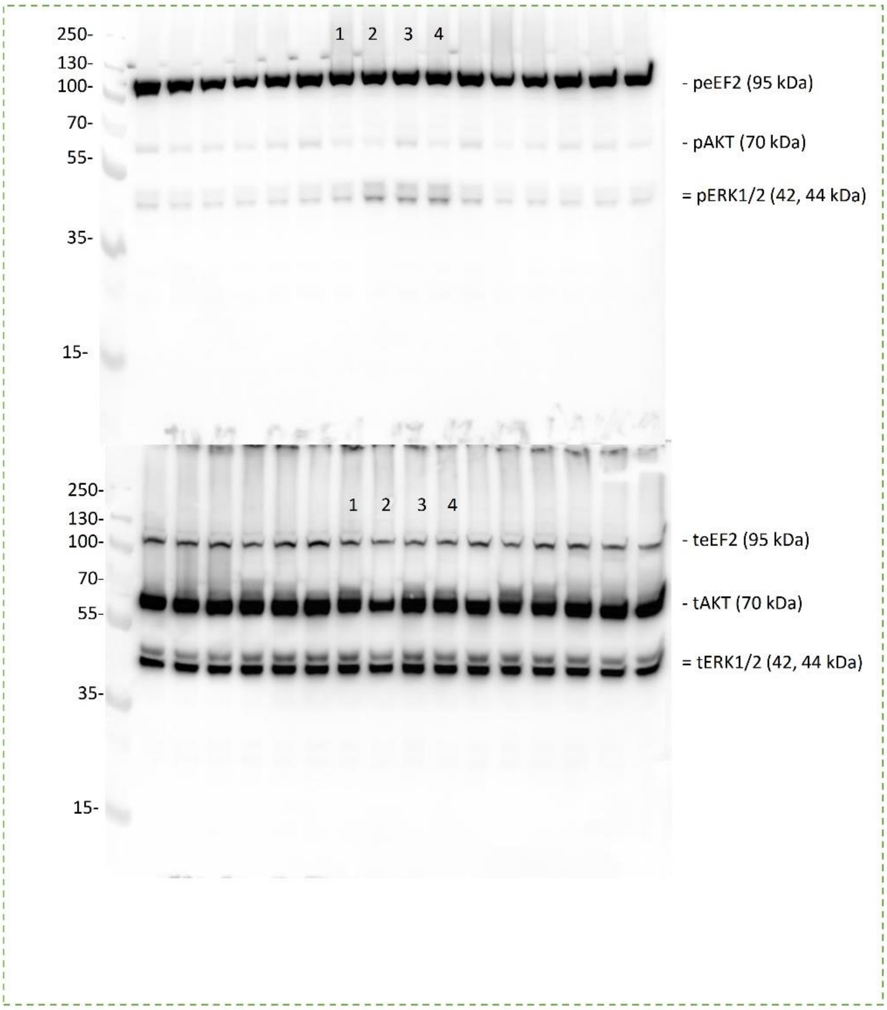

Figure 2D

1. Veh
2. (*R,R'*)-MNF 1 µM
3. ICI 50 nM
4. ICI 50 nM+ (*R,R'*)-MNF 1 µM
5. (*R,S'*)-MNF 1 µM
6. ICI 50 nM+ (*R,S'*)-MNF 1 µM

cropped part used for figure preparation

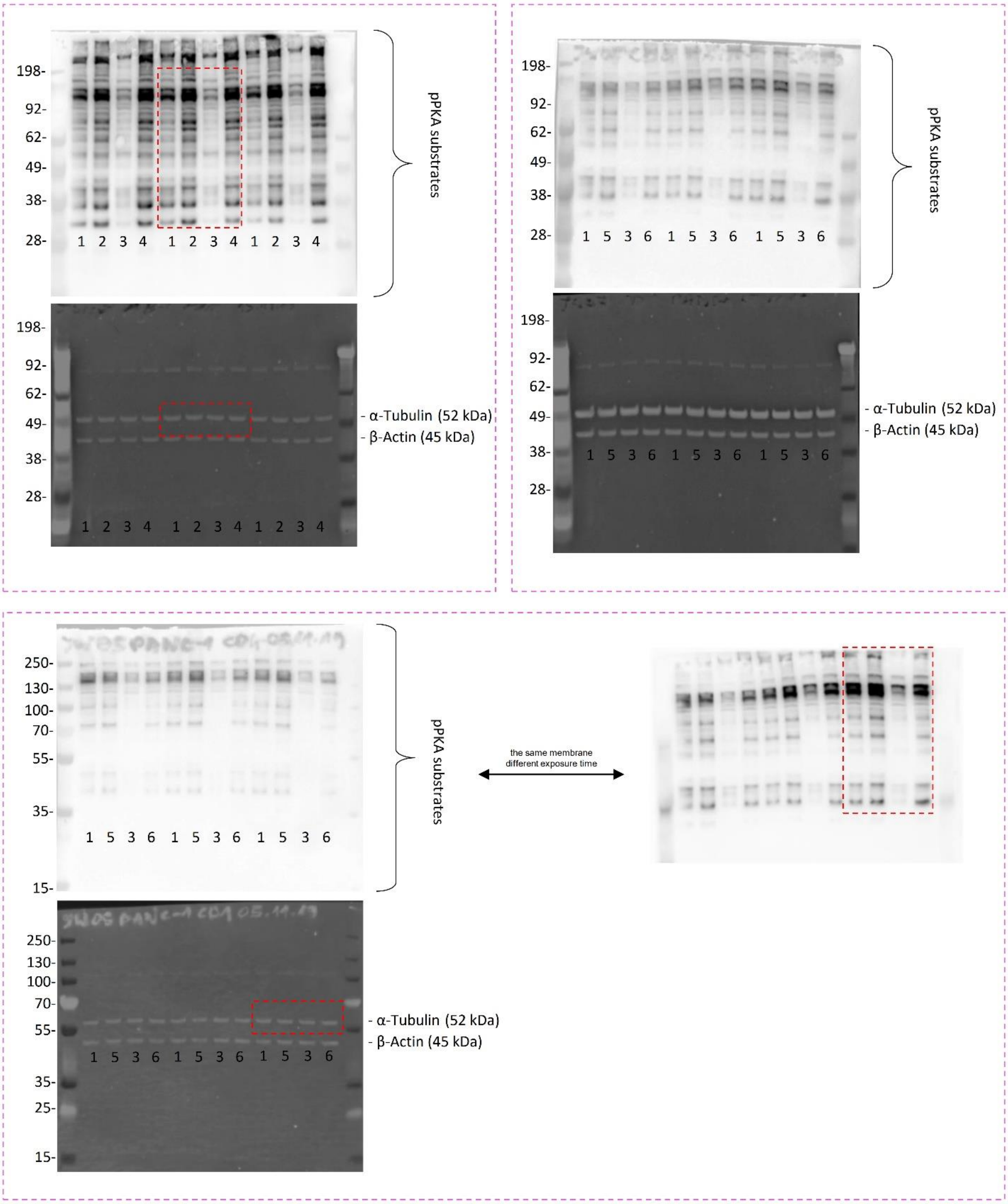

Figure 2D

1. Veh
2. (*R,R'*)-MNF 1 µM
3. ICI 50 nM
4. ICI 50 nM+ (*R,R'*)-MNF 1 µM
5. (*R,S'*)-MNF 1 µM
6. ICI 50 nM+ (*R,S'*)-MNF 1 µM

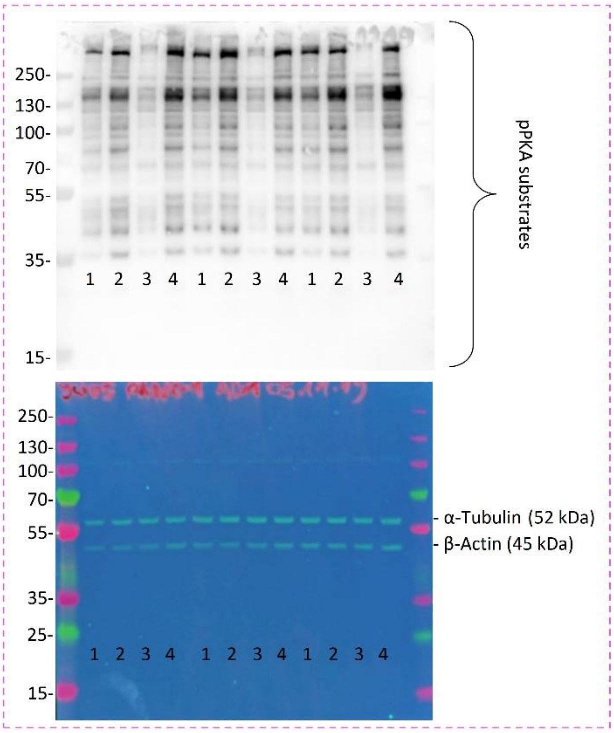

Figure 2E

Lanes:
1. Veh
2. (*R,R'*)-MNF
3. PKI
4. PKI + (*R,R'*)-MNF

cropped part used for figure preparation

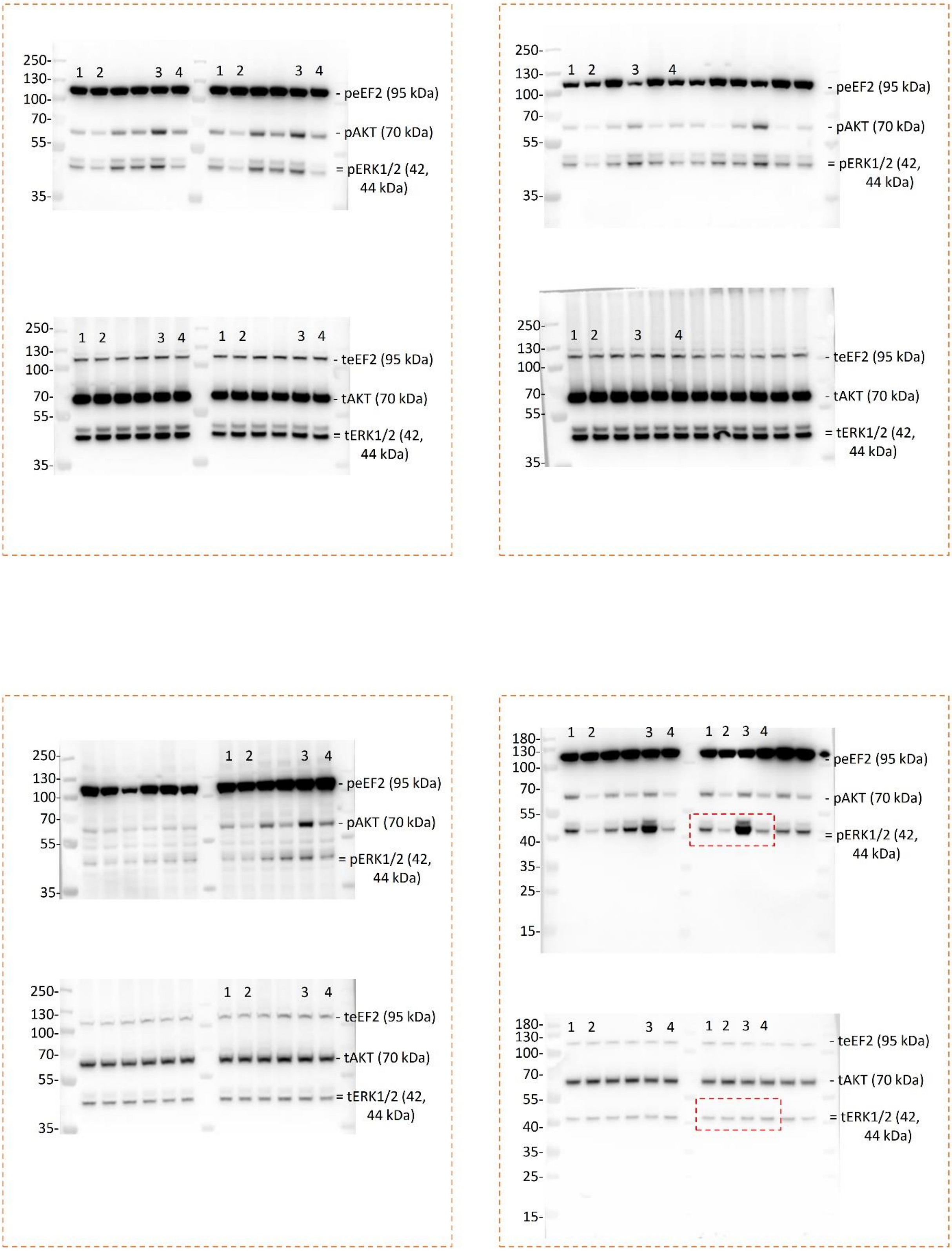

Figure 2E

Lanes:
1. Veh
2. (*R,R'*)-MNF
3. PKI
4. PKI + (*R,R'*)-MNF
5. (*R,S'*)-MNF
6. PKI + (*R,S'*)-MNF

cropped part used for figure preparation

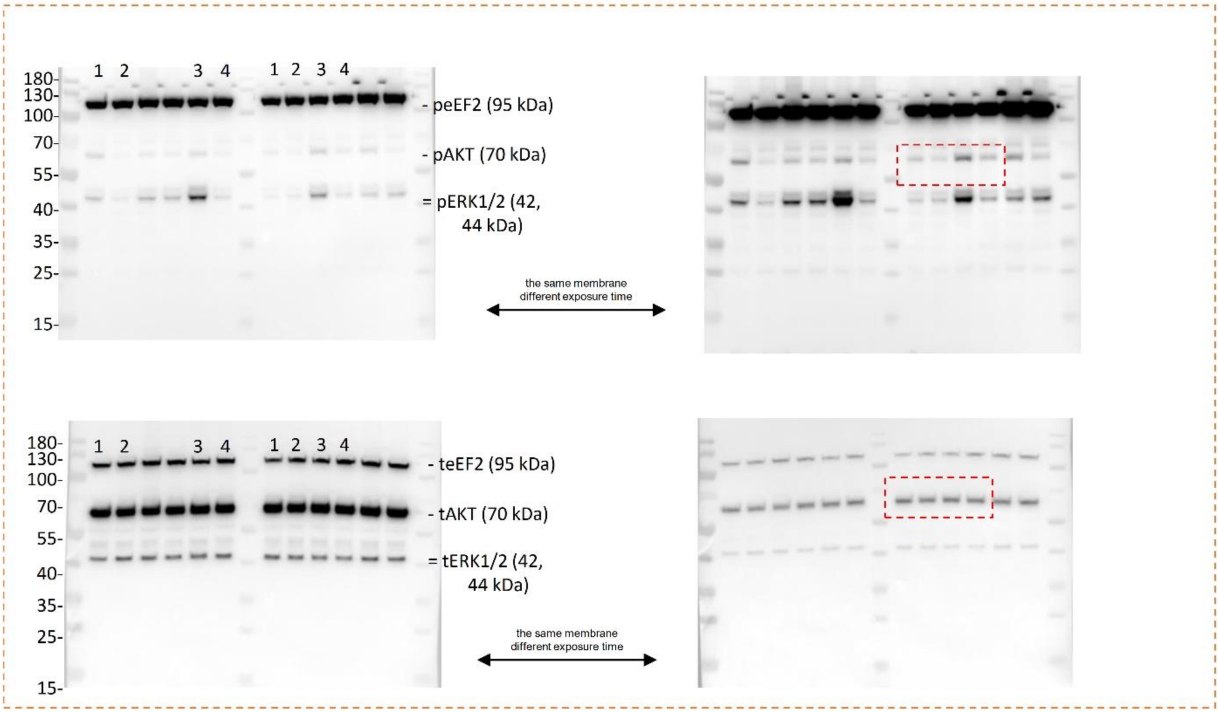

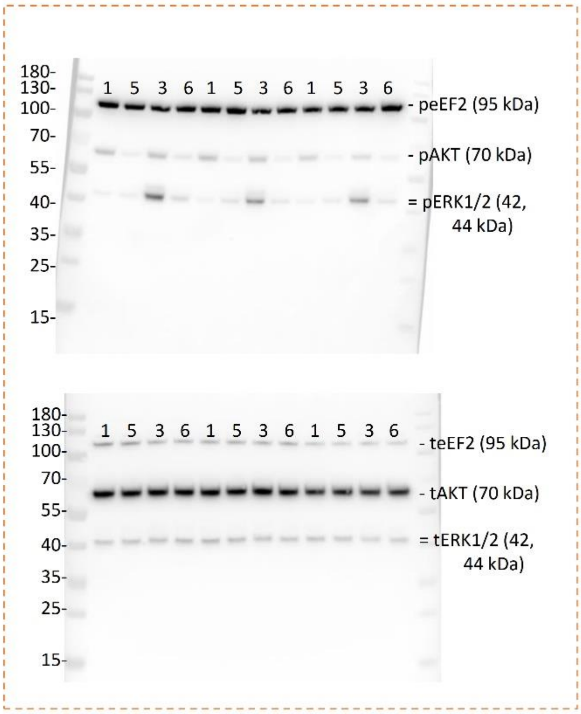

Figure 2E

Lanes:
1. Veh
2. (*R,R'*)-MNF
3. PKI
4. PKI + (*R,R'*)-MNF
5. (*R,S'*)-MNF
6. PKI + (*R,S'*)-MNF

cropped part used for figure preparation

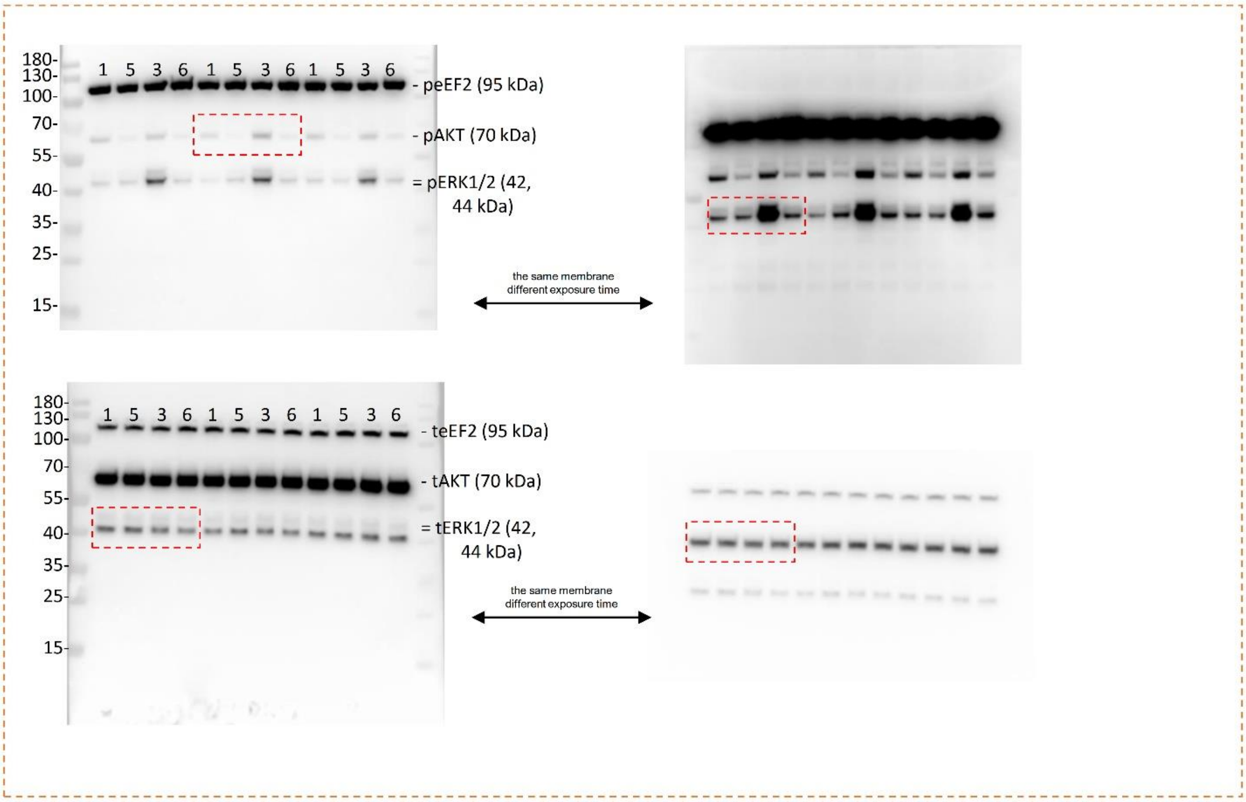

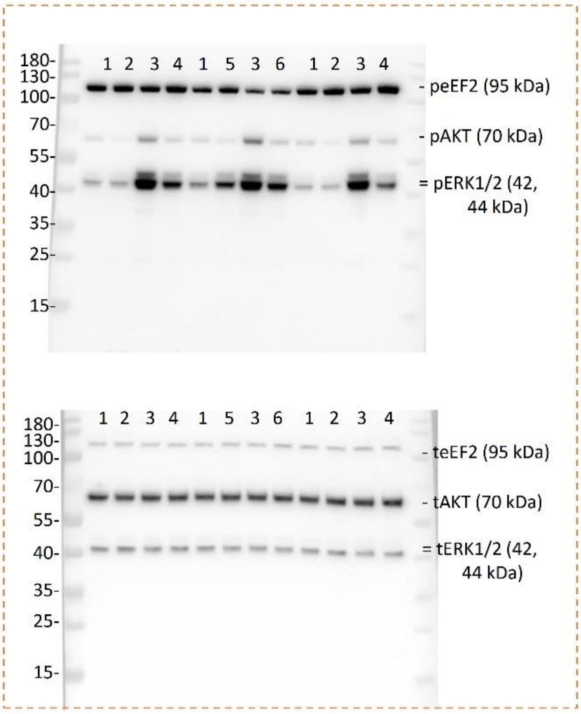

Figure 2E

Lanes:
1. Veh
2. (*R,R′*)-MNF
3. PKI
4. PKI + (*R,R′*)-MNF
5. *(R,S′)*-MNF
6. PKI + (*R,S′*)-MNF

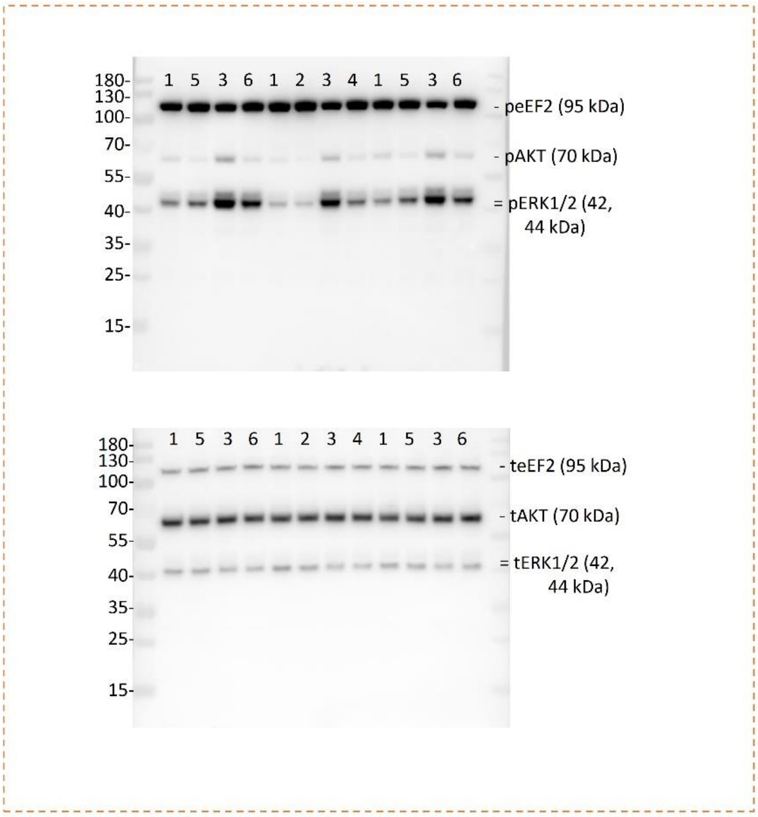

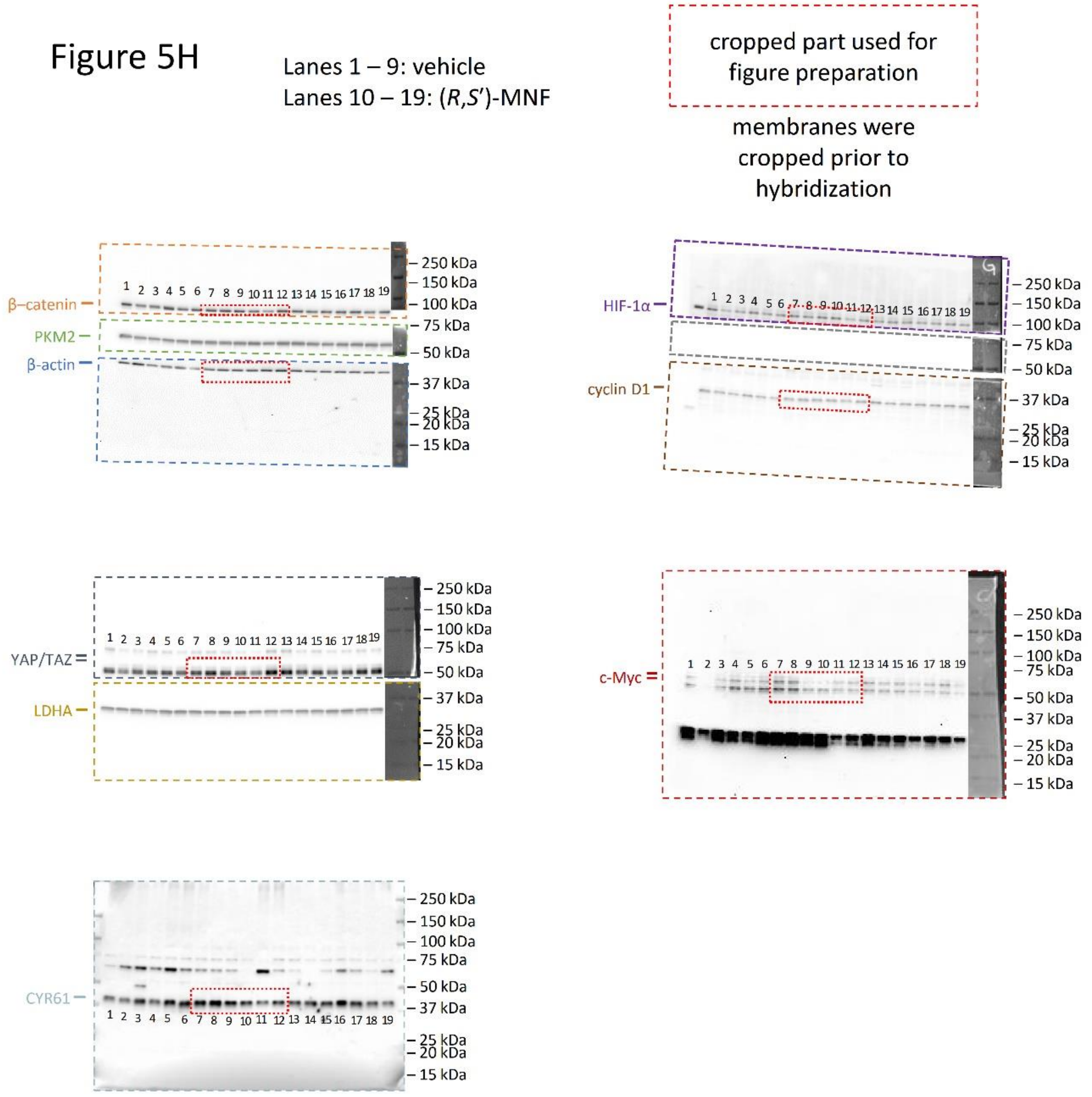
Figure 5H
Lanes 1 – 9: vehicle
Lanes 10 – 19: (R,S′)-MNF
cropped part used for figure preparation
membranes were cropped prior to hybridization
β–catenin
PKM2
β-actin
HIF-1α
cyclin D1
YAP/TAZ
LDHA
c-Myc
CYR61
250 kDa
150 kDa
100 kDa
75 kDa
50 kDa
37 kDa
25 kDa
20 kDa
15 kDa

Figure S2A

Lanes:
1. Veh
2. (*R,R'*)-Fenoterol 100 μM
3. (*R,R'*)-Fenoterol 31,6 μM
4. (*R,R'*)-Fenoterol 10 μM
5. (*R,R'*)-Fenoterol 3,16 μM
6. (*R,R'*)-Fenoterol 1 μM

cropped part used for figure preparation

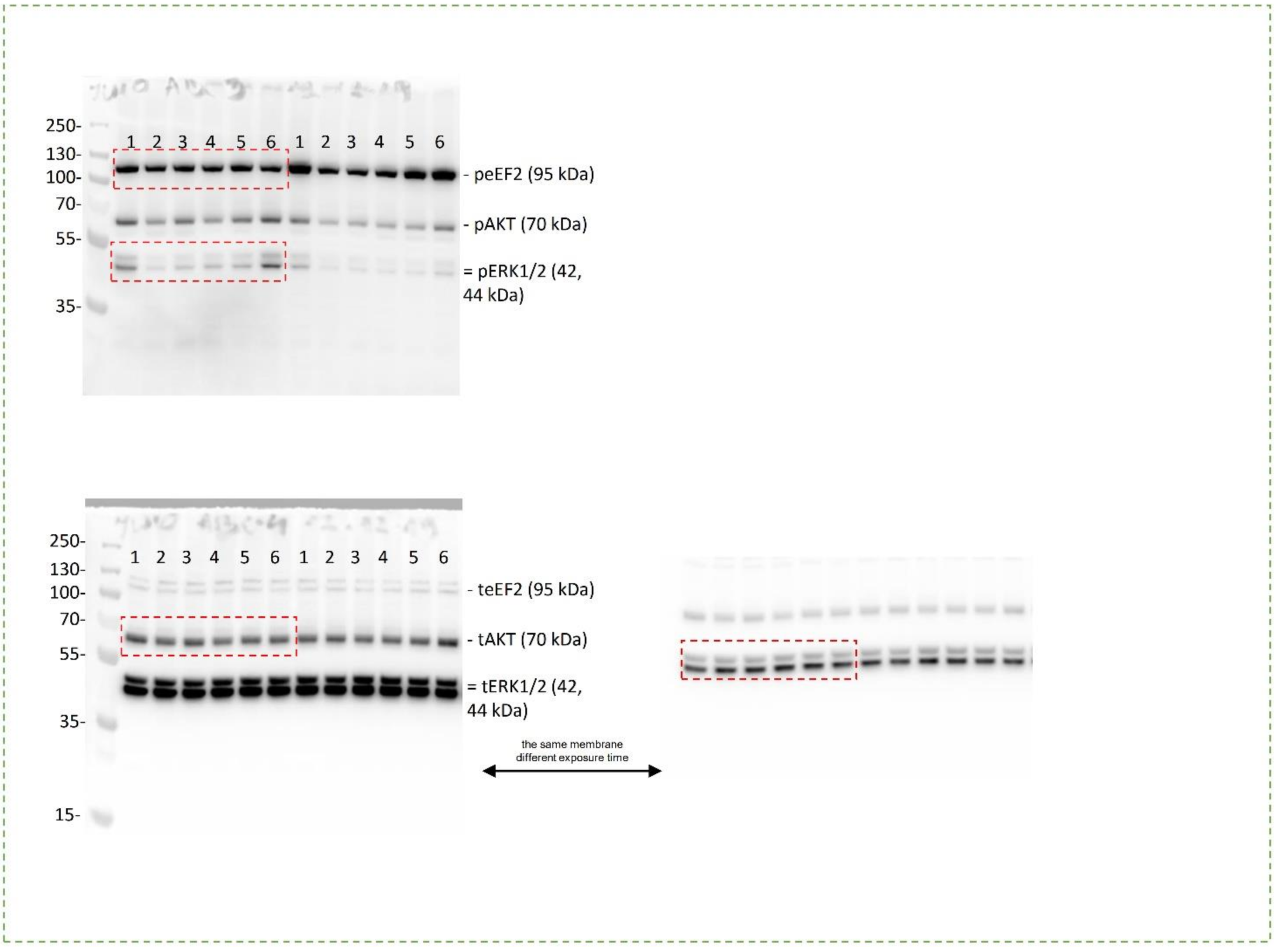

Figure S2A

Lanes:
1. Veh
2. (*R,R'*)-Fenoterol 100 μM
3. (*R,R'*)-Fenoterol 31,6 μM
4. (*R,R'*)-Fenoterol 10 μM
5. (*R,R'*)-Fenoterol 3,16 μM
6. (*R,R'*)-Fenoterol 1 μM

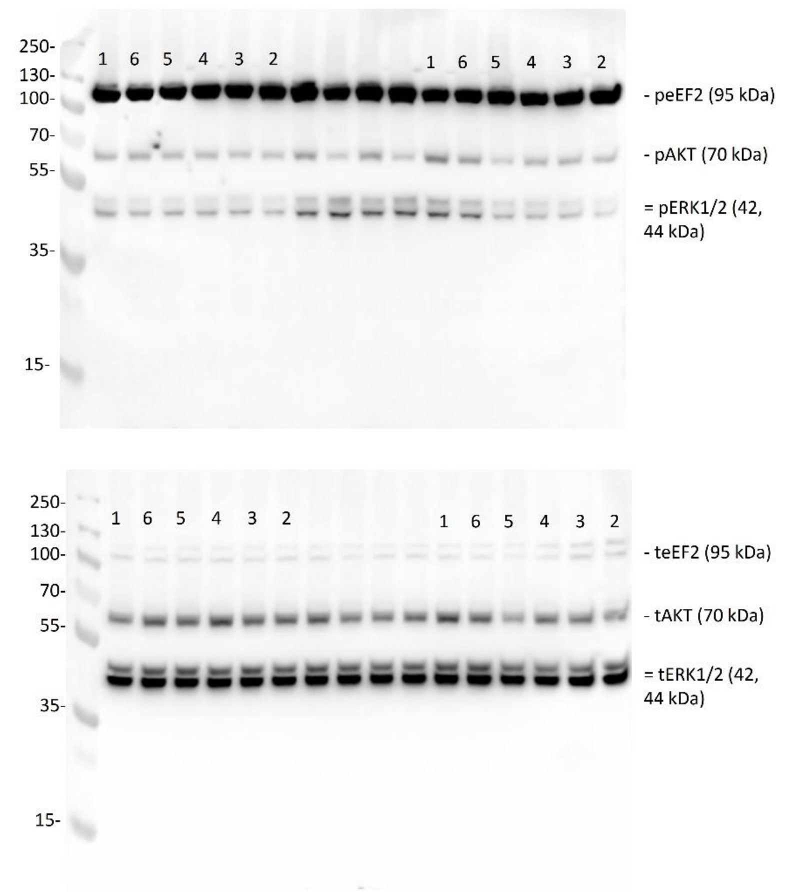

Figure S2A

Lanes:
1. Veh
2. (*R,R'*)-Fenoterol 100 µM
3. (*R,R'*)-Fenoterol 31,6 µM
4. (*R,R'*)-Fenoterol 10 µM
5. (*R,R'*)-Fenoterol 3,16 µM
6. (*R,R'*)-Fenoterol 1 µM

cropped part used for figure preparation

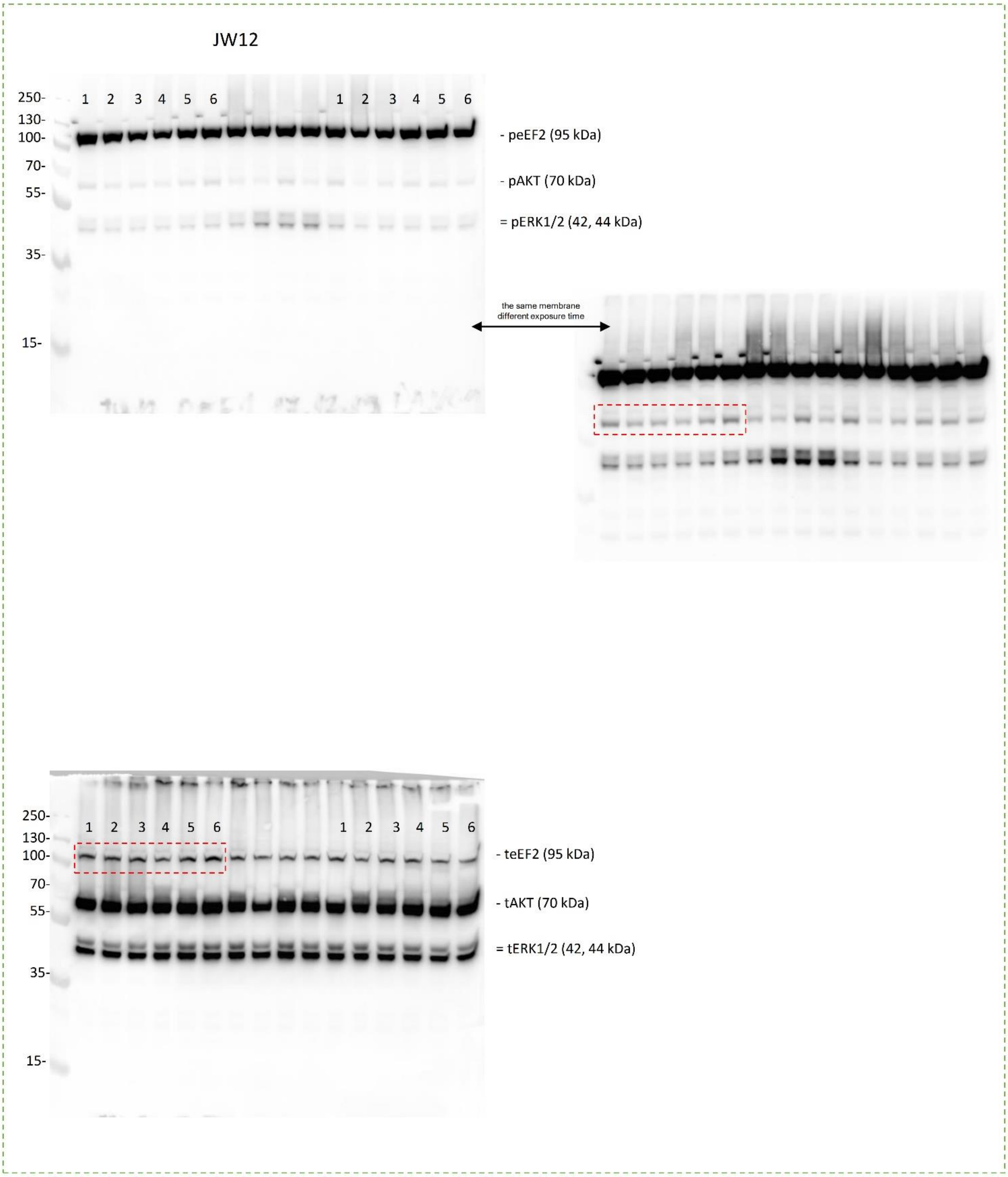

Figure S2C

1. Veh
2. Salmeterol 100 µM
3. Salmeterol 31,6 µM
4. Salmeterol 10 µM
5. Salmeterol 3,16 µM
6. Salmeterol 1 µM

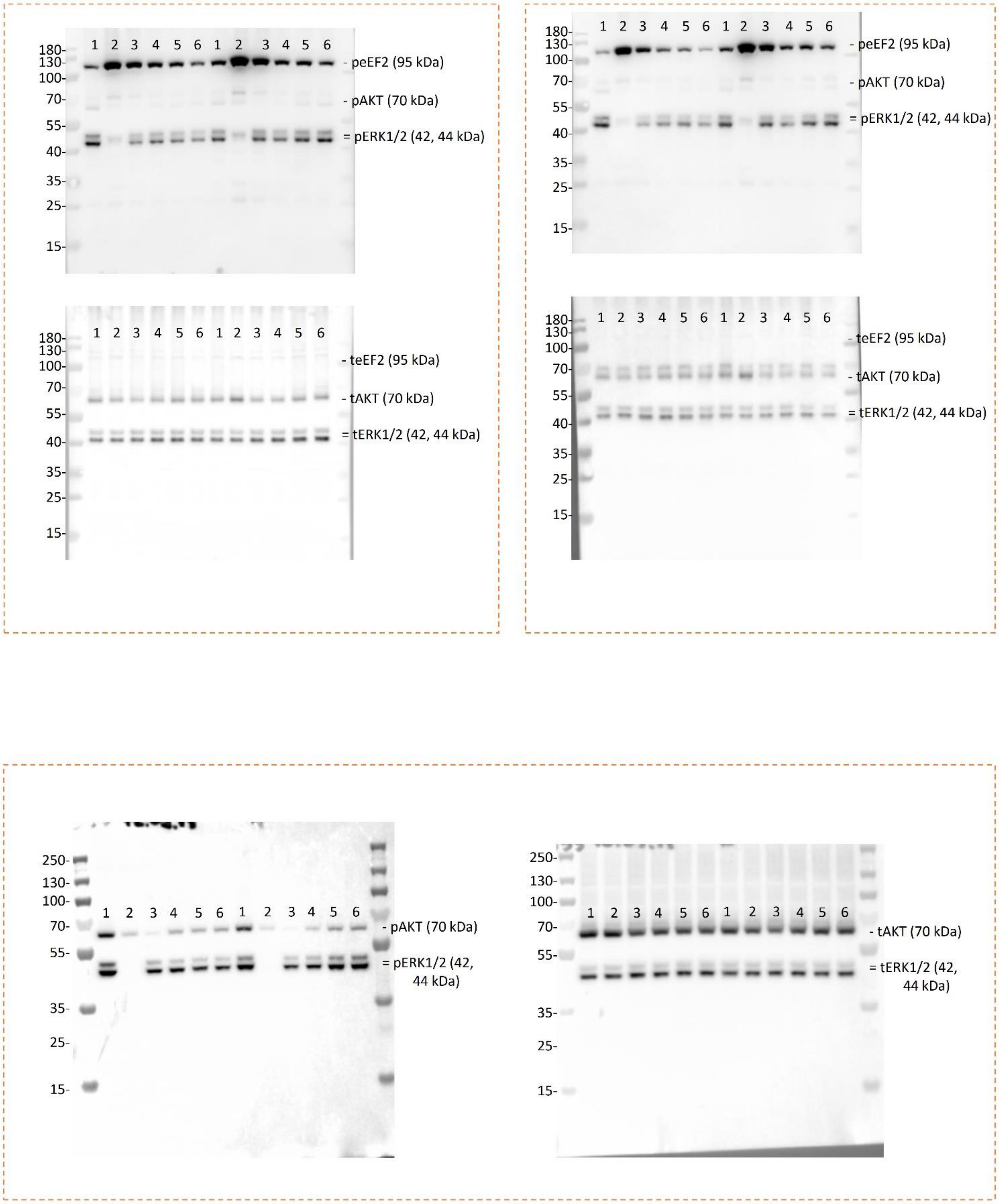

Figure S2C

1. Veh
2. Salmeterol 100 µM
3. Salmeterol 31,6 µM
4. Salmeterol 10 µM
5. Salmeterol 3,16 µM
6. Salmeterol 1 µM

cropped part used for figure preparation

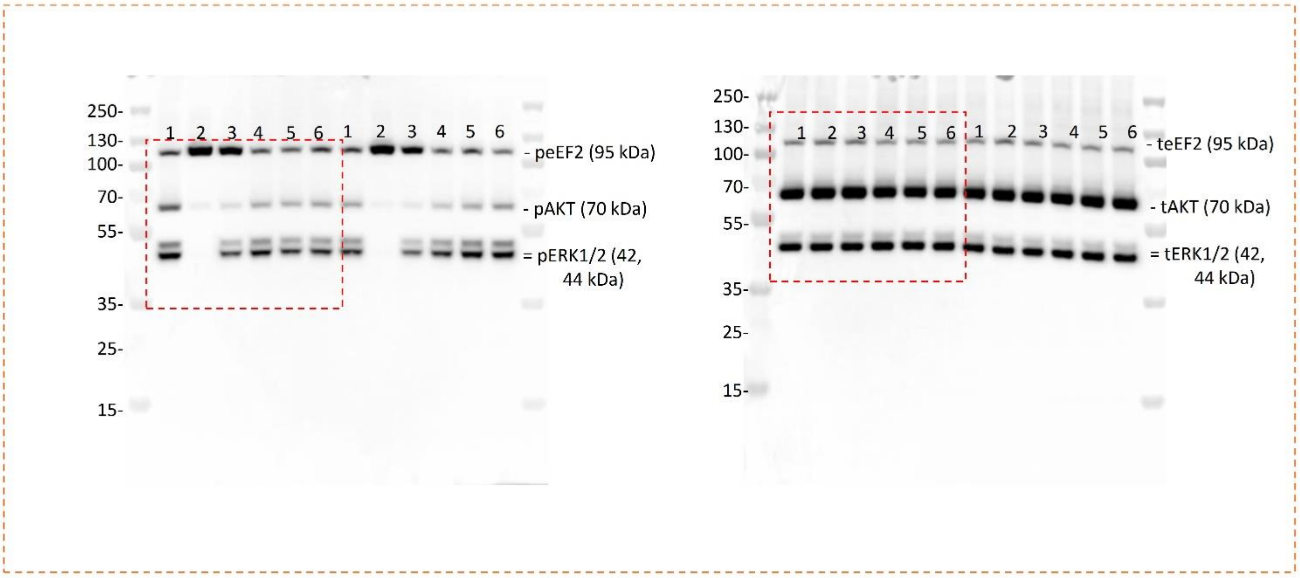

Figure S2E

cropped part used for figure preparation

Lanes:
1. Veh
2. Forskolin 10 µM
3. Forskolin 1 µM
4. Forskolin 100 nM
5. Forskolin 10 nM
6. Forskolin 1 nM

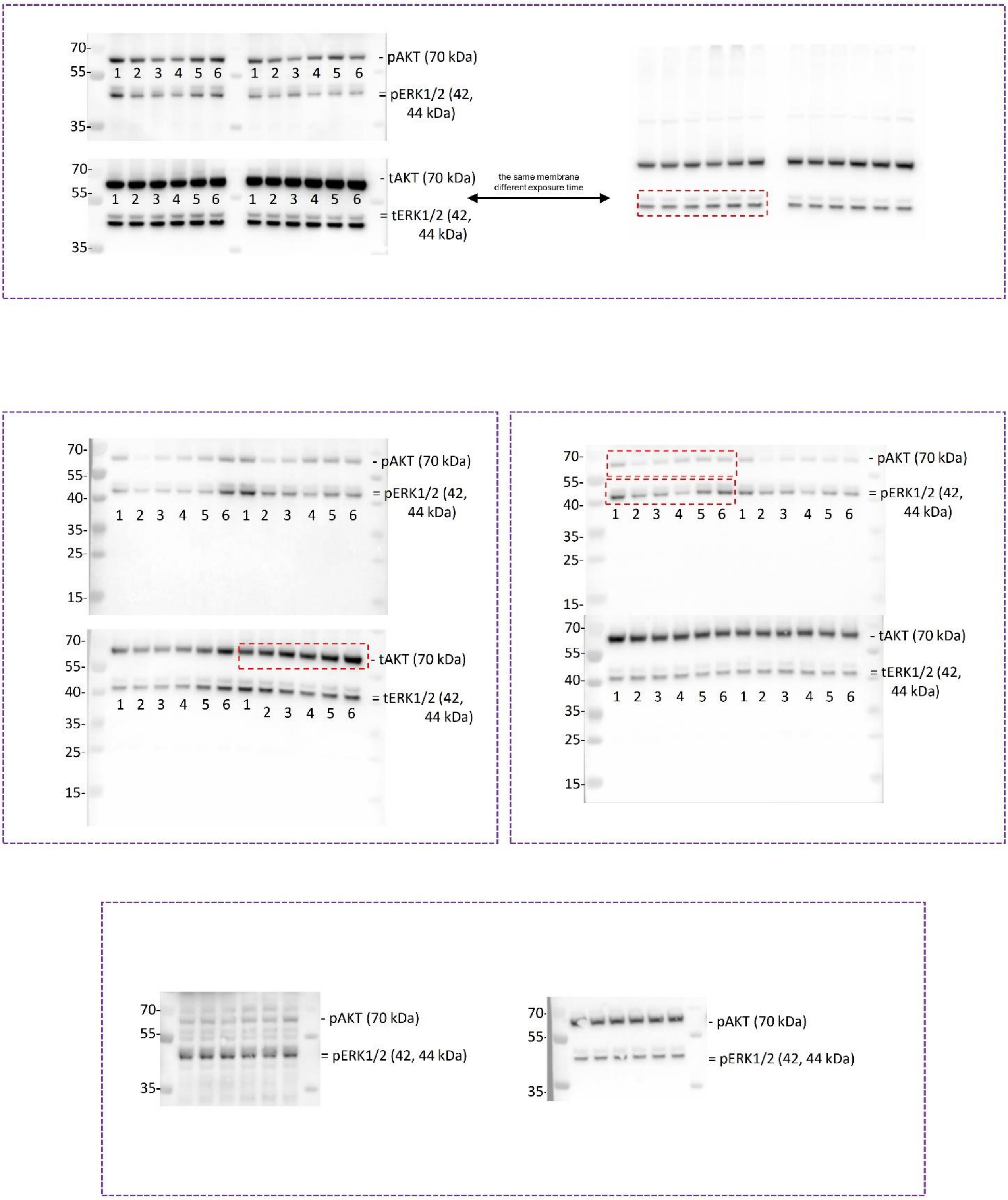

Figure S2E

Lanes:
1. Veh
2. Forskolin 10 µM
3. Forskolin 1 µM
4. Forskolin 100 nM
5. Forskolin 10 nM
6. Forskolin 1 nM

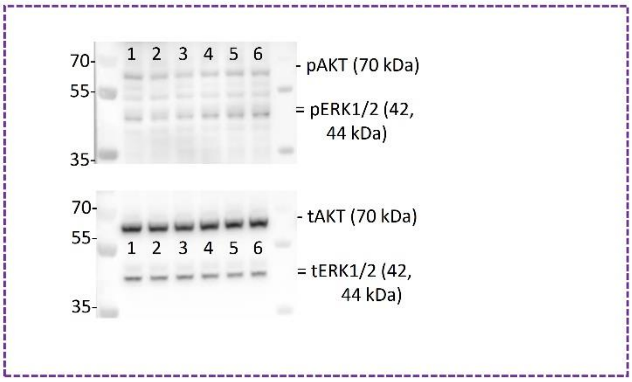

Supplement: Supplementary file 1 — Supplementary Information. [file 41598_2022_7600_MOESM1_ESM.pdf]
